# Delivery Systems for Therapeutic Genome Editing: Challenges, Innovations, and Future Perspectives

**DOI:** 10.1002/mco2.70791

**Published:** 2026-07-12

**Authors:** Meijia Yang, Yiqiong Song, Ziyang Wang, Ke Chao, Lifeng Li, Xu Zhang, Xiaoran Duan, Chenglong Yu, Ruyue Xue, Jie Zhao

**Affiliations:** ^1^ National Engineering Laboratory For Internet Medical Systems and Applications The First Affiliated Hospital of Zhengzhou University Zhengzhou Henan China; ^2^ Out‐Patient Department Armed Police Henan Corps Hospital Zhengzhou Henan China

**Keywords:** delivery systems, genome editing, CRISPR–Cas, AAVs, LNPs, VLPs

## Abstract

Therapeutic genome editing has advanced rapidly with the development of diverse programmable nucleases, from zinc‐finger nucleases and transcription activator‐like effector nucleases to clustered regularly interspaced short palindromic repeats (CRISPR)‐based systems such as base and prime editors. Despite these breakthroughs, clinical translation remains constrained by the challenge of achieving safe, efficient, and tissue‐specific delivery. Viral vectors, particularly adeno‐associated viruses, have enabled durable editing in selected organs but are limited by their restricted cargo capacity, immunogenicity, and complex manufacturing. Nonviral platforms, most notably ionizable lipid nanoparticles, have demonstrated remarkable efficacy for hepatic targets, with clinical trials reporting up to 93% protein knockdown after a single dose. An expanding set of emerging modalities, including virus‐mimicking nanosystems, cell‐derived extracellular vesicles, cell‐penetrating peptides, and intelligent‐responsive multifunctional scaffolds, further enriches the delivery toolbox by supporting transient expression and programmable targeting across diverse editors and tissues. Parallel advances in high‐throughput barcoded screening and machine learning are accelerating vector optimization, while rational chemical modification of payloads improves in vivo stability and specificity. This review provides a comprehensive overview of current and emerging delivery systems for genome editing, highlighting key innovations, unresolved challenges, and interdisciplinary strategies poised to unlock broader therapeutic potential.

## Graphical abstract

Schematic illustration of four emerging CRISPR–Cas delivery platforms defined by distinct design principles and structural features: virus‐mimicking nanosystems (e.g., VLPs), cell‐derived extracellular vesicles, cell‐penetrating peptides, and stimuli‐responsive scaffolds. These platforms enable spatiotemporally controlled delivery of RNPs, mRNA, or DNA for precision genome editing (Figure was created with BioRender.com)

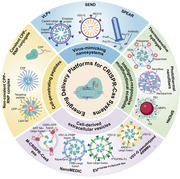

## 1 Introduction

Over five decades have passed since the idea of treating genetic diseases through nucleic acid delivery was proposed. Only in recent years has this concept become practical with the development of programmable genome editing. A key breakthrough was the demonstration that *Streptococcus pyogenes* Cas9 could be directed to almost any genomic locus via a single‐guide RNA (sgRNA). This shifted the field from gene addition toward precise strategies that correct mutations, regulate gene expression, or insert protective alleles [1]. Engineering modifications, such as sgRNA designs and chemical stabilization of tetraloops, have improved potency, stability, and compatibility with delivery systems [2, 3, 4].

The genome editing toolbox has rapidly expanded to compact nucleases such as Cas12f, base editors (BEs) that mediate precise A‐to‐G or C‐to‐T conversions, and prime editors (PEs) that insert new sequences without double‐strand breaks (DSBs), enabling diverse applications [5, 6, 7]. Clinical advances include ex vivo correction of *BCL11A* in hematopoietic stem cells and in vivo delivery of Cas9 messenger RNA (mRNA) by lipid nanoparticles (LNPs), achieving up to 87% reduction of transthyretin in amyloidosis patients [8, 9].

Despite these methodological breakthroughs, delivery remains the main limitation to clinical translation. Effective therapies require editors to reach the correct cells, remain active long enough to achieve durable editing, and do so with minimal toxicity. Genome editors, typically large, are negatively charged biomolecules that cannot traverse biological barriers without assistance. Current clinical strategies therefore rely on a limited set of delivery systems. Recombinant adeno‐associated viruses (rAAVs) remain widely used due to efficient nuclear delivery and favorable tissue tropism. However, their ∼4.7 kb cargo capacity requires dual‐vector or split‐intein strategies for larger editors. Prolonged episomal expression also raises safety concerns such as sustained nuclease activity and immunogenicity responses [10, 11]. In contrast, LNPs provide transient expression and can carry larger cargos such as long mRNAs or ribonucleoproteins (RNPs). Yet their natural hepatic tropism, mediated by apolipoprotein E (ApoE)–LDL receptor (LDLR) interactions, limits applications beyond the liver [12, 13, 14].

Recent studies highlight the strong influence of delivery context. In nonhuman primates (NHPs), a single LNP administration of adenine BEs (ABEs) targeting *PCSK9* achieved 70% editing and 69% LDL cholesterol reduction [15]. However, similar formulations exhibited markedly reduced efficacy in extrahepatic tissues, including cardiomyocytes and neurons [16, 17]. Although directed evolution has produced rAAV variants with improved skeletal muscle tropism in mice, these results often do not translate to primates [18, 19, 20]. Small changes in LNP composition or rAAV cassettes can drastically affect tissue tropism and immune response, underscoring the narrow balance between efficacy and toxicity. The challenge intensifies for sophisticated payloads like clustered regularly interspaced short palindromic repeats (CRISPR)–Cas9, as their guide RNAs are unstable unless chemically protected [4]. Thus, delivery has emerged as the principal bottleneck for therapeutic genome editing. Advances such as baculovirus‐mediated rAAV production, high‐throughput lipid library screening, and viral‐like particles (VLPs) for RNP delivery all aim to improve payload capacity, tissue specificity, expression kinetics, safety, and manufacturing [18, 21, 22]. Only a small fraction of candidates identified in vitro progress to NHP studies, highlighting the difficulty of translation.

This review first outlines the development and limitations of current genome editing technologies. It then discusses the principles of genome‐editing cargo delivery and evaluates established platforms, including rAAVs and LNPs. Emerging delivery systems, such as virus‐like particles and intelligent‐responsive scaffolds, are also examined. Recognizing that no single strategy fits all applications, this review analyses key design challenges and illustrates them with disease‐specific examples. Finally, it highlights future directions, including AI‐guided vector engineering and modular delivery platforms, toward safe and effective genome editing across diverse tissues and diseases.

## 2 Development and Limitations of Genome Editing

It is instructive to consider the entire development trajectory of this field. Genome editing tools have evolved through several stages, from early gene addition strategies to programmable nucleases, and more recently to precision editors such as BEs and PEs. Parallel to these molecular advances, the physical formats used to deliver these editors, including DNA, mRNA, protein, and RNP complexes, have each imposed distinct constraints on in vivo application. Understanding this dual evolution, which involves both the editing tools themselves and their deliverable formats, is essential for appreciating why delivery has emerged as a central hurdle. This section traces the historical development of genome editing, compares the molecular forms available for delivery, and summarizes the major constraints that collectively define the delivery bottleneck.

### 2.1 Historical Evolution of Gene Therapy and Genome Editing

Therapeutic genome manipulation has advanced steadily over the past several decades. A major milestone occurred in 1990, when retroviral vectors were used to introduce exogenous DNA into tumor‐infiltrating lymphocytes for metastatic melanoma therapy, demonstrating that stable genetic modification in human cells was feasible [23]. In 1995, a similar approach was used ex vivo to treat ADA–SCID, restoring immune function and demonstrating clinical benefit [24]. These early successes led to regulatory approvals. China approved Gendicine (adenoviral p53 gene therapy) in 2003. The European Medicines Agency subsequently approved Glybera for lipoprotein lipase deficiency and Strimvelis for ADA–SCID. More recently, rAAV‐based therapies such as Roctavian for hemophilia A have further validated viral vectors as effective clinical platforms.

**FIGURE 1:**
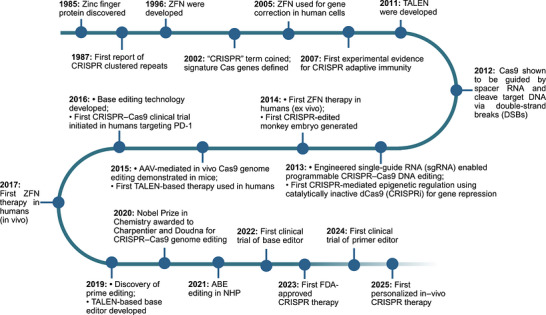
Timeline of key milestones in the development of genome editing technologies. This timeline outlines the development of programmable nuclease platforms—ZFN, TALEN, and CRISPR–Cas9—from foundational discovery to clinical translation. Major milestones include the elucidation of core molecular mechanisms, the first demonstrations of targeted genome modification in mammalian cells and nonhuman primates (NHPs), and the subsequent transition into human clinical trials. More recent achievements highlight the first United States of Food and Drug Administration‐approved CRISPR‐based therapy and the emergence of next‐generation precision editors, such as base editors and prime editors. The awarding of the 2020 Nobel Prize in Chemistry for CRISPR–Cas9 further underscores the transformative impact of these technologies, which continue to shape the landscape of precise genetic intervention. AAV, adeno‐associated virus; ABE, adenine base editor; Cas, CRISPR‐associated; CRISPR, clustered regularly interspaced short palindromic repeats; NHP, non‐human primate; PD‐1, programmed cell death protein 1; TALEN, transcription activator‐like effector nucleases; ZFN, zinc‐finger nucleases. (Figure was created with BioRender.com).

**FIGURE 2:**
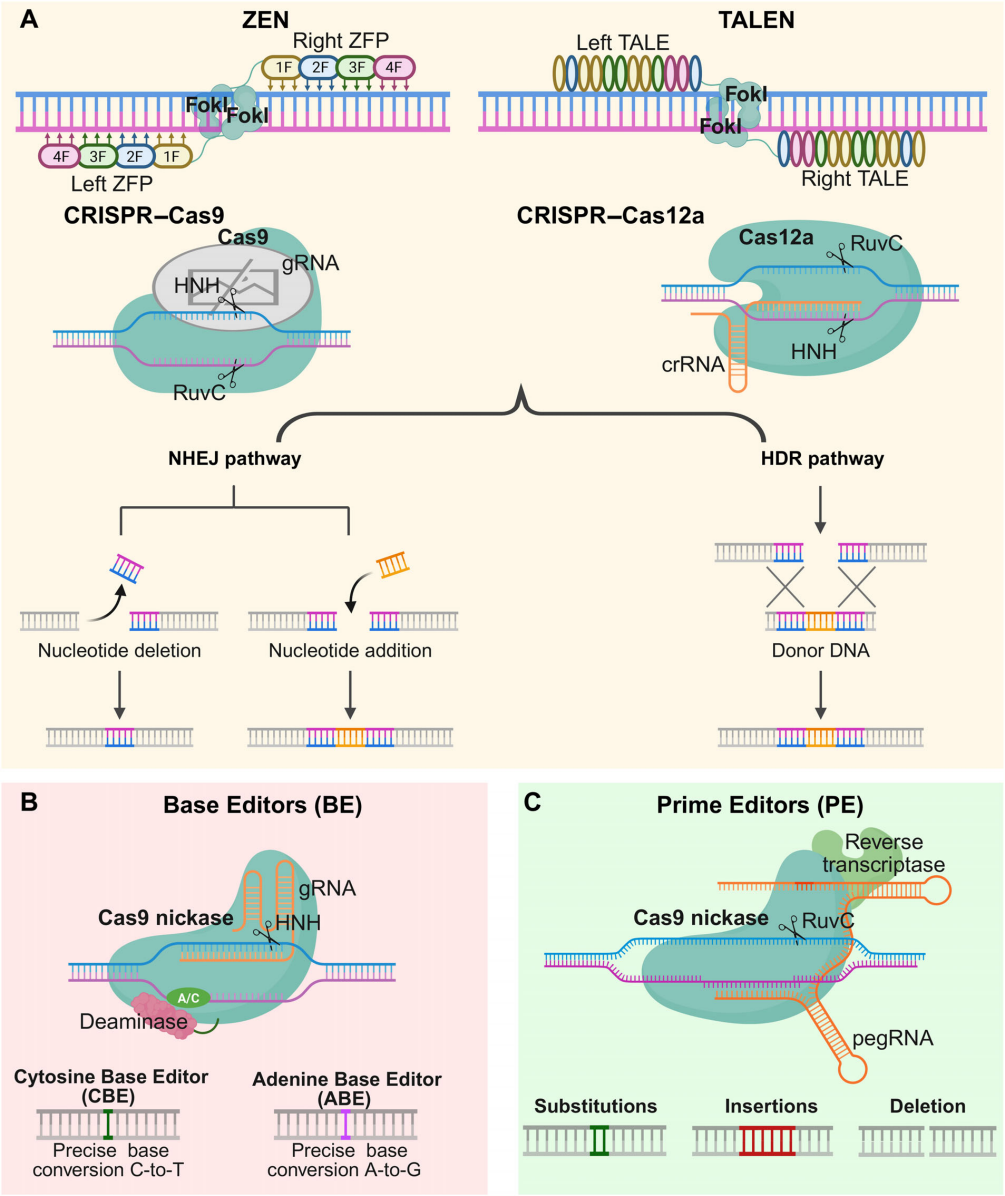
Molecular principles of major genome editing platforms. (A) ZFN and TALEN recognize specific DNA sequences via their protein domains and induce DNA double‐strand breaks (DSBs). The clustered regularly interspaced short palindromic repeats (CRISPR)–Cas9 and CRISPR–Cas12a systems, guided by sgRNA or crRNA, direct Cas proteins to target loci, where the HNH and RuvC domains of Cas9, or the RuvC domain of Cas12a, cleave DNA to generate DSBs. These breaks are predominantly repaired through error‐prone nonhomologous end joining (NHEJ), resulting in insertions or deletions (indels) for gene knockout. In the presence of an exogenous donor template, homology‐directed repair (HDR) can instead introduce precise modifications, including point mutations or gene replacement. (B) Base editors combine a catalytically impaired Cas9 nickase with a deaminase and are directed to target sites by sgRNA, inducing only a single‐strand break (SSB). Without requiring DSB formation or donor DNA, the deaminase catalyzes the chemical conversion of target bases, enabling precise and efficient base substitution. Cytosine base editors (CBEs) mediate C‐to‐T conversion, whereas adenine base editors (ABEs) mediate A‐to‐G conversion. (C) Prime editors comprise a Cas9 nickase, a prime editing guide RNA (pegRNA), and an appended reverse transcriptase (RT). Guided by the pegRNA, the system introduces a targeted SSB and uses the pegRNA‐encoded sequence as a template for reverse transcription. The newly synthesized DNA strand is incorporated into the genome, enabling precise substitutions as well as small insertions and deletions. (Figure was created with BioRender.com.)

Despite these advances, gene addition integrating vectors raised safety concerns such as insertional mutagenesis and uncontrolled gene expression. Many monogenic diseases also arise from point mutations, which require precise correction rather than gene supplementation. These challenges motivated the development of programmable nucleases (Figure 1). Zinc‐finger nucleases (ZFNs) were the first widely adopted and programmable genome editing platform, using engineered zinc‐finger DNA‐binding domains fused to the FokI endonuclease to create DSBs, with their development dating back to 1996 [25]. Although labor‐intensive to design, transient delivery of ZFN mRNA allowed temporally controlled editing with limited toxicity. Transcription activator‐like effector nucleases (TALEN), developed in 2011, simplified design compared with ZFN and offered higher specificity with fewer sequence restrictions [26, 27] (Figure 2A). These enabled efficient ex vivo editing of hematopoietic and immune cells, accelerating progress in cell therapies.

A paradigm shift occurred in 2013 with the adaptation of CRISPR–Cas9. This RNA‐guide system allowed simple reprogramming through a short guide sequence. Fusion of tracrRNA and crRNA into a sgRNA further simplified delivery [28] (Figure 2A), while chemical modifications improved intracellular stability and editing efficiency [2, 3]. Clinical translation followed quickly: in 2016, a first‐in‐human trial used autologous T cells electroporated with Cas9 and an sgRNA targeting PD‐1. These modified cells reduced PD‐1 expression after reinfusion into patients [29]. In vivo editing was soon achieved using LNPs carrying Cas9 mRNA and a TTR‐specific sgRNA, producing an 87% reduction in serum TTR in amyloidosis patients (NTLA‐2001) [9]. At the same time, proprietary Cas9 platforms enabled ∼90% ex vivo editing of hematopoietic stem cells, restoring hemoglobin expression in β‐thalassemia and sickle cell patients in early‐phase trials [8].

Third‐generation editors further expanded the field. Cytosine and adenine BEs, reported in 2016–2017, enabled precise C‐to‐T and A‐to‐G conversions with fewer indels [30, 31, 32] (Figure 2B). Delivery of an ABE8.8 construct using LNPs (VERVE‐101) reduced LDL cholesterol by 69% in NHPs and showed ∼55% reduction in early‐phase clinical trials [15]. PEs, first reported in 2019, integrated a Cas9 nickase, a reverse transcriptase, and a prime editing guide RNA, enabling all base conversions as well as small insertions and deletions [33] (Figure 2C). PEs with hydrodynamic injection or AAV delivery corrected mutations in hereditary tyrosinemia and Leber congenital amaurosis (LCA) mouse models, ameliorating disease phenotypes without off‐target edits, confirming their therapeutic potential [34].

Together, these developments chart the progression from retroviral gene addition to advanced programmable nucleases. Improvements in editing chemistry have proceeded in parallel with advances in delivery systems—from integrating retroviruses to nonintegrating AAVs, and more recently to LNPs and engineered VLPs. This synergistic advancement of molecular tools and delivery technologies has established the foundation for rapid clinical translation of modern genome editing.

### 2.2 Genome‑Editing Modalities: Molecular Formats and Requirements

**FIGURE 3:**
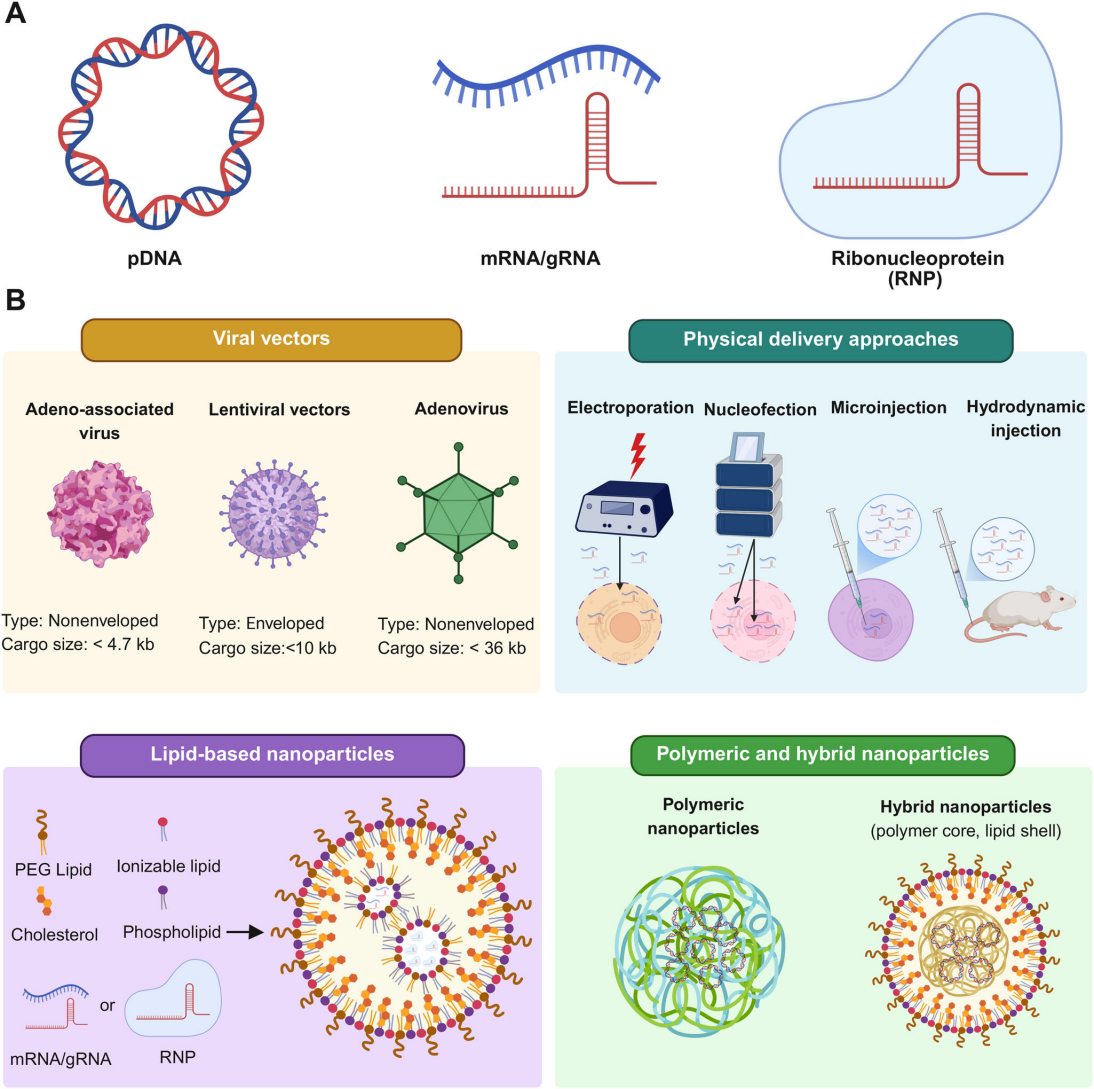
Schematic overview of established delivery methods for genome editing tools. (A) The clustered regularly interspaced short palindromic repeats (CRISPR)–Cas system can be administered in three major formats: plasmid DNA (pDNA), Cas mRNA with sgRNA, or preassembled ribonucleoprotein (RNP) complexes. (B) Four representative delivery platform categories are illustrated: (1) *Viral vectors*: engineered viral systems, including adeno‐associated virus (AAV), lentiviral vectors, and adenovirus, each differing in capsid architecture (enveloped vs. non‐enveloped), genome type, and packaging capacity. (2) *Physical methods*: membrane‐perturbation techniques that enable direct intracellular access, such as electroporation, nucleofection, microinjection, and hydrodynamic injection. (3) *Lipid nanoparticles (LNPs)*: synthetic, self‐assembling nanoparticles composed of ionizable lipids, phospholipids, cholesterol, and PEG–lipids, optimized for the encapsulation and cytosolic delivery of RNA or RNP cargo. (4) *Polymeric and hybrid nanoparticles*: nanoscale carriers including polymeric nanoparticles and hybrid structures (e.g., polymer core with lipid shell), engineered to enhance encapsulation efficiency, stability, and controlled release of genome editing components. (Figure was created with BioRender.com.)

A critical factor in therapeutic genome editing is the physical format in which the nuclease is delivered. Genome editors can be delivered as DNA plasmids, mRNA, purified protein, or preassembled RNPs (Figure 3A). Each format has distinct requirements because of differences in molecular size, charge, stoichiometry, and intracellular localization.

Plasmid DNA is widely used in research because it can encode both the nuclease and its guide RNA on a single construct. However, in vivo use faces major barriers. Cas9 plasmids typically exceed 6 kb, and constructs for BEs or PEs can reach 8–9 kb. Their large, highly charged molecules cross cell membranes inefficiently and require both cytoplasmic transport and nuclear entry before expression begins. Prolonged nuclear exposure to bacterial DNA also activates innate immunity. Furthermore, differences in transcription and translation rates may produce unbalanced nuclease and guide RNA levels, increasing the risk of off‐target genome editing.

Viral cDNAs also require nuclear entry but are more efficient in cellular uptake. AAV vectors are the most clinically validated, but their 4.7 kb capacity excludes most BEs (>5 kb) and PEs. Dual‐AAV systems can split oversized editors but can achieve a maximum of 16% editing efficiency in vivo [35]. Lentiviral vectors (LVs) (∼8.5 kb) and adenoviral vectors (>30 kb) can package larger editors, but the prolonged nuclease expression elevates the risk of genotoxicity. Viral delivery also fixes stoichiometry at the time of infection, while cellular regulation later introduces variability in expression.

mRNA delivery offers distinct advantages by avoiding the requirement for nuclear entry. When released into the cytoplasm, Cas9 mRNA is translated rapidly, enabling transient expression sufficient for stable genome editing while reducing long‐term exposure [36]. In NHPs, codelivery of BEs mRNA and sgRNA by LNP reduced LDL cholesterol by up to 69% [15]. The modular design allows adjustment of mRNA to sgRNA ratios, but both require encapsulation in protective carriers and chemical modifications, such as pseudouridine substitution, to improve stability and reduce immune activation.

Direct delivery of purified protein further accelerates the initiation of genome editing by entirely bypassing the need for translation, offering unmatched temporal precision. However, Cas9 protein is large (∼160 kDa) and aggregates easily in serum, preventing direct uptake. Specialized carriers such as VLPs or cationic lipid carriers are required. Protein delivery also faces the challenge of codelivering sgRNAs, since mismatch in timing reduces efficiency. Preassembled RNP complexes combine nuclease and sgRNA at a defined ratio before delivery. This format supports rapid and transient editing upon nuclear entry, with reduced off‐target effects due to limited intracellular persistence. Studies show that RNP nucleofection yields higher editing efficiency than plasmid or viral cDNA delivery [37, 38]. However, RNPs are larger, highly charged complexes (∼190 kDa total), which require advanced carriers, such as reducible lipidoids, optimized VLPs, or electroporation, to traverse cellular barriers while avoiding degradation.

BEs and PEs further accentuate the disparities among delivery formats. BEs consist of deaminase domains fused to Cas9, enlarging the cDNA by approximately 1 kb and increasing protein mass to ∼200 kDa [31, 32]; PEs, incorporating a reverse transcriptase, exceed 230 kDa and generate cDNA sequences that are incompatible with standard AAV vectors [33]. These expanded cargos further limit compatibility with AAVs and plasmids. In contrast, mRNA and RNP formats can accommodate these larger editors more effectively, provided the stabilization and delivery barriers are addressed.

In summary, the physicochemical characteristics of each delivery format dictate distinct delivery strategies. Plasmid DNA and viral cDNAs offer stable expression but face size limits and sustained expression profiles. mRNA allows rapid and transient expression but requires strong protection from nucleases. Protein and RNPs provide precise control of dosage and expression kinetics but are difficult to transport across membranes. Future genome editing technologies, such as compact nucleases and self‐replicating RNAs, must be evaluated not only for their editing activity but also for the compatibility with delivery systems.

### 2.3 Delivery as the Central Bottleneck

Despite rapid advances in Cas9 nucleases, BEs and PEs, their clinical translation remains constrained by in vivo delivery. Preclinical and early‐phase clinical studies frequently identify four delivery constraints: (1) achieving sufficient target‐cell/tissue accessibility, (2) vector or particle cargo‐size and packaging limitations, (3) immunogenicity of the editing machinery or delivery, and (4) manufacturing, scale‐up and reproducible production under clinical‐grade conditions.

The liver is the most accessible organ for in vivo editing. Both LNPs and rAAVs can achieve high efficiency in hepatocytes. A single 0.3 mg kg^−1^ LNP infusion of NTLA‑2001 reduced serum transthyretin by ∼87% within 28 days [9]. Doses of 0.45–1.5 mg kg^−1^ of the base editing candidate such as VERVE‑101 lowered LDL cholesterol by up to ∼69% [15]. Comparable effects occur in NHPs, demonstrating that therapeutic editing levels above 60% are feasible when access is secured [39]. However, editing efficiencies drop markedly in tissues beyond the liver [40]. AAV9 targeting the adult human central nervous system (CNS) still faces diffusion limits even with intrathecal or intracerebroventricular dosing. These observations indicate that vector tropism, rather than nuclease activity, often sets the ceiling for in vivo editing.

Cargo size imposes a further constraint that is frequently underestimated. The ∼4.7 kb rAAV capacity is insufficient for accommodating full‐length SpCas9 with regulatory elements, and many BEs and PEs exceed this limit [41, 42]. Dual‐AAV split systems partly address the issue but commonly produce limited editing efficiency in vivo and add cotransduction variability [35, 43]. Oversized disease targets (e.g., CEP290) for LCA (∼7.5 kb) and full prime editing systems (>5 kb) require splitting or nonviral delivery. LNPs bypass capsid limits, but higher nucleic acid loads can destabilize particles and increase innate immune activation.

Immunogenicity of the delivery vehicle constitutes a third major barrier. Many adults harbor neutralizing antibodies (NAbs) to common AAV serotypes, restricting enrollment and redosing, and high vector doses have been linked to complement‐mediated and hepatic toxicities [44, 45]. LNP‐based systems also encounter immune‐related challenges: infusion reactions require management and transient transaminase elevations occur. Because Cas enzymes are bacterial in origin, prolonged expression risks adaptive immune responses, which favors transient delivery formats such as mRNA or RNPs [46, 47].

Manufacturing constraints on clinical‐grade vectors constitute a fourth critical limitation. rAAV production is limited by assembly inefficiencies, long lead times, and high cost, constraining dose exploration and redosing strategies. Similarly, LNP manufacturing faces a cross‐species translation challenge: many formulations that perform in mice or NHPs lose potency or specificity in humans. Regulators now require expanded data on off‐target effects and potential germline exposure, lengthening timelines.

Collectively, these findings show that improvements in nuclease specificity and sgRNA chemistry are no longer the main limitation. The bottleneck lies in the delivery systems themselves. Safe, efficient, and scalable delivery is essential for expanding genome editing beyond the liver and into diverse therapeutic contexts. Nowhere is this challenge more pronounced than in oncology, where the tumor microenvironment introduces additional physiological barriers that necessitate specialized delivery strategies. Ghaemi and colleagues have provided a comprehensive review of CRISPR–Cas9 delivery systems engineered for cancer therapy, highlighting how platforms such as chitosan‐based nanocomplexes, gold nanorods, and pH‐responsive polymeric nanoparticles are designed to enhance tumor‐specific accumulation and efficacy [48]. Their analysis emphasizes the critical importance of tailoring delivery strategies to disease‐specific features, thereby offering a valuable framework for next‐generation cancer therapies.

## 3 Principles of Genome‑Editing Cargo Delivery

Effective genome editing requires that therapeutic cargos overcome a sequence of biological barriers before reaching the nucleus. At the systemic level, exogenous nucleic acids and proteins are rapidly degraded or cleared, while nanoparticles interact with serum proteins that alter their pharmacokinetics. Particle size and charge strongly influence their fate: particles smaller than 10 nm are rapidly cleared by the kidney, whereas those above 200 nm trigger immune clearance. A size range of 50–150 nm usually balances circulation with uptake [49, 50]. At the tissue level, physical barriers further restrict distribution. The blood–brain barrier (BBB) excludes most nucleic acids and larger complexes, requiring intrathecal dosing, viral capsids with BBB penetrance, or engineered nanoparticles that engage receptors such as transferrin [51, 52, 53]. Similar barriers exist in fibrotic stroma, tumor interstitium, and bone marrow sinusoids, where dense or specialized environments limit extravasation [54, 55]. After reaching the target tissue, efficient cellular internalization becomes crucial. The cargo format dictates distinct requirements. mRNA enables rapid but transient expression yet demands stabilization, and RNPs provide immediate activity with minimal persistence but need strong protection and efficient endosomal escape. Endosomal escape remains a major bottleneck, with typically <2% of internalized material reaching the cytosol [56, 57]. DNA requires nuclear transport and sustained expression. Nuclear import presents a significant challenge for DNA‐based editors. This is because plasmid dimensions exceed the size exclusion limit of nuclear pore complexes, preventing passive diffusion. Consequently, their nuclear entry often depends on transient nuclear envelope breakdown during mitosis or must be facilitated by specialized viral mechanisms [58].

In summary, genome‐editing cargos face multilayered physiological defenses spanning circulation, tissue entry, cellular uptake, and the nuclear envelope. No single platform universally overcomes all barriers. Instead, effective delivery requires integrated engineering that balances stability, tropism, and intracellular trafficking. These principles provide the foundation for the delivery platforms discussed in the following sections.

### 3.1 Barriers from Injection Site to Target Tissue

Systemic delivery of genome‐editing cargos encounters multiple extracellular barriers that significantly reduce efficiency before the RNPs, mRNA, or plasmid DNA reach their chromosomal target. These include nucleases degradation, renal and hepatic clearance, phagocytic uptake, endothelial filtration, and steric or electrostatic hindrance by the extracellular matrix (ECM). Such challenges have motivated the development of lipid, polymeric, and membrane‐based carriers designed to stabilize and transport CRISPR cargos in vivo.

Serum endonucleases represent a major degradation pathway [59]. Chemical modification of oligonucleotide backbones, such as phosphorothioate (PS) linkages, enhances resistance to nucleases [60], although they reduce duplex stability [61]. Alternative chemistries, including phosphonoacetate, thiophosphonoacetate, and phosphoryl guanidine (PG) linkages, offer tailored balances among nuclease resistance, RNase H recruitment, and cellular uptake [62, 63, 64]. Formulation strategies, such as encapsulation within LNPs, provide physical shielding against nucleases and complement proteins until endosomal escape [65, 66].

Particle size and charge are also key determinants of circulation and clearance. Nanoparticles smaller than ∼10 nm are rapidly filtered by the kidney, whereas those larger than 200 nm tend to trigger immune activation. An optimal range of 50–150 nm supports extended circulation while maintaining tissue penetration [49, 50]. PS modifications enlarge the hydrodynamic radius through albumin binding, thereby reducing renal clearance. Polyethylene glycol (PEG) coatings and hydrophilic coatings further improve colloidal stability and reduce aggregation, while low zeta potential prevents nonspecific interactions [67, 68].

The mononuclear phagocyte system presents an additional challenge, particularly opsonization and clearance [69]. To circumvent this, carrier surfaces are engineered to resist protein adsorption. Hydrophilic polymer shells, such as dense PEG layers, can reduce protein adsorption and suppress macrophage uptake [70, 71]. Natural vesicle systems, such as exosomes, exploit endogenous membranes to evade immune recognition and enable codelivery of Cas9 and sgRNA [72, 73]. Upon evading systemic clearance, nanoparticle formulations traverse the endothelial barrier separating the vasculature from target cells. ApoE binding to LNP surfaces enables LDLR‐mediated uptake by hepatocytes [74, 75]. Adjustment of helper lipid charge can redirect tropism: cationic helper lipids (e.g., DOTAP) impart a positive surface charge to LNPs, shifting tropism from the liver to the lungs and correlating with reduced pulmonary immune cell infiltration. Conversely, anionic helper lipids (e.g., phosphatidylserine) reduce the zeta potential, redirecting LNPs to the spleen and potentiating transfection in splenic immune cells [76]. Beyond the vasculature, the ECM constitutes a dense and heterogeneous barrier that restricts nanoparticle diffusion, especially in tumors with abnormal vascularization, acidic pH, and elevated reactive oxygen species (ROS) [77, 78]. These stimuli‐responsive carriers, such as disassembling LNPs, take advantage of these pathological microenvironments to trigger selective release [79, 80]. Collectively, advances in chemical modification, lipid chemistry, and biomimetic engineering provide complementary strategies to overcome systemic barriers and enable effective delivery.

### 3.2 Cellular and Intracellular Barriers

Efficient therapeutic genome editing requires the successful transport of nucleic acid or protein cargos across sequential intracellular barriers, from initial plasma membrane entry to final localization in the nucleus or mitochondria. Each barrier imposes a bottleneck that reduces the effective intracellular dose. Modern delivery platforms are therefore engineered to coordinate membrane binding, cellular uptake, endosomal escape, cytosolic transport, and, when needed, active nuclear import [81].

Viral vectors offer highly adapted solutions to these processes, with rAAV being the most classic example. rAAV particles first bind to cell‐surface glycans and receptors, enter via clathrin‐mediated endocytosis [82]. Within early endosomes, pH‐induced conformational changes promote downstream trafficking [83]. Capsid–host interactions, most notably with AAVR and GPR108, facilitate endosomal escape and direct transit through the trans‐Golgi network toward the perinuclear region [84, 85, 86]. Importantly, N‐terminal motifs in VP1 and VP2 interact with the nuclear pore complex, enabling nuclear import of the single‐stranded DNA genome [87]. Subsequent second‐strand synthesis converts this genome into a double‐stranded, transcriptionally active form, a process accelerated by mutations in internal terminal repeats [88]. This inherent nuclear tropism supports robust transgene expression. For example, dual rAAV have successfully delivered PEs and achieved 38% editing at the *Dnmt1* locus in mouse liver [89].

Nonviral vectors orchestrate intracellular trafficking processes through distinct physicochemical mechanisms. For instance, LNPs rely on their ionizable lipid components, which undergo protonation in the acidifying endosomal environment to disrupt membrane integrity and enable efficient cytosolic release [83]. Cationic polymers such as polyethyleneimine (PEI) and poly(β‐amino ester)s (PBAEs) form polyplexes that exploit the proton‐sponge effect to disrupt endosomes. Structural modifications, including guanidinium substitution, further strengthen membrane interactions [90, 91]. Although these strategies successfully address the critical bottleneck of endosomal escape for DNA cargos, nuclear import in nondividing cells remains a key limitation. Approaches such as nuclear localization signals or coupling to microtubule transport machinery have been investigated to enhance delivery [92, 93].

Preassembled RNPs combine nuclease and sgRNA at fixed stoichiometry, offering rapid and transient activity with reduced off‐target effects. However, their large hydrodynamic size and high charge complicate in vivo delivery [94, 95]. Electroporation remains effective ex vivo, particularly for stem cells and zygotes, but in vivo toxicity restricts its use. Alternative approaches include exosome‐mediated fusion, which bypasses endocytosis, and VLPs, which encapsulate BEs and PEs to minimize nuclease exposure while maintaining delivery efficiency [96, 97].

These platforms integrate multiple mechanisms into a single carrier to coordinate uptake, endosomal escape, cytosolic release, and nuclear import in sequence. For instance, phenylboronic acid‐modified PEI nanoparticles carrying dCas9 plasmids are cloaked with a pH‐sensitive PEG shell; in the acidic tumor microenvironment, the shell detaches to expose boronic acids, which bind sialylated glycoconjugates and enhance uptake. Following endosomal internalization, the proton sponge effect of PEI drives endosomal rupture, enabling DNA release into the cytosol, while PEI's intrinsic nuclear affinity facilitates nuclear import [98]. Related designs using pH‐responsive dendrimers and hydroxyl‐rich lipid systems undergo stepwise disassembly in response to local biochemical cues, synchronizing intracellular trafficking and improving overall delivery efficiency [99].

Intracellular barriers thus impose stringent requirements on delivery platforms. rAAVs remain unmatched for efficient nuclear entry, while LNPs and polymers provide modularity and reduced immunogenicity but are limited by poor endosomal escape. RNP‐based and vesicle‐derived systems offer precise temporal control but face significant challenges in stability and intracellular trafficking. Continued innovation across these platforms is required to address these persistent obstacles and enable safe, efficient, and tissue‐specific genome editing.

### 3.3 Impact of Cargo Form on Delivery Strategy

The physical format in which genome‐editing agents are delivered, such as plasmid DNA, mRNA, or preassembled RNP, directly determines their requirements for systemic protection, intracellular trafficking, and stoichiometric balance of components. Each format imposes distinct design principles for vector engineering in therapeutic applications.

Plasmid vectors must translocate large, highly charged macromolecules across both cytoplasmic and nuclear membranes and sustain transcription long enough to achieve robust editing. Plasmid constructs allow stoichiometric coexpression of Cas proteins and gRNAs from a single transcriptional unit for editing and immune activation. Yet, extended or high‐level expression elevates risks of off‐target editing and immune activation. Capsid engineering has addressed this by reducing required DNA doses [21, 100]. For example, re‐engineered Padua FIX levels mitigate risks associated with sustained DNA exposure [101]. Viral vectors such as AAV and lentivirus are frequently employed because their capsids provide strong protection against serum nucleases and actively facilitate nuclear import. However, their limited packaging capacity constrains their applicability [41, 102]. PEs are approximately 6 kb, requiring dual‐AAV strategies in which split‐intein halves are separately packaged and delivered [43]. Dual‐AAV8 has achieved 15% editing efficiency at the Dnmt1 locus in mouse liver, proving in vivo feasibility, though with added manufacturing and dosing complexity [103].

mRNA‐based delivery strategies prioritize rapid cytosolic release and transient expression. The inherent instability and large size of mRNA necessitate protective carriers that also promote efficient endosomal escape. Ionizable LNPs dominate this field: they condense mRNA at acidic pH and trigger cytosolic release [104]. Nucleoside modifications such as pseudouridine and 5‐methylcytidine further improve transcript stability and reduce innate immune activation [105]. Clinically, VERVE‑101, an LNP coencapsulating SpCas9 mRNA and stabilized gRNA, produced durable A‐to‐G editing in PCSK9, lowering LDL cholesterol by 69% at single doses of 1.5 mg kg^−1^, with effects sustained for up to 476 days in NHPs [15]. Success relies on delivering Cas mRNA and gRNA in stoichiometric ratios. Coencapsulation achieves this, but the mass of Cas mRNA exceeds sgRNA by nearly two orders of magnitude, complicating equimolar delivery at the cellular level. Advances such as zwitterionic amino lipids and selective organ targeting (SORT) lipids have expanded editing beyond the liver by fine‐tuning endosomal escape and biodistribution, highlighting how lipid chemistry can reconcile protection with efficient cytosolic access [106].

Direct delivery of RNP bypasses transcription and translation, minimizing nuclease exposure and reducing off‐target activity. Ex vivo, electroporation of RNPs is widely used for stem cells and zygotes. In vivo, recent advances using electrically neutral or mildly cationic LNPs demonstrate effective packaging and delivery of intact RNPs to achieve in vivo homology‐directed repair (HDR) [107]. However, RNP payloads are volumetrically larger than RNA and present less uniform charge distribution than DNA, which imposes stricter limitations on vector size and surface characteristics. Alternatively, biologically derived carriers such as exosomes and VLPs have shown promise. Exosomes retain parental membrane proteins that promote direct fusion with target cells, thereby facilitating cytosolic release and reducing dependence on endocytosis. For example, liver‐derived exosomes loaded with dCas9–VP64 successfully reprogrammed hepatic stellate cells and alleviated fibrosis in mice [108]. VLPs, structurally similar to retroviruses but replication deficient, have been used to package and deliver mature RNPs within target cells [109]. Fourth‐generation VLPs have reached high editing efficiencies in mice through optimization of cargo localization signals and precise tuning of the Gag:protease:RNP stoichiometry, maximizing encapsulation while minimizing premature cargo release [110].

The distinct release profiles required by each form further refine vector selection. DNA editors benefit from slow unpacking and, when applicable, integration, which supports nuclear entry and sustained expression; vectors with slow disassembly kinetics are therefore favored. mRNA editors require rapid cytosolic exposure and favor vectors that destabilize in mildly acidic endosomes. RNP editors have short intracellular half‐lives and demand near‐instantaneous release, motivating vectors that undergo triggered disassembly via mechanisms such as reductive cleavage or protease activation. Protective strategies also differ by cargo type: DNA commonly employs capsid proteins or dense polymer matrices to shield against nucleases; mRNA leverages ionizable lipids and nucleoside modifications; RNPs require concurrent stabilization of both protein and RNA, consequently often achieved through neutral lipid encapsulation or VLP‐based scaffolding.

Taken together, a given genome editor defines not a single therapeutic entity but three distinct cargos: DNA, mRNA, or RNP, each imposing unique physicochemical requirements that govern vector design. Rational vector selection must therefore account not only for tissue specificity and manufacturability, but also for cargo‐dependent protection, release kinetics, and stoichiometry. As delivery platforms advance, modular systems, including switchable LNP chemistries and programmable VLP scaffolds, offer adaptable solutions to these constraints, broadening the clinical applicability of genome editing.

## 4 Established Delivery Platforms in Clinical and Late Preclinical Use

Vectors that have advanced from preclinical studies to first‐in‐human dosing provide an instructive base for next‐generation genome‐editing design. Three platforms dominate this landscape: rAAV, LNPs, and ex vivo electroporation of RNPs, each revealing specific constraints and opportunities that guide vector selection and engineering [111]. Among viral vectors, rAAV remains the most deeply characterized in vivo vector, with durable transgene expression and well‐mapped tissue tropism supporting applications across multiple organs. LNPs offer a complementary route based on transient expression with manufacturing processes that already scale for clinical use [112, 113]. LNPs achieve high efficiency in hepatocytes through ApoE–LDLR interactions and have produced therapeutically relevant levels of editing in clinical settings [75, 114]. Ex vivo electroporation of preassembled RNPs has delivered high on‐target editing of BCL11A in autologous CD34^+^ cells and corresponding restoration of fetal hemoglobin (HbF) in haemoglobinopathies [115, 116]. These platforms each exhibit distinct advantages and limitations in terms of editing efficiency, delivery capacity, immunogenicity, and applicability (Table 1), thereby guiding the rational selection and engineering of vectors (Figure 3B).

**TABLE 1:** Established delivery platforms comparison for genome editing.

Delivery platforms	Details	Editing efficiency	Advantage	Disadvantage	References
Adeno‐associated virus (AAV)	Single‐AAV‐encoded ABEs injected in mice	Up to 66, 33, and 22% editing in liver, heart, and muscle, respectively	− High infection efficiency − High safety − Low immunogenicity − Multiple serotypes for flexible targeting − Minimal genomic integration risk − Transduces dividing and nondividing cells	− Limited packaging capacity − High production cost − Pre‐existing antibodies limit application − Capsid immunogemocity − Risk of liver toxicity at high doses − Long‐term expression increases off‐target risk	[117]
	AAV‐delivery of SaCas9 for in vivo genome editing	>40% (*Pcsk9* knockout in the mouse liver)			[118]
	Self‐complementary AAV (scAAV) to deliver RNP into a mouse model of DMD	Up to 30% in muscle (*Dmd* exon 45 knockout in the triceps)			[119]
	All‐in‐one self‐cleavage AAV‐CRISPR–Cas9 system to limit the long‐term expression of Cas9 nuclease	Without compromising on‐target efficacy in vitro and in vivo			[120]
	AAV2–SaCas9–gRNA targeting *GFP* in HT1080 single‐cell clone	∼80% GFP knockout efficiency			[121]
	AAV‐mediated delivery of SaKKH–CBE3 into *Pah* ^enu2^ mice	∼23% on‐target editing (in whole liver lysates)			[122]
	Dual AAVs for the delivery of split cytosine and adenine base editors in mice	Up to 59% in brain, 38% in liver, etc.			[89]
	Dual AAV‐mediated adenine base editing to correct the most common mutation in *ABCA4*	∼75% in cones, ∼87% in RPE cells			[123]
	Dual AAVs (v3em PE‐AAV) for prime editing to install mutations of biomedical interest in mice	Up to 42% in brain, 46% in liver, 11% in heart			[124]
Lentiviral vectors (LVs)	Single lentiviral vector (LV‐X4R5) delivering Cas9 and dual sgRNAs targeting *CXCR4* and *CCR5*	∼55% for *CCR5* and ∼36% for *CXCR4* in CD4+ cell lines	− High transduction efficiency − Large packaging capacity − Low immunogenicity − Broad cell tropism − High cloning capacity − Long‐term transgene expression − Inexpensive production	− Potential integration and insertional mutagenesis risk − Potential carcinogenicity − Limited in vivo delivery efficiency − Complex manufacturing and scale‐up challenges − Prolonged expression leads to risk of off‐target editing	[125]
	Lentivirus for the delivery of Cas9 and gRNA targeting *MYOC* in human TM3 cells	∼62% reduction in mutant MYOC accumulation			[126]
	Integrase‐defective lentiviral vectors delivering ZFNs and template DNA for gene correction at *IL2RG* locus	13–39% editing across different cell types			[127]
	Integrase‐deficient lentiviral vectors delivering CRISPR/Cas9 for targeted *GALNS* knock‐in in MPS IVA mice	HDR efficiency: 5.58–12%; Indel formation: 24.6–26.8%			[128]
	Engineered lentiviral vectors for the delivery of Cas9 and gRNA targeting *Vegfa*	∼44% knockout editing efficiency			[129]
Adenovirus (Ad) and hybrid Ad/AAV systems	High‐capacity Ad vectors delivering CRISPR/Cas9 system containing three gRNAs against HBV	∼54% reduction of HBsAg secretion	− Efficient delivery efficiency − Large packaging capacity − Minimal genomic integration − Can transduce dividing and nondividing cells − High transient expression efficiency	− High immunogenicity − Pre‐existing antibodies limit readministration − Potential cytotoxicity − Prolonged expression leads to risk of off‐target editing − Hybrid system still has limited capacity	[130]
	High‐capacity Ad vectors type 5 delivering Tet‐on‐inducible CRISPR/Cas9 and donor DNA for HDR in Huh7–cFIXmut cells	Up to 5.52% HDR efficiency			[131]
	High‐capacity Ad vectors delivering CRISPR/Cas9 system for major *DMD* hotspot deletion	Up to 42 ± 13% of alleles with targeted deletion			[132]
	High‐capacity Ad vectors delivering forced CRISPR–Cas9 heterodimers for *DMD exon 51* deletion in human myoblasts	∼1.7‐fold increase in precise deletions; precision index improved by ∼3–4‐fold			[133]
	HDAd5/35++ vectors delivering CRISPR/Cas9 and donor DNA for HDR in AAVS1 transgenic mice	Up to 97% γ‐globin+ RBCs in individual mice; ∼24% γ‐globin of total adult mouse β‐globinc			[134]
	Combining single‐stranded AAV delivery of HR donors with third‐generation Ad vectors transfer of CRISPR–Cas9 nucleases for knock‐in in HeLa cells	97.6–100% precise HDR among targeted clones			[135]
	Combining AAV donor with Ad‐TALENs for COL7A1 mutation correction in RDEB–E67A6 cells	∼39% targeting efficiency			[136]
Physical methods	Electroporation of ABE8e–NRCH mRNA + sgRNA into human CD34^+^ HSPCs from SCD patients	∼80% HBB^S^‐to‐HBB^G^ conversion	− High efficiency − Direct delivery, no carrier limitations − Widely used − Dosage more controllable − Suitable for various cell types	− Need specialized equipment − Low throughput (e.g., Microinjection) − Significant cell damage − Difficult for in vivo application − High technical skill required − Nonpersistent gene expression	[7]
	Tube electroporation of Cas9/gRNA RNP + ssODN targeting APP in human iPSCs	Up to 42.1% HDR efficiency			[137]
	Electroporation of Cas9 mRNA + gRNA + ssODN against *Il2rg* into intact rat embryos	Up to 88% knockout efficiency, 33% knock‐in efficiency			[138]
	Nucleofection of Cas9 mRNA into human T cells for *TRAC* and *CD52* knockout to generate universal CAR19 T cells	CAR19 expression (>97%), residual TCR (<1%), and CD52 negativity (>70%)			[139]
	Nucleofection of Cas9/gRNA RNP into primary cells	>90% indel efficiency			[140]
	Microfluidic droplet cell mechanoporation for RNP delivery into K562 cells	∼62.16% knockout editing efficiency			[141]
	Microfluidic‐assisted biomineralization for RNP delivery with NIR‐triggered release in HeLa/GFP cells	∼80% GFP knockout efficiency			[142]
	Microinjection of sgRNA and Cas9 mRNA into the yolk of 1‐cell stage zebrafish embryos for germline transmission	∼28% germline transmission efficiency			[143]
	Microinjection of CRISPR/Cas9 RNP into fresh mouse zygotes	Mutation rate in pups: 73.7% (by PAGE)/89.5% (by Sanger sequencing)			[144]
Lipid‐based nanoparticles (LNPs)	LNP‐delivered CRISPR base editor targeting the *PCSK9* gene in nonhuman primate	Mean liver editing of 46% at 0.75 mg kg^−1^ and 70% at 1.5 mg kg^−1^ dose	− High biocompatibility − Low immunogenicity − No risk of integration − All‐in‐one delivery − Low cost, easy scale‐up − Feasible of in vivo application	− Delivery efficiency varies by cell type − Limited payload capacity − Poor in vivo circulation stability − Dose‐dependent toxicity − Significant liver accumulation − Requires formulation optimization − Batch‐to‐batch consistency challenges	[15]
	LNP formulated with novel ionizable lipid HTO12, codelivering ABE8.8m mRNA and sgRNA targeting the *Pcsk9* gene in mice	∼63.4% editing in liver			[145]
	LNP formulated with C9‐200 ionizable lipid, codelivering Cas9 mRNA and TTR sgRNA in mice	∼35% editing in liver			[146]
	Peptide–ionizable lipid nanoparticles (PILOT LNPs) codelivering PEmax mRNA and epegRNA for prime editing in mice	∼13.1% editing in liver			[147]
	Enhanced lipid nanoparticles (eLNPs) containing the cholesterol analog β‐sitosterol, codelivering prime editor mRNA, pegRNA, and nsgRNA for prime editing in a reporter cell line	∼54% prime editing rate			[148]
	Cationic lipid nanoparticle delivering pCas9–sgLDHA plasmid DNA to edit *GFP* gene in HeLa–GFP cells	Up to 31.3 ± 2.3% indel efficiency			[149]
	ALC‐0315 ionizable cationic lipid LNP delivering ABE8e mRNA and LucR387X‐specific gRNA for base editing in a luciferase reporter mouse model	Up to 24.6 ± 5% base editing efficiency			[150]
Polymeric nanoparticles	Lonic liquid‐conjugated polymers with fluorination monomers (PBF) for delivery of CRISPR/Cas9 plasmid in various cell lines and in vivo tumor models	∼41.2% *PLK1* gene knockout in vivo	− High structural designability − Minimal immunogenicity − All‐in‐one delivery − Flexible preparation methods − Easy large‐scale production − Low cost	− Relatively low delivery efficiency − Variable biocompatibility and toxicity − Targeting depends on modification − Possible immune response	[151]
	PEG‐b‐PLGA/PEI nanoparticles delivering CRISPR–Cas9 plasmid with CDH5 promoter for endothelial‐specific genome editing in adult mice	∼13–39% indel efficiency			[152]
	Precise PBAE with guanidine end group (guanidine‐4mer) delivering pDNA encoding ABEmax‐NG in HEK‐293T–sEGFP cells	∼12% prime editing rate			[153]
	Poly(beta‐amino ester)‐based nanoparticles delivering ABEmax‐NG/sgRNA plasmid to HEK‐293T–sEGFP cells in serum‐free medium	Up to 70% EGFP^+^ cells (under the serum‐free condition)			[154]
	Cyclic poly(β‐amino ester)s (CPAE‐2A) delivering CRISPR–EX80 plasmid for *COL7A1* exon 80 deletion in RDEBK cells	∼60% knockout efficiency			[155]
	highly branched PBAEs (HPAEs) with PG groups (HPAE‐PG) delivering dual CRISPR RNP for *COL7A1* exon 80 deletion in HEK cells	∼32% exon 80 deletion efficiency			[156]
Hybrid nanoparticles	Lipid–polymer hybrid nanoparticles for compartmentalized delivery of Cas9 protein and sgRNA targeting *STAT3* in glioblastoma	∼49.62% *STAT3* knockout efficiency	− High biocompatibility − High structural design flexibility − Strong nucleic acid protection − Potential for targeted delivery	− Complex fabrication process − Limited delivery efficiency − Potential immunogenicity − Stability needs optimization	[157]
	Glutathione (GSH) hybrid PBAE–plasmid nanoparticles delivering CRISPR/Cas9 plasmids targeting *KAT6A* in HEK 293T cells	∼10.7% indel efficiency			[158]
	Ionizable lipopolymer–lipid hybrid nanoparticles delivering CRISPR–Cas9 mRNA/sgRNA to lung tissue	Up to 5.6% ± 2.4% indel efficiency			[159]
	Lipid–polymer hybrid nanoparticles delivering CRISPR/Cas9 plasmids targeting MGMT in T98G glioblastoma cells in vitro	Significant downregulation of MGMT protein expression			[160]

Abbreviation: CRISPR, clustered regularly interspaced short palindromic repeats.

### 4.1 Viral Vectors

rAAV represents the most clinically advanced platform for in vivo genome editing; a total of 238 rAAV‐based gene therapy clinical trials have been registered on ClinicalTrials.gov as of 2024 [161]. Approved therapies such as Zolgensma for spinal muscular atrophy (SMA) [162] and Upstaza for aromatic l‐amino‐acid decarboxylase deficiency [163] have demonstrated durable benefit, while investigational editor programs highlight its versatility. Notably, rAAV has enabled CEP290 disruption in EDIT‐101 trial [164] and HIV proviral excision in EBT‐101 study [165]. Additionally, the AAV8 vector leads to sustained reduction of circulating PCSK9 and LDL cholesterol for over 3 years [166]. These examples illustrate the capacity of viral vectors to reach therapeutically meaningful efficacy.

At the same time, three fundamental barriers limit broader translation: rAAV's ∼4.7 kb packaging capacity limits restricts the size and complexity of genome‐editing cargos [167, 168]. Immunogenicity and systemic dose‐related toxicities restrict eligibility and complicate repeat dosing [169, 170]. For clinical application, rAAVs demand industrial‐scale and cost‐effective production methods. These constraints underscore the need for continued engineering of vector tropism, genome architecture, and production platforms.

The following subsections discuss how each major viral system—AAV, LVs, and adenoviral/Ad–AAV hybrids—has been adapted to address these challenges. AAV remains the benchmark for in vivo applications, LVs dominate ex vivo therapies, and adenoviral systems offer unmatched capacity for large editors. Together, they illustrate both the achievements and ongoing limitations of viral vector‐based genome editing.

#### 4.1.1 Adeno‑Associated Virus

rAAV remains the most widely applied platform for in vivo genome editing, building on its broad tissue tropism, efficient nuclear entry, and decades of clinical use. Editor‐based applications have highlighted its reliability while revealing persistent limitations in cargo capacity, immune barriers, and manufacturing burden, issues that motivate extensive engineering efforts.

Single‐vector AAVs enable efficient, one‐time delivery of therapeutic genes or genome‐editing tools, with capsid re‐engineering and tropism control being central to enhancing transduction efficiency and tissue specificity [171, 172, 173]. For example, a liver‐tropic AAV3 achieved FIX activity within or near normal range at a low vector dose of 5 × 10^11^ vector genomes/kg [174]. Optimization of compact Cas9 orthologs or domain‐deleted editors enables compatibility with single‐vector delivery, though these often narrow the editing window or necessitate gRNA redesign [175, 176, 177]. Incorporating regulatory modules enhances precision, and microRNA‐122 target sites in AAV9–Tnnt2 restrict hepatic expression [178], while local AAV8 delivery achieves confined SaCas9 expression in glioblastoma models [179]. CRISPR activation at AAV ITRs boosts transgene expression [180], and host‐factor screening implicates Golgi and trafficking pathways as key regulators of yield and transduction efficiency [181, 182]. Together, these studies point to promoter design, receptor targeting, and producer cell engineering as levers to maximize potency.

Genome redesign strategies aim to accelerate transgene or editor activity. Self‐complementary AAV (scAAV), generated by deleting the terminal resolution site in one inverted terminal repeat, circumvents the rate‐limiting step of second‐strand synthesis and enables faster transgene expression [183]. In a Duchenne muscular dystrophy (DMD) model, a dual‐vector delivery, in which Cas9 was delivered via single‐stranded AAV and sgRNA via scAAV, restored dystrophin expression at lower scAAV doses, indicating that accelerated sgRNA expression can be critical for phenotypic rescue [119].

Dual‐AAV systems expand vector capacity for large editors by using split inteins or dimerization domains to reconstitute BEs or PEs in vivo. However, the editing efficiency of reassembly and functional recovery varies widely, ranging from ∼9 to 60% across liver, heart, retina, skeletal muscle, and CNS, depending on the design [89]. Therefore, rational selection of split sites and reconstitution domains is critical, as suboptimal architectures can markedly reduce expression or editing fidelity [184]. Notably, in phenylketonuria mice, intein‐split cytidine BEs corrected up to ∼21% on‐target editing, leading to the reversal of the disease phenotype [122].

Beyond molecular design, the practical implementation of rAAV systems is further constrained by manufacturing and safety challenges [185, 186]. rAAV production remains limited by producer‐cell capacity and cassette architecture, and dual‐vector assembly adds substantial complexity to large‐scale manufacturing. Although single‐AAV systems offer superior yield, dual‐vector systems add complexity to large‐scale production [187, 188]. Immunogenicity also limits readministration, as pre‐existing antibodies can neutralize efficacy [189]. Vector integration or chromosomal rearrangements remain risks, particularly with incomplete packaging [190, 191]. Mitigation includes degradable editor designs, microRNA‐regulated promoters, and comprehensive biodistribution and integration profiling. Advances in AAV serotype engineering, genome design, and scalable manufacturing are addressing key safety and efficiency barriers. Innovations such as scAAV, dual‐vector systems, and capsid evolution enhance expression and tissue targeting. Integrating these developments with immune control and genomic‐stability strategies is crucial for achieving safe, efficient, and durable clinical translation.

#### 4.1.2 Lentiviral Vectors

LVs are central to ex vivo genome editing, combining a ∼10 kb payload capacity, efficient transduction of dividing and nondividing cells, and scalable manufacturing [192, 193]. Their design balances the durability of genomic integration with the safety of transient expression. Integrating LVs (ILVs) provide stable long‐term expression, exemplified by successful correction of immunodeficiencies [194]. However, continuous nuclease activity increases off‐target and insertional risks even with self‐inactivating long terminal repeats [195]. Integrase‐deficient LVs (IDLVs) mitigate these hazards by generating episomal cDNA that supports transient yet efficient editing, though limited persistence and rare host‐mediated integration remain challenges [196, 197].

Envelope pseudotyping further refines tropism and safety. Replacement of the native HIV‐1 envelope with vesicular stomatitis virus glycoprotein confers broad tropism and particle stability, while envelopes from baboon endogenous or measles viruses enhance CD34^+^ HSC targeting and reduce off‐target transduction [198, 199]. “Nanoblade” systems, fusing murine leukemia virus (MLV) Gag to Cas9, yield 30–40% editing in CD34^+^ cells (HSPCs) and ∼40% in T cells, with antibody fragment display targeting CD4^+^ or CD8^+^ subsets [200]. Manufacturing refinements further strengthen safety. Excluding SV40 T‐antigen sequences from 293T producer lines preserves high‐titer while preventing oncogenic carryover [201], and high‐resolution mapping methods such as CReVIS‐seq enable precise assessment of vector integration [202]. The adaptation of LVs has expanded their relevance to CRISPR‐based editing. IDLV‐based Cas9 delivery enables one‐step correction of sickle‐cell mutation with up to 10% protein‐level correction in patient‐derived HSPCs [203], and multiplexed gRNA backbones permit simultaneous gene disruption. Nonetheless, prolonged effector expression can reduce vector yield and cell viability, underscoring the need for precise control of nuclease dosage and exposure [203].

Preclinical studies have validated LV efficacy across diverse disease contexts. In hemoglobinopathies, γ‐globin or RPS19‐encoding vectors restore hematopoiesis and correct disease phenotypes [204]. LVs are equally central to T‐cell and iPSC engineering. In CAR‐T therapy, LV‐mediated CRISPR–CAR19 editing induced durable remission in pediatric B‐ALL [139]. In iPSC systems, LVs carrying safety modules, such as suicide switches or AAVS1‐anchored Cas9 cassettes, enable controlled differentiation and mitigate tumorigenic risks [205, 206, 207]. These strategies showcase the versatility of LVs in combining editing and safety elements within a single platform.

Recent engineering advances have reduced major genotoxic risks in LV‐based editors, including insertional mutagenesis and off‐target nuclease activity. Transient IDLVs and VLP‐RNP delivery shorten nuclease exposure and reduce chromosomal rearrangements [208, 209], while high‐fidelity Cas9 variants and microRNA‐regulated payloads restrict activity to target cells [210, 211, 212]. Hybrid architectures combining IDLVs with LNP or VLP systems balance precision with scalability for safer genome editing.

In summary, integrating and non‐ILV systems offer complementary strengths for ex vivo genome editing. Envelope pseudotyping enhances cell‐type specificity, and transient nuclease expression minimizes genotoxicity. With their large payload capacity, scalable production, and established clinical record, LVs remain core platforms driving safe and durable genome‐editing therapies [213].

#### 4.1.3 Adenovirus and Hybrid Ad/AAV Systems

Distinct from AAV or lentivirus systems, helper‐dependent adenoviral vectors (HDAd, or “gutless,” Ad) retain only inverted terminal repeats and packaging signals, allowing payloads of up to ∼36 kb [214, 215]. This vast capacity enables single‐vector delivery of full‐length Cas nucleases, multiple gRNAs, and donor templates for complex editing [216]. The episomal Ad genome supports high expression in both dividing and quiescent cells without insertional mutagenesis risk.

Early Ad vectors triggered severe immune reactions due to residual viral genes, exemplified by the 1999 OTC deficiency trial [217]. Fully deleted HDAd systems were developed, removing all viral open reading frames to minimize antigenicity and cytotoxic T cell activation [214, 215]. In NHPs, systemic administration below complement‐activating thresholds achieved years‐long hepatic transgene expression without inflammation [218, 219]. Cre‐lox excision and inducible helper control further minimized contamination (<0.1%) and improved batch consistency [220, 221].

HDAd's large payload enables multicomponent editing systems such as HDAd5/35++, which can deliver up to 30 kb of editors to hematopoietic stem cells and restore HbF expression in vivo [222]. Continued capsid engineering, including fiber swaps, polymer coatings, and bispecific adaptors, has reduced pre‐existing immunity and complement activation while improving tissue targeting [223]. Collectively, these refinements have transformed HDAd from a proinflammatory vector into a clinically viable, large‐capacity delivery vehicle.

Adenovirus‐based platforms have become indispensable in disease modeling and functional genomics. Ad5 vectors effectively deliver CRISPR/Cas9 to disrupt target genes such as MYOC in glaucoma and tumor suppressors (Rb1, Tp53) in lung cancer, enabling faithful disease recapitulation in vivo [224, 225]. Genome‐wide CRISPR screens have further identified host factors such as *FANCA*, *SETDB1*, and *MORC3* that suppress transgene expression, suggesting routes to improve adenoviral and AAV vector performance [226].

High‐capacity adenoviral vectors (HCAdVs) enable complex genome editing. In HBV models, HCAdV with Cas9 and multiple gRNAs reduced viral antigens and cccDNA [130]. In hemophilia B, a tetracycline‐regulated CRISPR/Cas9–AAV system achieved 5.52% *F9* correction, while HDAd5/35++ supported stable *AAVS1* integration in HSCs [131, 134]. Hybrid Ad/AAV vectors combine adenoviral efficiency with AAV tolerance: they match AAV6 in sickle‐cell models and correct *COL7A1* mutations, restoring collagen VII in epidermolysis bullosa [136, 227]. These designs balance potency and safety for large‐payload genome editing.

In summary, adenoviral vectors have evolved from early immunotoxic prototypes into highly adaptable gene‐delivery platforms. Modern HDAd and hybrid Ad/AAV platforms combine large payload capacity, episomal stability, and improved safety, positioning them as leading options for complex or multicomponent genome‐editing applications.

### 4.2 Physical Delivery Approaches

Physical delivery methods remain indispensable for introducing genome‐editing reagents when chemical or viral vectors are unsuitable. Current strategies include ex vivo electroporation, nucleofection, microinjection, and hydrodynamic limb or portal‐vein injection. A shared advantage among these approaches is the direct cytosolic transfer of RNP complexes, mRNA, or plasmid DNA, which minimizes prolonged nuclease expression and off‐target activity. Nonetheless, each technique balances distinct trade‐offs between efficiency, safety, and scalability [228, 229].

Electroporation remains the most established method, relying on short electric pulses to transiently permeabilize the plasma membrane. It enables efficient editing in zygotes, primary cells, and pluripotent stem cells ex vivo, as rapid membrane resealing preserves viability and supports delivery of diverse editor modalities, including HDR donors, BEs, PEs, and homology‐independent targeted integration systems. This versatility has made electroporation a cornerstone of adoptive cell therapies, where autologous cells are edited and reinfused. Nonetheless, membrane disruption restricts in vivo use due to local tissue injury risk, confining application to cells tolerant of physical stress [230, 231, 232, 233]. Efforts to scale electroporation have yielded flow‐through and hydroporation systems, which increase CAR‐T yields by ∼1.7–2‐fold versus conventional systems and improve viability, proliferation, and effector function [234, 235, 236]. A flow‐based platform enables continuous processing of up to 256 million T cells per minute, achieving >95% mRNA transfection with minimal viability loss [237]. These advances support high‐throughput, clinically scalable editing while highlighting the need for automation and closed‐system control.

Nucleofection, which targets both plasma and nuclear membranes, operates via localized electric fields and offers editing frequencies comparable to conventional electroporation while accommodating large RNPs and donor templates. However, it has not consistently improved safety or efficiency, and higher voltages can compromise viability, limiting use to ex vivo workflows that require precise integration or HDR [238, 239]. To overcome throughput and sensitivity constraints, microfluidic and mechanoporation platforms have emerged. Viscoelastic mechanoporation delivered CRISPR–Cas9 at rates exceeding 250 million cells per minute via hydrodynamic stress without electrode contact [240]. Adoption remains limited by specialized media, precise flow control, and device fabrication costs.

Nanoscale and photothermal approaches offer minimally invasive, spatially controlled delivery. Nanostraw‐mediated electroporation enabled localized cargo transfer into primary immune cells with reduced membrane disruption, supporting uptake of mRNA, proteins, and RNPs [241]. Hollow nanoneedle arrays deliver Cas9 RNPs directly to nuclei in dendritic cells, bypassing cytoplasmic barriers [242]. Photothermal transfection using iron oxide‐embedded fibers transiently permeabilizes T cell membranes, supporting efficient editing and clonal expansion [243]. These technologies enhance precision but currently rely on custom nanofabrication and specialized optics, constraining scale‐up.

Direct in vivo physical delivery shows proof‐of‐concept but faces practical barriers. Intramuscular electroporation in turquoise killifish yielded months‐long expression from a single pulse [244]. Microinjection of CRISPR reagents into zebrafish embryos remains widely used for developmental mutagenesis yet is labor‐intensive and low‐throughput [143]. Hydrodynamic delivery via limb or portal vein enables transient organ‐wide delivery through rapid infusion of large volumes but is difficult to translate due to endothelial stress, inflammation, and pressure‐scaling limits in humans [245, 246].

In summary, physical delivery defines a trade‐off in which editing efficiency and transient nuclease exposure must be balanced against cell viability, throughput, and translational feasibility. Continued refinements, including pulse optimization, flow‐based automation, and tissue‐protective strategies, have improved yield and reproducibility. Realizing therapeutic impact will require integrating these advances into scalable, automated, and tissue‐compatible delivery systems.

### 4.3 Lipid‑Based Nanoparticles

Ionizable LNPs have emerged as the foremost nonviral vectors for nucleic acid and genome‐editing therapeutics, owing to their modular chemistry, biocompatibility, and clinical scalability. Composed of an ionizable lipid, cholesterol, a helper phospholipid (e.g., DSPC), and a PEG–lipid, LNPs achieve a delicate balance between colloidal stability and endosomal escape efficiency [14, 247]. Ionizable headgroups remain neutral at physiological pH to minimize toxicity but protonate under acidic conditions to facilitate endosomal membrane and cargo release [83, 248]. Clinically validated lipids such as DLin–MC3–DMA, SM‐102 (Moderna), and ALC‐0315 (Pfizer/BioNTech) illustrate how amine placement, tail branching, and p*K*_a_ tuning govern encapsulation and delivery efficiency [249].

These design principles enable the delivery of both mRNA and RNPs complexes. Rapid microfluidic mixing under acidic conditions drives electrostatic complexation and formation of protective cores; coencapsulation of sgRNA or Cas9 RNP supports precise and transient editing while limiting off‐target activity [142]. LNPs first proved clinical effectiveness in siRNA therapeutics (patisiran), establishing the regulatory and manufacturing frameworks now adapted for CRISPR applications [250, 251]. The rapid success of mRNA vaccines (BNT162b2, mRNA‐1273) further demonstrated scalability and safety, paving the way for CRISPR therapeutics [252]. In the landmark NTLA‐2001 trial, codelivered Cas9 mRNA and TTR‐targeting sgRNA achieved an average 87% reduction of serum transthyretin at a single 0.3 mg kg^−1^ dose, followed by additional successes such as NTLA‐2002 (KLKB1) and VERVE‐101 (PCSK9 BE) [9, 15, 253].

Tissue‐specific delivery is primarily dictated by the interplay among helper lipids, sterols, PEG–lipids, and the serum‐derived protein corona that forms postinjection [254, 255, 256, 257]. Neutral DSPC promotes ApoE adsorption and LDL receptor‐mediated hepatic uptake, explaining the innate liver tropism of early LNPs [258]. Substituting cationic or anionic helper lipids reshapes the corona to favor vitronectin or Tim4 binding, redirecting localization to lung or immune cells [76]. “SORT” LNPs using these principles achieved up to 60–80% gene knockdown in lung tissue [259]. These findings underscore how rational formulation engineering can guide LNP tropism and minimize off‐target risks.

The ability to encapsulate RNPs remains central to CRISPR delivery [260]. Recent formulations combining optimized helper lipids with cell‐penetrating peptides (CPPs) have achieved over 300‐fold higher gene‐editing efficiency than unformulated RNPs [261]. Codelivery of Cas9 protein and sgRNA has enabled genome correction in hepatocytes, hematopoietic stem cells, and fetal models [150, 262, 263], while BEs delivery and targeted moieties (e.g., phenylboronic acid for sialic acid‐rich cancer cells) further enhance precision [264]. Despite these advances, key challenges remain, including maintaining RNP stability, minimizing immunogenicity, and improving endosomal escape without triggering strong innate responses.

High‐throughput synthesis and biological screening of lipid variants has revealed that subtle modifications, such as tail length, branched headgroup motifs, or hydroxyl group density, can profoundly affect delivery efficiency and cytotoxicity [146, 265, 266]. These insights now inform the rational design of next‐generation LNPs optimized for mRNA, siRNA, and RNP payloads. Nonetheless, challenges persist: combinatorial synthesis remains resource‐intensive, preclinical models often fail to predict human responses, and long‐term safety, immune activation, and repeated dosing tolerance are insufficiently characterized. Moreover, some of the most effective lipids require complex chemistries not yet suited for large‐scale clinical manufacturing.

Overall, iterative refinement has transformed LNPs from experimental carriers into cornerstone clinical vectors for nucleic acid and genome‐editing therapeutics. Their capacity to encapsulate and protect labile payloads underlies a growing portfolio of United States Food and Drug Administration (US FDA)‐approved and investigational products [111, 250, 267]. Clinical trials such as NTLA‐2001 validate their translational potential, and continued advances in lipid chemistry and formulation design are expected to drive the next generation of genome editing toward broader therapeutic reach.

#### 4.3.1 Engineering LNP Tropism and Endosomal Escape

LNP tropism is determined not by the nucleic acid payload but by the physicochemical interplay of four key components: helper lipids, sterols, PEG–lipids, and the serum‐derived protein corona that forms immediately after systemic administration. The dynamic balance among these components dictates whether LNPs undergo hepatic clearance or reach extra‐hepatic targets, thus shaping both therapeutic index and off‐target risk [247, 268].

Helper lipids, typically 5–15 mol% of total composition, play active roles beyond structural stabilization. Neutral phosphatidylcholines such as DSPC favor ApoE adsorption and LDL receptor‐mediated hepatic uptake, explaining the strong liver tropism of early MC3‐ or ALC‐0315‐based systems. Substituting DSPC with permanently cationic or anionic analogs alters corona composition: cationic helper lipids promote vitronectin binding and uptake by pulmonary endothelial cells, while anionic phosphatidylserines engage scavenger receptors such as Tim4, redirecting distribution toward immune and endothelial compartments. “SORT” LNPs based on such lipid polarity tuning have achieved up to >70% editing in lung tissue with minimal hepatic transfection [76, 254, 269].

Sterol composition serves as a secondary determinant of organotropism. Standard cholesterol maintains endosomal stability and supports hepatic expression, whereas plant‐derived sterols and oxysterols disrupt lipid packing and modify the protein corona, thereby enabling preferential accumulation in the spleen or bone marrow [270, 271, 272]. Similar modulation occurs with hydrophobic conjugates: cholesterol–siRNA complexes associate with LDL particles and localize to hepatocytes, while less hydrophobic analogs bind HDL and redirect to kidney, heart, or lung tissues [273, 274].

PEG–lipids provide a third, time‐sensitive layer of control. Anchored PEG chains enhance colloidal stability and reduce opsonization but gradually detach in circulation. Rapidly shedding, short anchors favor ApoE adsorption and hepatic uptake, whereas long or branched anchors extend circulation time and promote alternative protein adsorption. Incremental increases in PEG–lipid content (1–3%) can substantially reduce liver accumulation and enhance delivery to ocular, splenic, or pulmonary tissues [275, 276, 277].

Ultimately, the serum protein corona crystallizes LNP fate. ApoE dominates canonical formulations, but its replacement or suppression through dense PEG layers or modified surface chemistries that favor vitronectin, albumin, or complement factors enables extra‐hepatic targeting without exogenous ligands. Because the corona evolves dynamically through PEG shedding and lipid exchange, host‐ or disease‐specific serum compositions may further bias LNP biodistribution, offering opportunities for pathology‐selective targeting [74, 278, 279, 280].

These parameters, helper lipid charge, sterol hydrophobicity, PEG–lipid dynamics, and corona composition, are interdependent and collectively determine organ selectivity [281]. For example, lung‐specific targeting arises from cationic helper lipids and slow‐shedding PEG anchors [281], whereas spleen‐directed formulations employ oxidized sterols and intermediate PEG kinetics [282]. High‐throughput in vivo barcoding now enables systematic mapping of this multidimensional design space, challenging the traditional assumption that hepatotropism is an intrinsic property of LNPs [283].

Beyond tropism, chemical engineering of ionizable lipids has substantially improved intracellular delivery. LNP uptake involves heparan sulfate proteoglycans and vacuolar ATPase activity, which coordinate endosomal maturation [284]. Rational headgroups and tail engineering, adjusting p*K*_a_, linker flexibility, and hydrophobic packing, has achieved an increase in reporter expression. Branched tails enhance endosomal penetration, while combinatorial chemistries such as Passerini‐derived biodegradable libraries allow iterative optimization of charge distribution and degradation kinetics [266, 285].

Fine‐tuning the membrane‐disruptive capacity of ionizable lipids is central to efficient endosomal escape while minimizing immune activation. Small changes in hydrophobic tail geometry or linker composition can suppress type I interferon responses, prolong cytosolic retention, and reduce innate immune activation. Rationally optimized LNPs have achieved up to 70% Cas9 mRNA and sgRNA‐mediated knockout efficiency [285], while disulfide‐bridged linkers introduce glutathione‐responsiveness, triggering redox‐mediated endosomal release and producing up to 87‐fold higher hepatic mRNA levels than clinical benchmarks [286]. Multitailed ionizable phospholipids further promote hexagonal phase transitions in acidic endosomes, enhancing cytosolic release without relying on proton‐sponge effects [287]. Formulations with hydroxycholesterols or stable cholesterol domains enhance late endosomal trafficking and DNA protection, achieving gene editing efficiencies up to 39% with Cas9/sgRNA complexes [288].

Innovative fusion‐based nanoplatforms have also advanced intracellular trafficking. Hybrisome‐LNPs incorporating cell‐membrane proteins show up to 15‐fold higher cellular uptake and eightfold enhancement in mRNA translation [289]. pH‐responsive cationic/ionizable hybrids enable electrostatic fusion within endosomes [290, 291], while redox‐responsive systems deliver nearly 90% of Cas9 RNPs into cytoplasm and nucleus [292].

Despite these advances, several translational hurdles remain. LNP‐mediated CRISPR delivery still faces variability in sgRNA release, endosomal kinetics, and immune sensing. Optimization of guide RNA design and modulation of innate immune sensing have facilitated prime editing efficiencies exceeding 50% in vitro, with rapid onset posttransfection [148, 293]. Codelivery strategies incorporating redox‐triggered platforms further improve the efficiency and specificity of CRISPR plasmid or RNP delivery across diverse disease models [292, 294]. Single‐dose delivery of nuclease‐null Cas9 in LNPs has also achieved durable transcriptional activation [295]. Dose‐dependent toxicity from cationic lipids underscores the need for quantitative safety profiling [296]. Moreover, rodent models inadequately capture human serum diversity and organ physiology, which strongly influence corona formation and biodistribution.

Advances in lipid composition tuning, PEG–lipid dynamics, and serum‐corona modulation have transformed LNP tropism from a fixed property into a programmable feature. Optimized ionizable‐lipid chemistry, together with emerging fusion or redox‐responsive designs, enables efficient, tissue‐selective genome editing with improved endosomal escape, reduced immune activation, and strong translational potential.

### 4.4 Polymeric and Hybrid Nanoparticles

Polymeric and hybrid nanoparticles represent a versatile class of nonviral carriers for genome editing, condensing nucleic acids through electrostatic interactions and facilitating endosomal escape via the proton sponge effect. Their tunable chemical structures allow fine control of delivery efficiency, biodegradability, and cytotoxicity [290, 291, 297, 298]. Representative systems include PEI, PBAEs, highly branched PBAEs (HPAEs), poly(amido amine) (PAMAM) dendrimers, star‐shaped cationic polymers (SCKP), and polymer–lipid hybrids [299, 300, 301].

PEI remains a benchmark polymer owing to its dense amine network and strong transfection ability, achieving high editing efficiency in vitro with 25 kDa branched PEI [302]. However, its nondegradable backbone and high charge density lead to dose‐dependent cytotoxicity and inflammation. Chemical modifications such as PEGylation reduce cytotoxicity while largely preserving delivery potency [303, 304]. PBAEs overcome PEI's limitations via hydrolytically degradable ester linkages, yielding safer degradation profiles [305]. High‐throughput synthesis produces variants with high editing efficacy and low lactate dehydrogenase release. Nebulized PBAE nanoparticles efficiently deliver mRNA and sgRNA to mouse lungs with minimal toxicity, and repeated dosing in large animals maintains normal serum biochemistry, confirming favorable pharmacokinetics and limited organ stress [306]. Architectural refinements have further enhanced the PBAE platform. Guanidinium‐functionalized HPAEs combine dense amine functionality with controlled degradability, achieving superior editing efficiency over linear PBAEs while maintaining cell viability [307]. Similarly, star‐shaped SCKPs with cleavable backbones achieve PEI‐comparable transfection with threefold lower cytotoxicity [308], demonstrating how architectural tuning balances charge density and biocompatibility. Similarly, engineering disulfide bonds into PAMAM dendrimers improves biodegradability and halves cytotoxicity while maintaining delivery efficiency [300]. Hybrid polymer–lipid systems balance delivery efficiency and safety by coformulating PBAEs with helper lipids, which masks surface charge, improves serum stability, and extends circulation [309]. For instance, PEG‐b‐PLGA lipomers reduce hepatic accumulation and enable efficient single‐dose editing in difficult targets like the heart and vasculature [152]. Lipomer platforms like 7C1 also enable siRNA delivery to endothelial cells with minimal immune activation, demonstrating that lipid components act as both structural stabilizers and biological camouflage [310, 311].

Comparative analyses across polymeric systems reveal several design rules. First, biodegradability inversely correlates with toxicity: nondegradable PEI and PAMAM induce stronger inflammatory responses, whereas ester‐linked PBAEs, HPAEs, and SCKPs degrade into harmless by‐products [312, 313, 314]. Second, editing efficiency depends on charge density and endosomal escape capability; while PEI remains potent, guanidinium–PEI, hydrophobic–PEI, and PBAEs approach similar performance with greater safety [315, 316, 317]. Third, polymer–lipid hybrids offer synergy: lipid coatings retain cationic core activity for endosomal escape yet present a neutral exterior, lowering immune activation and off‐target distribution [318].

Building on these foundations, degradable polymeric carriers now serve as key CRISPR/Cas9 platforms. For instance, Cas9 conjugated to PAMAM via disulfide linkers enables near‐complete GFP knockout and 20% VEGF disruption in vitro [319]. Oxidative stress‐suppressing PBAEs yield 10–100% higher editing across cell types [158], cross‐linked PBAEs achieve ∼8.5% editing at oncogene loci [320], and chitosan‐quaternized derivatives show >5‐fold improvement over PEI [321]. Nevertheless, excessive surface charge (high ζ‐potentials) can still provoke cytotoxicity, underscoring the need to optimize polymer branching and chain length [322].

Advanced systems further expand functionality. Stimulus‐responsive polymers enable spatially controlled editing through NIR‐triggered, ATP‐responsive, or serum‐stable platforms, though repeated dosing requires safety evaluation [151, 323, 324]. Hybrid nanoparticles integrating polymers with lipids or biological membranes enhance delivery capability. For instance, phenylboronic branched polymer–liposome assemblies achieve ∼50% STAT3 knockout in hypoxic glioblastoma [157]. PEI–lipid hybrids improve pulmonary mRNA delivery 300‐fold over commercial reagents [159]; and exosome–liposome hybrids with aptamer decoration achieve 42–55% editing with improved tumor selectivity [289, 325]. Among membrane‐based strategies, stem‐cell‐membrane coatings have been shown to enhance tumor accumulation [326]. Applying this concept to HBV therapy, Wu et al. recently developed surfactant‐based nanoparticles cloaked with hepatocyte membranes. This biomimetic system exploits homotypic targeting for liver‐specific delivery, while the membrane coating reduces the intrinsic cytotoxicity of the cationic polymer. Notably, it redirects cellular uptake pathway from clathrin‐mediated endocytosis, typically associated with lysosomal degradation, to caveolin‐mediated pathways that bypass lysosomes, thereby improving DNA intracellular stability and facilitating nuclear delivery. As the result, the system achieved a transfection efficiency of 54.81% and reduced HBV cccDNA by 96.47% in vitro [327]. Additionally, acetalated oligosaccharide–PEI hybrids successfully delivered CRISPR cargo to cervical tumors with minimal toxicity [328]. While promising, these systems need further assessment of biodistribution and immunogenicity.

In conclusion, polymeric and hybrid nanoparticles have evolved from simple PEI or PAMAM scaffolds into sophisticated, multifunctional delivery systems. Advances in biodegradable polymers, dendrimers, and hybrid materials have markedly improved editing specificity, biocompatibility, and manufacturing scalability. Looking forward, modular designs integrating controlled biodegradation, efficient endosomal escape, and tissue‐specific targeting represent the most promising direction for next‐generation CRISPR delivery systems. Their clinical translation will require continued material optimization and rigorous preclinical validation, particularly in large animal models, to fully realize the therapeutic potential of genome editing.

## 5 Emerging Delivery Systems

**FIGURE 4:**
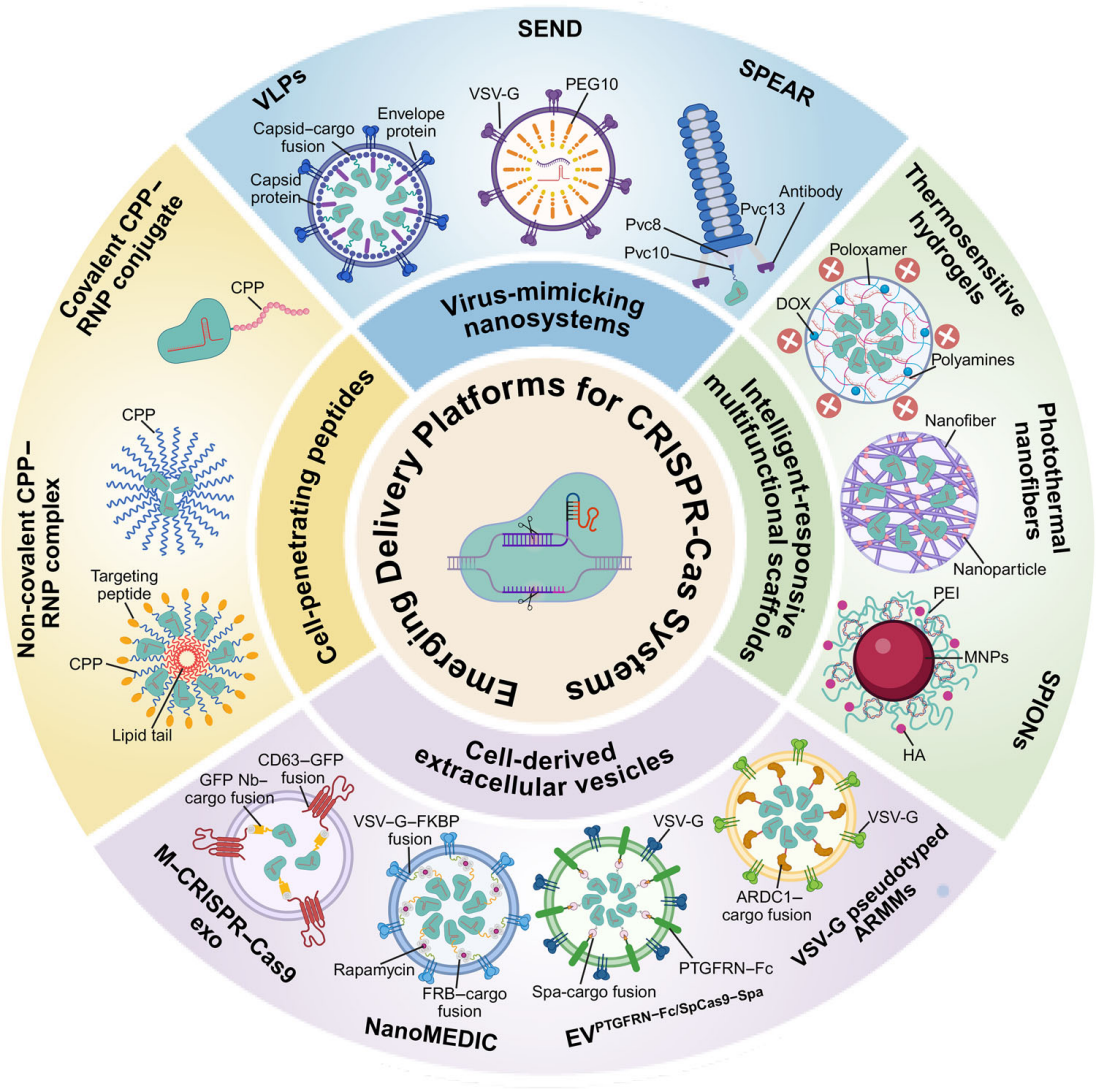
Structural and functional diversity of emerging delivery platforms for CRISPR–Cas systems. Schematic illustration of four representative classes of emerging delivery platforms, defined by their distinct design principles and structural features: (1) *Virus‐mimicking nanosystems* (e.g., VLPs, SEND, SPEAR): synthetically engineered or bio‐inspired nanostructures that emulate viral mechanisms of cellular entry and intracellular trafficking, thereby enhancing the delivery efficiency of CRISPR cargoes. (2) *Cell‐derived extracellular vesicles* (e.g., M‐CRISPR–Cas9 exo, NanoMEDIC): biocompatible, naturally derived nanovesicles functionalized with targeting ligands or protein effectors to achieve precise delivery of genome‐editing components. (3) *Cell‐penetrating peptides (CPPs)*: modular peptide‐based carriers that facilitate direct cytosolic translocation of RNPs through non‐covalent complexation or stie‐specific covalent conjugation. (4) *Stimuli‐responsive multifunctional scaffolds* (e.g., thermosensitive hydrogels, photothermal nanofibers): biomaterial‐based systems tailored for spatiotemporally controlled release of RNPs, mRNA, or plasmid DNA in response to physiological cues or externally applied triggers. ARMMs, arrestin domain‐containing protein 1‐mediated microvesicles; CPP, cell‐penetrating peptides; CRISPR, clustered regularly interspaced short palindromic repeats; EV, cell‐derived extracellular vesicles; M‐CRISPR–Cas9 exo: modified CRISPR–Cas9 exosomes; NanoMEDIC, nanomembrane‐derived extracellular vesicle for delivery of macromolecular cargo; SEND, selective endogenous encapsulation for cellular delivery; SPEAR, synergistic programmable enhancer‐antigen receptor; SPIONs, superparamagnetic iron oxide nanoparticles; VLPs, virus‐like particles. (Figure was created with BioRender.com.)

Efforts to combine the high efficiency of viral vectors with the safety and scalability of nonviral platforms have given rise to a new generation of delivery technologies. These vectors retain the efficient cellular uptake and endosomal escape typical of viruses while eliminating genome integration and minimizing immunogenicity, two major limitations of conventional viral therapies. A comparative profile of these emerging nonviral CRISPR delivery platforms, detailing their composition, cargo, target genes, and editing efficiencies, is provided in Table 2, offering critical insights for selecting and optimizing future delivery strategies. Recent innovations in VLPs represent an evolution that entirely omits viral genomes. Lentiviral‐derived VLPs have been optimized to efficiently package and deliver RNPs, achieving therapeutic levels of genome editing in multiple tissues with reduced off‐target activity [109, 110, 329]. Transitioning to mRNA cargo further expands their modularity and allows multiplexed editing with reduced innate immune activation [330, 331]. Fully synthetic particles such as SEND, assembled from human‐derived components, further extend delivery capacity without persistent expression [332, 333] (Figure 4).

**TABLE 2:** Summary of emerging delivery platforms for CRISPR–Cas systems.

Delivery system	Composition	CRISPR/Cas9 cargo	Target gene (s)	Editing efficiency	References
Virus‐mimicking nanosystems					
VLPs (Cas9P LV)	VSV‐G, Gag‐Pol, and Gag‐cargo (a fusion construct of Cas9, an HIV‐1 protease cleavage site, and the PH domain, inserted at the N‐terminus of Gag)	Cas9 protein	CCR5	∼15% (CCR5 knockout in sgRNA‐expressing primary CD4^+^ T cells)	[334]
VLPs	VSV‐G or HIV‐1 envelope glycoprotein, Gag‐Pol, and Gag‐cargo (Cas9 fused to the C‐terminus of Gag via an HIV‐1 protease‐cleavable linker); optional lentiviral transfer plasmid encoding sgRNA and/or a transgene (e.g., CAR, mNeonGreen)	RNP	B2M, TRAC	∼23.9% (B2M/TRAC double knockout in CD4^+^ T cells)	[335]
eVLPs (v4 BE–eVLPs)	VSV‐G or FuG‐B2 envelope glycoprotein, MMLV gag–pro–pol, MMLV gag‐3xNES‐cargo (ABE8e, a highly active adenine base editor, fused to the C‐terminus of MMLV gag via an optimized MMLV protease‐cleavable linker) and sgRNA	Base editor RNP	Pcsk9	∼63% (Pcsk9 knockdown in the mouse liver)	[110]
eVLPs (v3 PE‐eVLPs)	VSV‐G, MMLV Gag‐Pol, Gag‐P3‐Pol, P4‐PE, and engineered pegRNAs	Prime editor RNP	Dnmt1	∼47% (mouse brain, GFP^+^ nuclei)	[336]
VLPs (RIDE)	VSV‐G or hyRV‐G, MS2‐coat‐gagpol, GagPol, Cas9, and MS2‐stem loop‐gRNA	RNP	Vegfa, AAVS1, Htt	Highly efficient editing (at multiple endogenous loci across diverse cell lines); average 1.45% (in the striatum samples of mice)	[109]
SEND	VSV‐G or SYNA, PEG10, cargo mRNA containing the first 500 bp of the 3′ UTR	Cas9 mRNA and sgRNA	Kras, VEGFA	∼30% (Kras knockout in mouse N2a cells); ∼40% (VEGFA knockout in HEK293FT cells)	[332]
CISs (PVC)	PVC structural complex (pvc1‐16), Ad5‐knob‐retargeted tail fibre (Pvc13), cargo proteins loaded via N‐terminal packaging domain	Cas9 protein	Unspecified	Produced on‐target indels (in HEK293FT cells with a guide RNA)	[337]
CISs (SPEAR)	PVC structural complex (pvc1‐16), a modular tail fibre (Pvc13) functionalized with conjugated antibodies, and a spike complex (Pvc8/Pvc10) for cargo loading	RNP	VEGFA	∼5–7% (VEGFA knockout in HEK293FT cells)	[338]
**Cell‐derived extracellular vesicles**					
M‐CRISPR–Cas9 exosome	HEK293T cells‐derived exosomes, CD63–GFP, SpCas9–GFP nanobody and sgRNA	RNP	A stop cassette	Clear appearance of DsRed fluorescent signal in A549^stop‐DsRed^ cells, indicating successful deletion of the stop cassette and frameshift correction	[339]
NanoMEDIC	HEK293T cells‐derived exosomes, VSV‐G–FKBP12 or a combination of VSV‐G and FKBP12–Gag, FRB–SpCas9, AP21967, and sgRNA	RNP	DMD exon 45	Over to 92% exon skipping (in DMD patient iPSC‐derived skeletal muscle cells)	[340]
EV^PTGFRN−Fc/SpCas9−Spa^	HEK293T cells‐derived EVs, VSV‐G, PTGFRN‐Fc, SpCas9−Spa, and sgRNA	RNP	UL8, UL29	∼21.71% (UL29) and ∼3.25% (UL8) indel frequency (in HSV1‐infected Hela cells)	[341]
ARMMs	HEK293T cells‐derived microvesicles, ARRDC1, WW–Cas9 fusion proteins, and sgRNA	RNP	GFP	∼13.4% (in U2OS cells with stable expression of GFP protein)	[342]
VSV‐G pseudotyped ARMMs	HEK293T cells‐derived microvesicles, VSV‐G, ARRDC1–Cas9 and sgRNA	RNP	GFP, APP	∼40% (in U2OS‐gfp cells); ∼90% (in human neural progenitor ReNcell CX cells); ∼40% (in human iPSC‐derived neurons)	[343]
Gesicles	HEK293FT cells‐derived EVs, VSV‐G, CherryPicker Red with DmrA domain, Cas9 (fused with DmrC domain), A/C heterodimerizer and sgRNA	RNP	HIV LTR	∼8% mutation efficiency (in HIV‐NanoLuc CHME‐5 cells)	[344]
Gectosomes	HEK293T cells‐derived EVs, VSV‐G–GFP11, saCas9–GFP1‐10 and sgRNA	RNP	PINK1, PCSK9	∼40% reduction in the number of cells positive for Parkin recruitment (in HeLa‐Venus‐Parkin cells); significant reduction in serum PCSK9 and LDL cholesterol (in mouse liver)	[345]
**Cell‐penetrating peptides**					
Shuttle peptides	Engineered amphiphilic peptides (S10, S18, S85) noncovalently complexed with Cas9 (or Cas12a) protein and sgRNA	Cas9 RNP or Cas12a RNP	CFTR, HPRT1, ROSA26	∼26% (at the CFTR intron 22–23 in HAE with S10 peptide); ∼22% (at the HPRT1 locus in HAE with S10 peptide); ∼13% (large airways) and ∼12% (small airways) (at the Rosa26 locus in ROSA^mT/mG^ mice)	[346]
PF14 peptide nanoparticles	PepFect14 (PF14) cell‐penetrating peptide, PVA–PEG, Cas9, and sgRNA	RNP	Stop‐Light (SL) Reporter	∼80% (in HEK293T SL cells)	[347]
C2‐PF14 peptide nanoparticles	C2‐PF14 (C2 targeting peptide fused to PepFect14), PVA–PEG, Cas9, and sgRNA	RNP	SNCA (α‐synuclein)	∼42% (in SH‐SY5Y neuronal cells)	[348]
ADGN peptides nanoparticles	ADGN–AVA, ADGN–PEG peptides, and cargo mRNA	Cas9 mRNA and sgRNA	Firefly luciferase	∼60% (in A549‐luc cells)	[349]
A22p peptide	A22p peptide, spCas9 (fused with three copies of A22p peptide at C‐terminus), and sgRNA	RNP	Stop cassette in Ai9 tdTomato gene	∼70–72% (in turning on tdTomato with NPCs)	[350]
RALA peptide nanoparticles	RALA peptide, complex CRISPR components in pDNA, RNP, and mRNA molecular formats	Cas9/sgRNA pDNA; RNP; Cas9 mRNA and sgRNA	RFP	∼57.3% (in RFP^+^ primary MSCs)	[351]
6His–CM18–PTD4 peptide	6His–CM18–PTD4 peptide, Cas9 (or Cpf1), and sgRNA	Cas9 RNP or Cpf1 RNP	HPRT, DNMT1	Cas9–NLS RNP targeting HPRT: ∼13% in Jurkat cells and ∼18% in NK cells; Cpf1–NLS RNP targeting DNMT1: ∼12% in Jurkat cells and ∼27% in NK cells	[352]
**Intelligent‐responsive multifunctional scaffolds**					
Polyamines‐modified hydrogels	Poloxamer 407, poloxamer 188, polyamines (or hyaluronic acid), DOX (optional), Cas9 and sgRNA	RNP	YB‐1	∼53% (in B16F10 cells); ∼46% (in mouse melanoma model)	[353]
GelMA–HAMA hydrogel	GelMA, HAMA, APET×2 polypeptide, Cas9, and sgRNA	RNP	CFIm25	Effectively knocked down CFIm25 expression	[354]
Photothermal electrospun nanofibers	Polycaprolactone nanofibers, light‐sensitive iron oxide nanoparticles (IONPs), optional collagen or Geltrex coating, Cas9, and sgRNA	RNP	GFP, IL‐2R	∼80% (GFP protein knockdown in H1299‐GFP cells); >60% (IL‐2R gene knockout in hESCs)	[243]
MSCM‐coated nanofibril	Mesenchymal stem cell membrane, nanofibril, CXCL12α, LNP–Cas9 RNP	RNP	IL1RAP	∼53% (in THP‐1 cells)	[355]
Magnetic nanoparticles	Fe_3_O_4_ nanoparticles, polyethylenimine, and CRISPR/Cas9 plasmids	CRISPR/Cas9 plasmids	Traffic light reporter	∼13% (in HEK293‐TLR3 cells)	[356]
Magnetic nanoparticles	Magnetic core (ZnFe_2_O_4_), silica shell, PEI–Dye (inner layer), multiplasmids layer, and PEI (outer layer)	CRISPR/Cas9 plasmids	MeCP2	∼42.95% HDR (in RTT‐NPCs_(Q83X)_)	[357]

Abbreviation: CRISPR, clustered regularly interspaced short palindromic repeats.

Other emerging systems leverage endogenous vesicular pathways for enhanced biocompatibility. For example, exosomes derived from hepatic stellate cells serve as a delivery platform for Cas9 RNPs, achieving therapeutic efficacy in multiple liver disorder models with minimal immune responses [358]. Beyond these, CPPs provide a fully synthetic route for cytosolic access. Optimized CPP–Cas9 or CPP–mRNA conjugates enable direct translocation across membranes, achieving efficient editing in muscle and neuronal tissues while avoiding endosomal sequestration [359]. Intelligent‐responsive multifunctional scaffolds exhibit unique response characteristics in gene delivery, enabling precise release of genetic material according to the specific microenvironment for efficient and safe genome editing (Figure 4).

Collectively, these approaches signal a turning point in genome editing delivery. By integrating viral‐like efficiency with nonviral control and modularity, emerging vectors are approaching single‐dose, multiorgan editing with clinically relevant efficiency and safety. Although challenges in scalable manufacturing, precise biodistribution, and regulatory approval pose significant hurdles for clinical translation, these novel delivery platforms are emerging as cornerstone modalities in next‐generation gene therapy.

### 5.1 Virus‐Mimicking Nanosystems

Advances in virus‐mimicking nanosystems, including VLPs, self‐assembling endogenous systems such as SEND (Selective Endogenous eNcapsidation for cellular Delivery), and bacteriophage‐inspired contractile injection systems (CISs), have enabled transient, high‐efficiency, and low‐immunogenicity genome‐editing delivery [110, 332, 337, 338, 360]. These approaches retain the high uptake and cytosolic access typical of viruses while eliminating the genomic‐integration risks associated with replicating vectors. Collectively, they illustrate how precise engineering of structural proteins, envelopes, and fusion mechanisms can broaden tropism, enhance intracellular release, and improve translational feasibility.

VLPs are increasingly recognized as effective vehicles for delivering genome‐editing enzymes, owing to their propensity to incorporate and shield either nucleic acid or protein payloads, thereby simulating the viral infection efficiency without accompanying risks [21, 208, 361]. VLPs harness the self‐assembly of retroviral Gag polyproteins to encapsulate and deliver genome editing cargos. For example, fusing Cas9 to the HIV‐1 Gag protein enables the resulting VLPs to efficiently package and deliver Cas9 RNPs into target cells. This method ensures that the Cas9 RNPs are encapsulated within the VLPs, protecting them from degradation and enhancing delivery efficiency. Early Streptococcus pyogenes Cas9–Gag constructs achieved 15% editing efficiency in lymphoid cells [334]. Incorporating protease‐cleavable linkers, nuclear export signals, and optimizing the gag‐cargo: gag–pro–pol stoichiometry raised indel frequencies above 90% in HEK293T and Neuro‐2a cells. Recent advances are exemplified by the RIDE platform, which achieves efficient gene editing through optimized VLP design and enables cell‐type‐specific targeting via envelope engineering [109]. MLV‐derived eVLPs delivering ABEs corrected pathogenic alleles in human iPSCs and achieved therapeutically relevant editing in murine retina, liver, and brain following both local and systemic delivery [110]. Furthermore, Aptamer‐guided loading, maintaining ∼5–6 mRNA or RNP molecules per particle, effectively compensates for the absence of viral replication and sustains disease‐modifying outcomes [362, 363].

Envelope modifications further refine targeting and immune compatibility. While VSV‐G remains widely used for broad tropism, the cellular tropism of VLPs can be reprogrammed by pseudotyping them with different envelope glycoproteins, enabling the transduction of specific cell populations. For example, pseudotyping with the FuG‐B2 envelope glycoprotein confers specificity for CNS cells. In vivo, compared with VSV‐G‐pseudotyped vectors, FuG‐B2‐pseudotyped engineered VLPs mediated efficient base editing in mouse neuroblastoma cells but not in fibroblasts [110]. Similarly, pseudotyping Cas9–VLPs with the HIV‐1 envelope glycoprotein (Env) enabled specific targeting of CD4^+^ T cells within a mixed population of CD4^+^ and CD8^+^ T cells, while sparing bystander CD8+ cells [335].

Eliminating viral structural proteins altogether offers an additional route to mitigate immunogenicity. The emergence of the SEND system marks the development of a novel type of “humanized” VLPs [333]. This system innovatively employs PEG10, an endogenous retrotransposon‐derived protein from the human genome, to replace the structural components of traditional viruses. PEG10 is capable of naturally encapsulating mRNA via its untranslated‐region (UTR) motifs. Flanking Cas9 or other therapeutic transcripts with these UTRs allows PEG10 to assemble into RNA‐loaded vectors in producer cells [332]. Although fusogens are required in trans, SEND particles avoid anticapsid immune responses even after repeated dosing administration and achieve efficient editing in multiple tissues. Further evidence shows that after subretinal injection, this system can specifically transduce retinal pigment epithelial cells, achieving stable transgene expression for several consecutive weeks without eliciting significant inflammatory responses. These results underscore the immunological advantages of its humanized components [364]. Collectively, these findings confirm that SEND, as a modular and fully endogenous delivery platform, leverages endogenous human protein components to provide a novel delivery tool with low immunogenicity potential for gene therapy.

Beyond biologically derived shells, bacteriophage‐derived CISs offer a programmable design platform unconstrained by native viral biology. CISs employ a distinct, mechanically driven delivery mechanism: upon receptor recognition, their contractile sheath drives an inner tube through the plasma membrane to form a transient pore, releasing cargo directly into the cytosol. This membrane‐puncturing entry mechanism effectively bypasses endosomal entrapment and accommodates diverse functional payloads, including proteins, nucleic acids, and RNP complexes. Receptor specificity can be precisely reprogrammed by engineering targeting domains within tail fiber (e.g., Pvc13 in the Photorhabdus virulence cassette, PVC) or spike tip (e.g., Pvc10) proteins, enabling efficient delivery to otherwise refractory cell types. Recent work introduced the SPEAR (spike engineering and retargeting) strategy, which enables modular loading of proteins, RNPs, and ssDNA onto PVC spikes and covalent retargeting via SpyTag/SNAP to antibodies or nanobodies. This nanosyringe system efficiently delivers Cas9 RNPs and DNA templates into mammalian cells and specific immune‐cell populations in vivo with minimal off‐target effects. By incorporating targeting moieties such as adenoviral knob domains, DARPins, or nanobodies, the SPEAR‐engineered PVCs can be precisely redirected to human cells and live mice, underscoring their broad potential in gene therapy and biotechnology applications [337, 338, 365, 366]. Notably, SEND represents a fusion‐mediated route, while CISs achieve direct cytosolic penetration, two complementary solutions to the same intracellular barrier.

Taken together, VLPs, SEND particles, and CIS platforms define a new generation of programmable delivery technologies. Each addresses fundamental challenges in genome editing, cargo loading, tropism, immune compatibility, and cytosolic access, through distinct mechanisms. Future progress will depend on integrating receptor‐specific ligands with fusion or puncture‐based entry systems, refining encapsulation signals, and developing scalable manufacturing platforms for clinical translation.

### 5.2 Cell‐Derived Extracellular Vesicles

Cell‐derived extracellular vesicles (EVs), as endogenous LNPs, facilitate the transfer of proteins and nucleic acids between cells, offering a biocompatible endogenous alternative for gene editing delivery systems [367]. By leveraging their natural biosynthetic pathways, EVs hold the potential to not only overcome both extracellular and intracellular delivery barriers but also integrate seamlessly into human cellular signaling networks, highlighting their significant promise for targeted therapeutic applications. In addition, immune‐evasion is essential for vesicle persistence and activity. Exosomes naturally evade immune clearance through self‐antigenic membranes, a property enhanced by integrating immunomodulators such as CD47 [368]. Encapsulating rAAV within exosomal membranes provides partial protection against NAbs and allows codisplay of immune‐suppressive ligands; combined with IgG‐degrading enzymes such as IdeS, this strategy improves transduction in repeat‐treatment models [369, 370]. EVs are largely categorized into three groups: exosomes, microvesicles, and apoptotic bodies [371]. Exosomes, naturally secreted 50–150 nm vesicles derived from multivesicular endosomes, inherit the lipid and membrane protein profiles from their parent cells, providing a self‐derived “fingerprint” that minimizes opsonization and promotes fusion with homologous tissues [372]. For example, exosomes derived from hepatocyte AML12 cells accumulate in the liver and have been used to deliver CRISPR–dCas9–VP64 complexes to upregulate HNF4α and alleviate fibrosis [108]. Similar principles underlie synthetic LNPs targeting, where serum adsorption of ApoE directs intravenously administered LNPs to hepatocytes via LDL receptor interactions [373, 374]; the surface proteome of exosomes governs their organ tropism and can be re‐engineered for extra‐hepatic targets.

To improve scalability, hybrid approaches have fused exosomes with preloaded liposomes, combining the natural compatibility of exosomes with the loading capacity of synthetic bilayers. Such exosome–liposome chimeras have successfully delivered plasmid DNA and modified Cas9 mRNA [375, 376, 377]. For instance, the delivery of the sgRNA/Cas9 plasmid via AS1411–aptamer‐modified exosome and liposome chimeras resulted in 42.05–55.02% gene editing efficiency in endothelial cells, significant inhibition of choroidal neovascularization in a rabbit model, and no notable increase in intraocular inflammatory factors [325].

Building on advances in exosome‐based delivery technologies, engineered EVs now offer a more versatile and customizable framework for therapeutic genome editing. An all‐in‐one EV delivery system named NanoMEDIC has addressed the critical challenge of loading large functional proteins by using chemically induced dimerization to efficiently recruit and encapsulate Cas9 proteins into extracellular nanovesicles. This system demonstrated the remarkable capability to achieve over 90% exon‐skipping efficiency in skeletal muscle cells derived from DMD patient iPSCs, showcasing its high therapeutic potential for gene editing [340]. Notably, an engineered EV platform based on the Fc/Spa interaction and PTGFRN membrane anchoring achieved efficient enrichment of Cas9–sgRNA RNPs and targeted delivery to neural tissues. This system significantly suppressed HSV‐1 replication in vitro and in infected mice, while maintaining low immunogenicity and off‐target activity, highlighting its translational potential for EV‐mediated genome editing therapies [341].

An alternative EV subtype, arrestin domain‐containing protein 1‐mediated microvesicles (ARMMs), provides a versatile and controllable platform for intracellular delivery of therapeutic macromolecules [342]. By leveraging ARRDC1‐driven budding at the plasma membrane, ARMMs can selectively package diverse cargos, including p53 protein, mRNAs, and CRISPR–Cas9/sgRNA complexes, via engineered peptide or domain interactions. Remarkably, ARMMs‐delivered p53 restored apoptosis in p53‐deficient mice, and Cas9 RNPs mediated efficient gene editing in recipient cells, underscoring their broad potential for in vivo protein and RNP delivery [343].

Furthermore, protein‐tethering strategies, such as fusing the tetraspanin CD9 to the RNA‐binding protein HuR, enrich sgRNA and dCas9 transcripts within vesicles, yielding higher nuclease encapsulation and more consistent release [378]. The integration of endogenous cues, active cargo loading, surface ligand engineering, and immune‐evasion strategies has substantially enhanced vesicle‐based genome‐editing platforms. Each innovation addresses distinct delivery barriers, from cargo packaging to immune compatibility, yet their modular integration enables versatile and disease‐specific therapeutic designs.

### 5.3 Cell‐Penetrating Peptides

Cationic CPPs have emerged as versatile, nonviral platforms for RNP delivery, circumventing the size limits and immunogenicity of viral vectors while maintaining the intrinsic efficiency of RNP‐based genome editing. Among the diverse CPPs, three major physicochemical subtypes (arginine‐rich, amphipathic, and nucleic‐acid‐binding peptides) have been most intensively studied, each conferring distinct advantages for membrane interaction, intracellular trafficking, and functional release.

Arginine‐rich CPPs exploit guanidinium side chains to form multivalent electrostatic interactions with both nucleic acids and anionic membrane lipids, driving spontaneous complexation with Cas9 RNPs and enabling translocation through direct penetration or macropinocytosis. Their efficiency is linked to transient water pore formation and local perturbation of lipid packing, which facilitate cytosolic entry [379]. Structural optimization, such as introducing hydrophobic residues, can improve uptake but must balance against cytotoxicity at higher peptide doses, highlighting the need for rational sequence engineering [380].

Amphipathic CPPs incorporate hydrophobic residues into cationic frameworks, strengthening bilayer and promoting endosomal escape. These peptides stabilize RNPs in serum, protect them from proteolysis, and enhance membrane fusion. Demonstrations in airway epithelia confirm their potential for gene therapy. In particular, engineered amphiphilic shuttle peptides have been shown to efficiently deliver proteins and CRISPR–Cas RNPs to human and mouse airway epithelial cells both in vitro and in vivo, enabling functional gene editing with minimal toxicity [346]. Amphiphilic block copolymers that self‐assemble into micelles around Cas9 RNP show similar benefits [381], underscoring amphiphilicity as a general design principle for serum‐stable, membrane‐active carriers.

Nucleic‐acid‐binding CPPs extend delivery capability through higher‐order assemblies. Poly(disulfide)‐based nanoparticles compact RNPs extracellularly and release them upon intracellular reduction [382], while platforms such as CRISPR‐gold employ thiol‐DNA modified gold cores to codeliver Cas9 RNPs and donor templates, achieving targeted in vivo editing [383]. Although gold nanoparticles are not peptides, their dense nucleic acid binding parallels strategies and suggests opportunities for hybrid assemblies.

Advances in CPP performance increasingly leverage site‐specific chemical modification. PEGylation creates a hydrophilic corona that shields peptide–RNP complexes from proteases and opsonins, extends circulation, and reduces aggregation without abrogating membrane activity [347, 348]. Conjugation with nuclear localization sequences (NLS) addresses the nuclear import barrier, as shown in the GEDEX system where dCas9/VPR complexes were efficiently coranslocated into the nucleus [384]. Together, these modifications couple membrane translocation with nuclear targeting to achieve more complete intracellular routing.

The mechanistic bases underpinning CPP performance continue to be elucidated through emerging research. α‐Synuclein‐based carriers, for example, exploit α‐helical transitions to improve bilayer penetration and enable both endosomal and nonendosomal uptake [385]. Genetic knockout studies confirm that transient pore formation underlies direct translocation, while modulation of hydrophobicity influences cell‐type specificity [380]. However, variability in membrane composition across tissues remains a key translational challenge.

The direct delivery of CRISPR RNPs with CPPs has significantly advanced genome editing outcomes, as both covalent and noncovalent peptide–RNP complexes have demonstrated higher editing efficacies and reduced cytotoxicity relative to nucleic acid‐based exposures [107, 349, 386]. Modified CPP motifs enhance editing efficiency in difficult‐to‐transfect cells such as neural progenitors [350], and amphiphilic formulations like RALA expand the utility across DNA, RNA, and RNP formats [351]. Histidine‐rich peptides improve routing by transient plasma membrane anchoring [352], and self‐assembling peptide nanocomplexes such as ADGN peptide achieve high efficiency gene knockout and selective tumor targeting [349]. In addition, incorporation of stabilizing components, such as inorganic ions or PEG chains, enhances nanostructure integrity and cargo release in physiological conditions [387, 388]. Compared with lipid‐based carriers, peptide assemblies often exhibit superior biocompatibility and modularity, though their complex synthesis and potential immunogenicity complicate scale‐up.

Systemic and localized administrations further confirm clinical promise. Intravenous CPP–RNP complexes have achieved tumor‐specific disruption with low off‐target effects [389], while intranasal or intravaginal dosing has enabled editing in airway and endometrial tissues [388, 390, 391]. Nonetheless, incomplete biodistribution, immune responses upon repeat dosing, and interspecies variability remain barriers requiring improved targeting ligands, stimuli‐responsive coatings, and predictive animal models.

Collectively, CPPs provide a modular toolkit for genome‐editing delivery. Arginine‐rich, amphipathic, and nucleic acid‐binding classes confer complementary strengths in condensation, protection, and cytosolic trafficking. Rational engineering, through sequence design, PEGylation for serum stability, and NLS conjugation for enhanced nuclear targeting, continues to improve safety and potency. While challenges in endosomal escape, manufacturing reproducibility, and in vivo predictability persist, accumulating evidence positions CPPs as leading nonviral carriers with broad biomedical and biotechnological potential.

### 5.4 Intelligent‐Responsive Multifunctional Scaffolds

Spatiotemporal control of genome‐editing activity has become a defining design principle for next‐generation delivery platforms. By integrating CRISPR components into materials responsive to biochemical cues or external physical fields, these platforms aim to confine nuclease activity to diseased tissues, minimize systemic exposure, and synchronize editing with regenerative processes. Representative examples include hydrogels, scaffolded nanofibers, and magnetically responsive complexes, which collectively exemplify the emergence of “on‐demand” genome‐editing release systems.

Hydrogel‐based systems utilize hydrated, cross‐linked polymer networks to encapsulate RNP and release them in response to pathological signals such as ROS or acidic pH. Beyond biochemical responsiveness, thermosensitive hydrogels have also been harnessed for localized control. For instance, a polyamines‐modified thermosensitive hydrogel (Psh) was developed for the intratumoral codelivery of CRISPR/Cas9 RNP (targeting the YB‐1 gene) and doxorubicin, demonstrating sustained release, enhanced tumor accumulation, and potent antimelanoma efficacy with minimal off‐target effects [353]. Similarly, phenylboronic acid‐modified PEI nanoparticles have been enveloped by detachable hydrogel shells that remain stable in circulation but disassemble in ROS‐rich, acidic tumor microenvironments [392]. Such conditional degradation protected genome‐editing cargos from premature degradation while ensuring localized activity, thereby reducing systemic toxicity. Beyond tumor targeting, supramolecular peptide hydrogels have been engineered to support immunotherapy, sustaining CAR‐T cell activity and promoting memory T‐cell formation in mouse models [393]. Composite hydrogel‐nanofiber scaffolds loaded with CRISPRa complexes further demonstrated multifunctionality: applied in a rat wound‐healing model, they enabled VEGF upregulation, angiogenesis, and tissue regeneration while avoiding viral vector safety concerns [394]. However, the influence of scaffold geometry, cross‐linking density, and mechanical cues on long‐term outcomes remains to be fully delineated.

Whereas hydrogels excel in volumetric encapsulation, scaffolded nanofibers contribute directional architecture, integrating mechanical guidance with localized molecular release [355, 395]. Advancing this concept, photothermal electrospun nanofibers have been engineered to leverage the photothermal effect of embedded iron oxide nanoparticles. Upon near‐infrared laser irradiation, this platform enables contactless membrane permeabilization, allowing it to achieve high editing efficiency while mitigating the safety concerns associated with direct nanoparticle internalization [243]. Electrospun fibers coated with self‐assembling peptide layers have been used to deliver CRISPR–dCas9 activators to primary neurons, sustaining intracellular levels of dCas9/VPR over 7 days and enhancing neurogenesis [396]. Alignment of fibrous scaffolds with neurite outgrowth created synergistic benefits, suggesting value for neural regeneration. Yet, validation beyond neuronal cultures is required, particularly in inflamed or heterogeneous tissue environments where scaffold degradation and biointegration may vary.

Magnetically responsive complexes contribute an additional dimension of spatiotemporal control. Superparamagnetic iron oxide nanoparticles (SPIONs) can be magnetically guided to deep tissues, with controlled drug release achieved through magnetothermal effects [397]. SPIONs coated with PEI and complexed with the CRISPR/dCas9 system leverage their surface positive charge to significantly enhance transfection efficiency in human skin fibroblasts, enabling targeted gene activation without significant cytotoxicity [398]. A key advancement in this area is the magnetic nanoparticle‐assisted genome editing platform, which synergizes magnetofection with magnetic‐activated cell sorting to achieve 42.95% HDR efficiency in correcting the MeCP2 mutation in Rett syndrome patient‐derived neural cells [357]. In a mouse model of myocardial infarction, magnetic targeting enabled the efficient accumulation of CRISPR/Cas9–SPIONs in the ischemic heart, leading to significant miR34a gene knockdown and subsequent cardiac repair with minimal off‐target effects observed [399]. Their modularity also permits codelivery of contrast agents for real‐time monitoring, though field penetration limits and the need for specialized equipment constrain clinical scalability.

Complementary strategies are emerging through stimuli‐responsive and optogenetic systems. Photoswitchable RNA‐binding proteins such as LicV enable reversible CRISPR activation with light [400], while smart CRISPR/Cas constructs have been designed to respond to temperature, pH, or enzymatic triggers [401]. Although multistimuli integration promises higher precision, it requires careful calibration to prevent premature activation in heterogeneous tissue environments.

Despite the demonstrated potential of these approaches, significant barriers remain for clinical translation. Uniform RNP distribution in dense matrices remains difficult, pathological ROS levels overlap with those of inflamed normal tissues, and magnetic field penetration is limited in deep organ targeting. Future directions may emphasize multiresponse, for example, hydrogel or nanofiber systems that can simultaneously decode multiple pathological signals, and the integration of magnetic targeting with imaging to enable adaptive pulse‐controlled drug delivery. In summary, hydrogels, nanofibers, and magnetic complexes represent a trajectory for precise genome editing, in which editing activities are spatially and temporally coordinated to align with the complex biology of human disease.

## 6 Design Considerations and Key Challenges

Successful translation of genome‐editing technologies into therapeutics depends on the development of delivery vehicles that are safe, efficient, and manufacturable. Whether the carrier is a viral vector, LNPs, polymer complexes, or VLPs, several recurring barriers consistently limit clinical performance. These challenges encompass molecular stability, payload capacity, tissue targeting, immunogenicity, expression kinetics, and large‐scale production. The foremost constraint arises from the intrinsic fragility of RNA components. Guide RNAs are rapidly degraded by serum nucleases, substantially reducing delivery efficiency. Chemical stabilization at the ribose 2′ position, such as 2′‐O‐methyl and PS modifications, protects guide RNAs from nuclease attack and markedly improves editing efficiency across diverse delivery platforms. Because these modifications are intrinsic to the RNA itself, they are broadly compatible with viral vectors, LNPs, and electroporated RNPs formats alike. A second pervasive limitation concerns the large size of many editing machineries, which often exceeds the packaging capacity of existing vectors. Oversized plasmids impede efficient polymerase–DNA complex formation, whereas bulky RNPs reduce nanoparticle encapsulation efficiency. Miniaturized or split editors help relieve these vector‐size constraints, increasing compatibility with compact viral capsids and high‐loading nanoparticle systems [118, 402, 403, 404, 405]. In addition, achieving therapeutic concentrations in specific organs remains one of the most formidable challenges. AAV biodistribution is strongly influenced by both species and serotype [19, 406, 407], whereas conventional LNPs are preferentially internalized by hepatocytes through ApoE–LDLR uptake [12, 373]. Peptide grafting, domain swapping, and lipid‐component tuning have enabled the generation of capsids and nanoparticles with enhanced cardiac, neural, or pulmonary tropism, facilitated by high‐throughput barcoding screens and data‐driven selection strategies. Immunogenicity constitutes a fourth overarching barrier. Up to 80% of adults carry pre‐existing NAbs against AAV, predisposing individuals to complement activation and dose‐dependent thrombotic microangiopathy (TMA) at high vector loads [44, 161]. Similarly, LNPs may also trigger acute cytokine release due to innate immune sensing of lipid components or RNA cargo. Mitigation strategies include transient immunosuppression, capsid shielding, and CpG depletion of payloads to attenuate both innate and adaptive immune activation [408]. Expression duration must balance efficacy and safety: transient delivery via RNPs or LNPs confers lower risk of off‐target activity compared with sustained expression driven by viral vectors, which may elicit T‐cell responses or increase the likelihood of integration events. Meanwhile, scalable manufacturing remains critical for clinical feasibility, requiring strict control over polymer cross‐linking, lipid composition, and particle uniformity to maintain potency and reproducibility at industrial scale.

### 6.1 Delivery and On‑Target Editing Efficiency

Gene editing efficiency is determined by the number of active editing complexes that reach the nucleus and the duration of their functional persistence. Across delivery formats, editing outcomes scale quantitatively with nuclear availability. Chemical stabilization of sgRNA illustrates this principle: introducing 2′‐O‐methyl and PS modifications at the termini of sgRNA increased gene editing in K‐562 cells from undetectable to ∼50% when coelectroporated with Cas9 mRNA, confirming that a minimal, specific modification pattern can yield major gains in efficiency under these conditions [4]. Preassembled Cas9–sgRNA RNPs consistently produce higher nuclear concentrations and indel rates than plasmid or viral vectors. In vivo, hepatocyte‐targeted LNPs delivering Cas9 mRNA and highly modified sgRNA achieved ∼70% editing of the Ttr locus with a 3 mg kg^−1^ dose, resulting in >97% serum TTR reduction maintained for at least 12 months [409].

Chromatin architecture imposes an additional layer of constraint. Nucleosomal occupancy and the conformational requirements of RNA–protein complexes raise the minimal number of correctly folded RNPs required per target site [410, 411]. Sub‐stoichiometric nuclear delivery fails to displace nucleosomes, whereas sufficiently high editor copy numbers overcome chromatin barriers and restore target accessibility. Enhancing nuclear localization can partially compensate for limited systemic exposure. rAAV genomes naturally access the nucleus, enabling dual‐AAV systems that reconstitute large editors postdelivery. In the DMD mouse model, split‐intein ABEs administered intramuscularly restored dystrophin and achieved 3.3% editing in cardiomyocytes [412], a notable outcome in nondividing tissue. By contrast, nonviral nanoparticles must overcome multiple intracellular barriers. Following endocytosis, most nanoparticles are trafficked to lysosomes, where nucleases rapidly degrade exposed cargo [413]. pH‐triggered phospholipid vectors that undergo endosomal phase transitions can disrupt membranes via the proton‐sponge effect, thereby enhancing cytosolic release [414]. But nuclear pores restrict passive diffusion to proteins <40 kDa, necessitating mitotic entry or efficient NLS engagement for larger RNPs and plasmids [415, 416, 417]. In postmitotic cells such as neurons and retinal pigment epithelial cells, optimized NLS presentation or capsid‐mediated transport is essential.

Ultimately, therapeutic editing necessitates achieving a nuclear threshold of active editor complexes while maintaining chromatin accessibility. Effective strategies will thus require integrated engineering across biodistribution, endosomal escape, and nuclear import mechanisms to ensure that both editor copy number and precise nuclear localization collectively support durable, precise genome editing.

### 6.2 Specificity and Off‑Target Accumulation

Precise control over biodistribution remains a major challenge for therapeutic genome‐editing vectors, as systemic administration typically directs most nanoparticulate carriers toward filtration organs. Intravenous LNPs exemplify this constraint: within minutes of administration, a large proportion are opsonized by ApoE, triggering LDLR‐mediated uptake by hepatocytes and leading to predominant liver uptake. While advantageous for targeting hepatocyte genes such as PCSK9 or TTR, this pathway restricts extrahepatic delivery and raises concerns regarding hepatic toxicities.

Chemical tuning of formulation components provides a primary means of reshaping biodistribution. Modifying the ratios or structural variants of cholesterol, PEG–lipids, and helper lipids modulates protein‐corona formation and tissue tropism. Anionic helper lipids promote the Stab2‐dependent uptake by scavenger endothelial cells, whereas Stab2 deficiency markedly attenuates this clearance pathway and prolongs systemic circulation [418]. Variations in PEG–lipid architectures can shift nanoparticle uptake between vascular and parenchymal compartments, and incorporation of dichloroacetic‐acid motifs redirects trafficking toward the lung and heart [254]. Collectively, subtle chemical modifications can reprogram organ preference without changing the genetic payload.

Active‐ligand targeting adds an additional layer of specificity. Triantennary GalNAc ligands conjugated to LNPs selectively bind the hepatocyte ASGPR receptor; a single administration of GalNAc–LNPs encapsulating base‐editor mRNA and ANGPTL3‐targeting sgRNA reduced circulating ANGPTL3 by >90% [419]. Antibody‐conjugated systems extend this precision beyond the liver: anti‐CD5 scFv‐conjugated LNPs enabled in situ generation of CAR‐T cells directly from circulating lymphocytes [420]. Meanwhile, SORT chemistry reprograms helper‐lipid charge to redirect LNPs from liver to lung or spleen, achieving ∼15% PTEN editing in pulmonary tissue [254]. Collectively, these strategies demonstrate how rational lipid and ligand engineering can redistribute editing activity while maintaining potency.

Biological vectors such as exosomes complement synthetic systems through intrinsic immune stealth and natural tissue tropism. Hepatocyte‐derived vesicles preferentially home to the liver, whereas surface engineering with receptor‐binding peptides or antibodies confers disease‐specific targeting without introducing foreign synthetic materials. Hybrid exosome–liposome constructs further improve cargo loading and support localized delivery with reduced innate immune activation [376, 421].

Microenvironment‐responsive formulations introduce an orthogonal layer of selectivity. Phenylboronic acid‐modified PEI nanoparticles encapsulate CRISPR–dCas9 plasmids within ROS‐ and pH‐labile shells that remain PEG‐masked in circulation but disassemble within acidic, oxidative tumor niches, releasing editors only at disease sites. Similarly, peptide‐enriched LNPs exhibit enhanced cardiac and splenic penetration, though hepatic uptake remains substantial [422, 423]. Combining such stimuli‐responsive chemistries with ligand‐based targeting may further enhance organ specificity. Surface charge and shielding also govern stability and off‐target exposure. Highly cationic particles exhibit excessive protein adsorption and rapid clearance, whereas neutral or densely PEGylated carriers show improved colloidal stability and reduced endothelial binding. PP/PEI coronas can attenuate hepatic tropism and enable editing in heart and lung. Balancing control over charge, hydrophobicity, and stealth coatings therefore remains essential for safe systemic administration.

Collectively, organ‐selective genome editing requires harmonizing chemical composition, targeting ligands, biological vectors, and microenvironmental responsiveness. Integrating these parameters enables programmable biodistribution and minimizes off‐target accumulation, paving the way toward tissue‐precise and clinically viable genome‐editing therapeutics.

### 6.3 Immunogenicity and Innate Immune Activation

Immune recognition remains a major obstacle to the safety and durability of therapeutic genome editing. Complement activation, cytokine release, and adaptive immunity to editor proteins constitute interconnected defense layers that can severely compromise efficacy. These immune mechanisms are most evident in high‐dose AAV therapies but increasingly observed with emerging lipid‐ and nanoparticle‐based systems, underscoring the need for broad‐spectrum mitigation strategies.

At high systemic doses, AAV vectors can trigger complement‐mediated toxicities including thrombocytopenia, hemolytic anemia, elevated liver enzymes, and in severe cases, fatal TMA [161]. Clinical experience with AAV9 (onasemnogene abeparvovec) and AAV‐based DMD gene therapies has linked these effects to activation of the classical complement pathway: antibody‐capsid complexes recruit C1q, deplete C4, and initiate membrane‐attack complex formation that injures vascular endothelium. However, observations of C4 depletion prior to detectable anti‐AAV antibodies in some seronegative recipients, and transient complement activation in NHPs despite similar dosing, suggest additional triggers, potentially direct capsid–complement interactions or species‐specific Fc‐receptor differences [424, 425, 426]. These findings emphasize the necessity of patient‐specific risk assessment in AAV gene therapy. Current clinical countermeasures target distinct complement cascade components. The C5 inhibitor eculizumab reduces sC5b‐9 formation and mitigates platelet loss, while C3 inhibitor (APL‐941) and C1 esterase (APL‐9) inhibitors aim to block upstream activation. Enzymes such as IceM and IceMG remove initiating IgM and IgG, though their therapeutic window remains undefined [427]. In parallel, vector‐centered strategies, reducing total vector dose via enhanced capsid potency or minimizing C1q‐binding epitopes, represent key preventive strategies.

Complement activation frequently coincides with innate immune signaling that induces systemic cytokine release syndromes. Genome‐editing vectors activate Toll‐like receptors (TLR2, TLR9) and cytosolic RNA sensors including MDA5 and RIG‐I, resulting in rapid secretion of interferons and proinflammatory cytokines [428]. In clinical contexts, these responses can precipitate capillary‐leak syndrome even in the absence of adaptive immunity. Cytokine levels typically peak within 6 h postinfusion, a window during which innate signaling suppresses transgene expression and primes adaptive responses. To mitigate these effects, prophylactic regimens such as dexamethasone, paracetamol, and antihistamines administered before infusion of the siRNA–LNP drug patisiran have proven effective.

Adaptive responses represent the most persistent challenge. NAbs against natural AAV serotypes are prevalent in up to 80% of adults, preventing efficient transduction and restricting repeated dosing [429]. Cytotoxic CD8^+^ T cells recognizing capsid‐ or transgene‐derived peptides further limit durability, causing clearance of edited cells and tissue‐specific pathologies such as dorsal‐root‐ganglion inflammation and myocarditis [430, 431]. Several molecular strategies reduce these adaptive immune responses. Incorporating antigen‐presenting cell (APC)‐specific miRNA binding sites (e.g., miR‐142 or miR‐652) into expression cassettes suppresses transgene expression in APCs, decreasing peptide presentation and T‐cell priming [432]. Including a miR‐183 site selectively detargets dorsal root ganglion expression, reducing sensory toxicity in preclinical models [433]. Codon deoptimization to eliminate CpG motifs diminishes TLR9‐mediated cross‐priming. For editor proteins such as Cas9, targeted deletion of immunogenic epitopes has eliminated humoral and cellular responses in mice, demonstrating that epitope engineering can complement pharmacological immunosuppression [10]. At the vector level, sequential administration of orthogonal serotypes (e.g., AAV8 followed by AAV5) or induction of tolerance to capsid epitopes helps overcome redosing limitations [434].

No single approach adequately addresses the complex interplay between innate and adaptive responses. Complement inhibition alone cannot prevent cytotoxic T‐cell infiltration, while corticosteroids may fail to suppress delayed neuro‐ or cardiotoxicity. Conversely, molecular detargeting prolongs expression only if early complement and cytokine cascades are controlled. The emerging consensus supports a two‐stage immunomodulation paradigm: acute management of innate activation, through complement and cytokine suppression at dosing; long‐term tolerance achieved by molecular detargeting, CpG minimization, and epitope engineering. Integrating these layers with rational vector design will be essential to ensure both immediate safety and durable therapeutic benefit in genome‐editing interventions.

### 6.4 Transient Versus Persistent Expression and Safety

The persistence of nuclease activity within target cells is primarily dictated by the delivery modality. Viral vectors such as AAV and lentivirus deliver DNA that remains episomal or integrates into the host genome, enabling transgene expression from weeks to years [435]. In contrast, preassembled Cas9–sgRNA RNPs are rapidly degraded by endogenous nucleases and proteases, restricting genome‐editing activity to a timeframe of only several hours [107]. This dichotomy establishes two distinct safety paradigms that now frame therapeutic genome‐editing design.

Sustained expression through viral vectors offers operational advantages, including high transduction efficiency, broad tissue tropism, and the potential for single‐dose efficacy. However, prolonged nuclease exposure substantially increases the probability of off‐target cleavage, particularly at low‐affinity genomic sites that may evade detection in short‐term assays. Large‐animal studies show that AAV genomes can integrate at DSB loci with a frequency comparable to wild‐type AAV, with the liver frequently serving as an integration hotspot [436]. Even integration‐deficient LVs yield measurable insertion events [437]. Such integrations raise clinical concerns due to the potential inactivation of tumor suppressors or activation of proto‐oncogenes. Moreover, AAV‐associated dose‐dependent hepatotoxicity demands careful balancing of editing efficacy against long‐term safety risks [438].

Transient RNP delivery mitigates many of these liabilities by confining nuclease to a narrow temporal window [110]. The absence of exogenous DNA eliminates vector‐integration risk, and off‐target cleavage declines rapidly as RNPs are cleared. Preformed complexes provide stoichiometric control over nuclease and guide RNA, and enable the application of high‐fidelity or self‐terminating variants such as Cas9TX, that reduce translocation formation. Emerging transient platforms, particularly VLPs, achieve efficient editing across multiple tissues while maintaining rapid nuclease clearance [7, 110, 111, 439, 440]. These VLPs combine the safety advantages of transient exposure with delivery efficiencies approaching viral vectors. Despite their favorable safety profile, RNPs are chemically unstable and unable to cross cellular membranes without assistance. They therefore require carrier systems capable of shielding against extracellular degradation, facilitating endosomal escape, and ensuring cytosolic release [110]. Clinical data now demonstrate that a single administration of LNP‐encapsulated RNP can achieve durable gene silencing in humans, validating the translational feasibility of transient delivery strategies.

Ultimately, overall genotoxic risk depends on both the persistence of nuclease expression and the inherent hazards associated with inducing DSBs. Sustained viral expression amplifies the probability of off‐target cleavage, vector integration, and chromosomal rearrangements, whereas transient RNP systems confine these events to a short and controllable window. Nonetheless, even brief nuclease activity can trigger deleterious structural variants, emphasizing the need for continued refinement of delivery vehicles. The development of self‐limiting viral vectors, next‐generation capsids that enable therapeutic editing at lower doses, and optimized transient‐RNP carriers will be essential to ensure durable efficacy with minimized long‐term risk.

### 6.5 Manufacturing

Scalability, the capacity to translate a delivery system from bench‐scale synthesis to industrial good manufacturing practice (cGMP) manufacturing while maintaining potency and safety, has become a decisive benchmark for genome‐editing platforms. Vectors that can be produced at industrial scale, administered at therapeutic doses, and adapted across indications are most likely to achieve broad clinical adoption. Chemically synthesized components inherently meet these criteria: short oligonucleotides bearing 2′‐O‐methyl or PS linkages are manufactured via solid‐phase chemistry, supporting kilogram‐scale production reproducibility under cGMP conditions. LNPs similarly represent a mature, scalable nonviral delivery platform [14]. Microfluidic mixing yields uniform LNPs within minutes and is now industrialized for mRNA vaccines, producing multikilogram batches for global clinical trials [441].

Small‐ligand conjugates further streamline manufacturing by reducing formulations to two component. The GalNAc–siRNA drug *givosiran*, produced via automated oligonucleotide synthesis followed by a single conjugation step [442], exemplifies lot‐to‐lot consistency and simplified fill‐finish operations, avoiding the colloidal complexity of nanoparticle formulations. In contrast, viral vectors, particularly rAAV, remain constrained by dose‐dependent scalability limits [10]. Clinical regimens require 10^14^–10^15^ vector genomes per patient, demanding batch‐scale production >2 × 10^16^ genomes. Comparative studies indicate that insect‐cell (Sf9)‐derived rAAV achieves superior full‐to‐empty particle ratios and lower costs than transient HEK293 production, while the TESSA helper‐adenovirus platform synchronizes Rep and Cap expression to increase rAAV2 and rAAV6 titers several‐fold without compromising purity [443, 444]. Vector re‐engineering can offset manufacturing burdens by reducing required dosing. Directed evolution has yielded rAAV.cc47 with enhanced brain and heart transduction and MyoAAV variants with improved skeletal‐muscle tropism [18, 407]. Compact editors such as Cas12f, along with split‐intein BEs capable of postinfection reassembly, allow packaging within a single AAV capsid, reducing total viral load requirements two‐ to fourfold [405].

VLPs bridge viral and nonviral systems, coupling transient expression with high delivery efficacy. Continuous bioreactor cultivation and optimized Gag‐cargo ratio have increased titers to ≥10^15^ particles for large‐animal studies, though yields still lag several orders of magnitude behind rAAVs production [110]. Nonviral polymers and dendrimers provide cost‐effective, flexible alternatives for scalable delivery. HPAE vectors, synthesized via one‐pot Michael addition, maintain up to 70% transfection efficiency in vitro and support nebulized lung delivery in rodents, using inexpensive monomers and an aqueous, multiliter reactor [307, 445, 446]. Degradable star polymers, hydroxyl‐rich lipids, and PEI–graphene‐oxide hybrids similarly combine potent transfection with scalable solution‐phase synthesis, while avoiding the sterile‐filtration bottlenecks of lipid emulsions.

Future scalability will rely on modular, data‐driven production platforms, such as machine learning (ML)‐guided promoter design, barcoded in vivo screening pipelines, and adaptive nanoparticle shells, that enable rapid optimization without extensive manufacturing redesign.

### 6.6 Regulatory and Clinical Translation Landscape

The regulatory landscape for therapeutic genome editing is defined by rigorous oversight frameworks established by agencies such as the US FDA and EMA, which govern all stages of delivery‐system development from discovery to clinical manufacturing. These frameworks converge on three core principles that shape translational design. First, genome‐editing machinery must remain as simple and transient as possible, as prolonged nuclease expression increases the risk of off‐target cleavage and genotoxicity, thereby requiring enhanced safety monitoring. Second, comprehensive in vivo biodistribution data are mandatory because organ tropism cannot be inferred solely from murine studies. Third, delivery systems are held to the same chemistry, manufacturing, and controls (CMC) standards as the editing payload, including stringent identity, purity, and potency testing throughout scale‐up [447].

Investigational new drug submissions begin with a delivery system discovery and optimization pipeline, in which viral or nonviral candidates are screened in vitro for efficacy and cytotoxicity before progressing through rodent and larger‐animal models. Cross‐species validation of both biodistribution and therapeutic index serves as a regulatory benchmark, as demonstrated by the development of liver‐targeted LNPs for NTLA‐2001, where preclinical data informed clinical starting doses and safety margins [9]. Potency assays are central to CMC evaluation. Regulators accept multiple assay formats provided they are quantitative, stability‐indicating, and mechanistically relevant. For rAAV vectors, potency is commonly established by in vitro or ex vivo transduction followed by measurement of editing frequency or therapeutic protein activity, exemplified by the rAAV5–FVIII program that led to approval of valoctocogene roxaparvovec, where FVIII clotting activity in human hepatocytes served as the release criterion [448]. For LNP‐mediated editors, reductions in circulating biomarkers such as TTR are often used as potency endpoints [9]. Immunogenicity testing has likewise become an essential regulatory requirement [449]; many AAV trials now incorporate cell‐based transduction inhibition assays to quantify the impact of patient serum on vector performance, informing enrolment thresholds and product‐release specifications.

Despite progress in single‐dose efficacy, redosing remains a major challenge, particularly for viral vectors. A single AAV administration induces serotype‐specific NAbs that block subsequent dosing, prompting regulators to closely examine mitigation strategies such as sequential dosing with orthogonal serotypes (e.g., AAV8 followed by AAV5), transient B‐cell suppression, tolerance‐induction protocols, and enzymatic degradation of circulating IgG using agents such as imlifidase, already approved in transplantation [408, 434]. Each approach requires extensive immunotoxicology and biodistribution evaluation before redosing can be authorized. Nonviral platforms, although less immunogenic, face their own repeat‐administration barriers. Multiple LNP infusions can trigger complement‐activation‐related pseudo‐allergy and induce anti‐PEG antibodies. Accordingly, the US FDA recommends monitoring complement split‐products and anti‐PEG titers in repeat‐dose toxicology studies. Moreover, because many LNPs rely on ApoE‐mediated hepatic uptake via LDL receptors, high or frequent dosing may saturate these pathways and alter systemic pharmacokinetics [12], necessitating investigation of such nonlinearity in preclinical models.

Collectively, the US FDA and EMA provide a demanding but predictable regulatory pathway for therapeutic genome editing. The rising number of approved AAV‐ and LNP‐based products indicates that these standards are achievable. Ultimately, regulatory success requires integrated datasets encompassing CMC validation, potency, biodistribution, and immunogenicity, all converging to show that a delivery system can achieve efficient, tissue‐specific editing following a single administration, while maintaining safety and providing well‐justified strategies for immune management and redosing feasibility.

### 6.7 Ethical

Recent first‐in‐human trials have confirmed that both viral and nonviral platforms can achieve clinically meaningful genome editing, while simultaneously exposing broader ethical and societal challenges related to safety, immunogenicity, manufacturing consistency, equitable access, and translational predictability. Public confidence is closely tied to perceived safety. For example, a Phase I study of NTLA‐2001 demonstrated that a single 0.3 mg kg^−1^ infusion reduced circulating TTR by an average of 87% within 28 days without serious adverse events [9]. In contrast, high‐dose AAV trials have triggered complement‐mediated TMA and T‐cell infiltration in dorsal‐root ganglia, raising concerns of neurotoxicity [161]. These findings underscore a public preference for transient, nonintegrating systems such as LNPs, while emphasizing the need for strict dose control and intensive monitoring in AAV‐based therapies.

To enhance controllability, researchers are embedding regulatory circuits into viral constructs. Rapamycin‐responsive promoters, riboswitch‐regulated poly(A) signals, and miRNA target motifs (e.g., miR‐142, miR‐183) enable pharmacological or cell‐type‐specific regulation of transgene expression, thereby mitigating oncogenic or germline transmission risks and aligning vector design with ethical expectations for reversibility and precision [450, 451, 452, 453].

Equitable access represents another ethical priority. Geographic, age‐related, and serotype‐dependent variations in AAV seropositivity complicate trial eligibility [44], potentially excluding large patient populations. Countermeasures such as transient B‐cell depletion, sequential serotypes switching, or short‐term immunosuppression with corticosteroids and rapamycin are under investigation, while nonviral platforms may circumvent such barriers entirely. Notably, the base‐editing LNP therapy VERVE‐101 achieved a sustained 55% reduction in LDL cholesterol for 6 months following a single 0.6 mg kg^−1^ dose, demonstrating the feasibility of redosing‐independent, immune‐evasive gene editing [454].

Translational predictability across species remains essential for societal trust. Although small‐animal studies often overestimate efficacy, cross‐species and humanized systems are improving reliability. Examples include rAAV.cc47, which preserves strong cardiac and neural tropism in primates, and AAV3‐based LK03, which exhibits superior human hepatocyte transduction compared with murine‐tropic serotypes [407, 455].

Ultimately, the ethical future of genome editing will depend as much on social acceptance as on technological progress. Delivery systems that demonstrate reproducible manufacturing, controllable expression, manageable immune profiles, and consistent cross‐species performance will form the foundation of public trust, equitable access, and responsible clinical translation.

## 7 Disease‐Specific Delivery Case Studies

**FIGURE 5:**
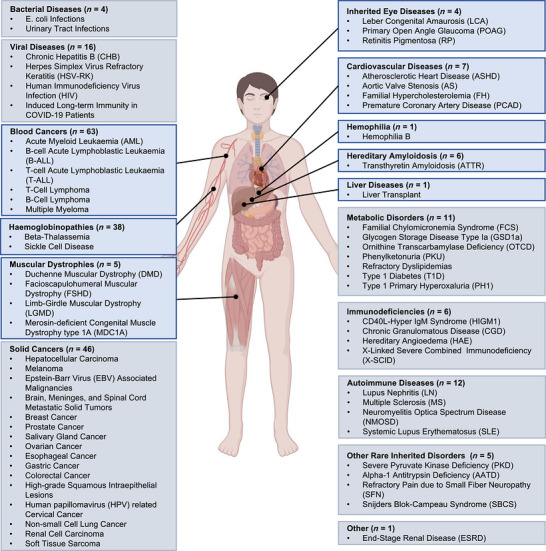
Clinical applications of genome editing technologies across human diseases. This figure summarizes the spectrum of human diseases currently being evaluated in genome‐editing‐based clinical trials, categorized by therapeutic area, including bacterial and viral infections, blood cancers, haemoglobinopathies, muscular dystrophies, solid tumors, inherited eye diseases, cardiovascular disorders, hemophilia, hereditary amyloidosis, liver diseases, metabolic disorders, immunodeficiencies, autoimmune diseases, and other rare inherited diseases. The value of “*n*” denotes the number of clinical therapeutic trials within each category. Data were sourced from publicly available records (https://crisprmedicinenews.com/clinical‐trials/) as of September 22, 2025, comprising a total of 226 trials. Only interventional therapeutic studies were included; observational studies, mechanistic investigations, and follow‐up trials were systematically excluded from the trial counts in each category.

Soon after the establishment of multiple gene‐editing platforms including CRISPR–Cas9, TALEN, and ZFN, therapeutic research based on these technologies began to grow rapidly. Numerous studies have demonstrated the feasibility of applying gene editing to treat a broad spectrum of human diseases, encompassing diverse categories such as blood cancers, hemoglobinopathies, muscular dystrophies, solid cancers, inherited eye diseases, and metabolic disorders (Figure 5 and Table 3). However, successful clinical translation of therapeutic genome editing is achieved only when delivery systems are precisely aligned with the anatomical and molecular context of the target disease. This fundamental principle is clearly articulated in the review by Cetin et al., who emphasize that delivery strategy, whether ex vivo electroporation for hematopoietic cells or in vivo LNP formulations for hepatocytes, serves as the primary determinant of therapeutic success across disease contexts. From the first approved ex vivo therapy, Casgevy, to emerging in vivo candidates targeting the liver, eye, and muscle, the expanding repertoire of delivery platforms underscores that aligning the molecular tool to the biological context of the target tissue is not merely a technical optimization, but a prerequisite for translating genome editing potential into meaningful clinical benefit [456].

**TABLE 3:** Clinical trials of genome editing in the treatment of human diseases.

Therapeutic areas	Clinical trials ID	Editor	Genes	Delivery method	Status	Sponsor organization
Blood cancers	ISRCTN14430213	Base editor	Anti‐CD33 CAR	Lentivirus	Phase 1	Great Ormond Street Hospital for Children NHS Foundation Trust
	NCT04849910	CRISPR–Cas9	CD33	Undisclosed	Phase 1/2	Vor Biopharma
	NCT03232619	CRISPR–Cas9	TRAC, anti‐CD19 CAR	Nonviral	Completed	Bioray Laboratories—Bangyao Biotechnology Co., Ltd.
	NCT03666000	Meganucleases	TRAC, anti‐CD19 CAR	Undisclosed	Phase 1	Imugene Limited
	NCT04213469	CRISPR–Cas9	PD‐1, anti‐CD19 CAR	Electroporation	Completed	Bioray Laboratories—Bangyao Biotechnology Co., Ltd.
	NCT04649112	Meganucleases	TRAC, anti‐CD19 CAR	Undisclosed	Phase 1	Precision BioSciences Inc.
	NCT05169489	MegaTAL	CBLB	Transfection with mRNA	Phase 1/2	Regeneron Pharmaceuticals
	NCT05631912	CRISPR–Cas9	TRAC	Undisclosed	Phase 1/2	Chinese PLA General Hospital
	NCT06014073	CRISPR–Cas9	TRAC, Power3	Undisclosed	Phase 1/2	Chinese PLA General Hospital
	NCT06321289	CRISPR–Cas9	TRAC, HLA‐A/B, CIITA, PD‐1, STAR	Adeno‐associated virus	Phase 1/2	Chinese PLA General Hospital
	NCT04026100	CRISPR–Cas9	TRAC, CD52	Lentivirus and electroporation	Phase 1	The First Affiliated Hospital of Nanjing Medical University
	NCT04023071	Undisclosed	CD16	Undisclosed	Completed	Fate Therapeutics
	NCT06500273	TALENs	TRAC, CD52	Lentivirus and electroporation	Phase 2	Allogene Therapeutics
	NCT06541405	AccuBase base editor	PD‐1, CD16	Undisclosed	Phase 1	Base Therapeutics (Shanghai) Co., Ltd.
	NCT02808442	TALENs	TCRα, CD52	Lentivirus and electroporation	Completed	Institut de Recherches Internationales Servier
	NCT06481735	CRISPR–Cas9	Power3	Lentivirus	Phase 1/2	Chinese PLA General Hospital
	NCT03190278	TALENs	TRAC, CD52	Lentivirus and electroporation	Phase 1	Cellectis S.A.
	NCT05662904	CRISPR–Cas9	CD33	Undisclosed	Phase 1	German Cancer Research Center
	NCT05949125	CRISPR–Cas9	Anti‐CD123–CAR, GHVD genes, Immune rejection genes	Undisclosed	Phase 1	AvenCell Europe GmbH
	NCT07026942	CRISPR–Cas9	Anti‐CD33–CAR	Adeno‐associated viral vector 6	Phase 1/2	Nationwide Children's Hospital
	NCT05984199	CRISPR–Cas9	CD33	Undisclosed	Phase 1/2	Vor Biopharma
	NCT06128044	CRISPR–Cas12a	PD‐1, B2M, B2M–HLA‐E fusion gene	Undisclosed	Phase 1	Caribou Biosciences, Inc.
	NCT06541444	AccuBase base editor	PD‐1, CD16	Undisclosed	Phase 1	Base Therapeutics (Shanghai) Co., Ltd.
	NCT04227015	CRISPR–Cas9	TRAC, CD52	Lentivirus	Phase 1	Zhejiang University
	2019‐003462‐40	CRISPR–Cas	CAR19, TCRαβ	Lentivirus and electroporation	Phase 1	Great Ormond Street Hospital for Children NHS Trust
	NCT02746952	TALENs	TCRα, CD52	Lentivirus and electroporation	Completed	Institut de Recherches Internationales Servier
	NCT04150497	TALENs	TRAC, CD52	Lentivirus and electroporation	Phase 1	Cellectis S.A.
	NCT04154709	CRISPR–Cas9	TRAC, CD19/CD22‐targeting CAR	Lentivirus and electroporation	Phase 1	Xuzhou Medical University
	NCT04557436	CRISPR–Cas9	CD52, TCRαβ	Lentivirus	Phase 1	Great Ormond Street Hospital for Children NHS Foundation Trust
	NCT05381181	Undisclosed	Anti‐CD19–UCAR	Undisclosed	Phase 1	Bioray Laboratories
	NCT04245722	Undisclosed	Anti‐CD19 CAR	Undisclosed	Phase 1	Fate Therapeutics
	NCT06014762	Cas‐CLOVER	TCRβ, B2M	PiggyBac	Phase 1	Poseida Therapeutics, Inc.
	NCT03166878	CRISPR–Cas9	TCR, B2M	Lentivirus and electroporation	Phase 1/2	Chinese PLA General Hospital
	NCT03298828	CRISPR–Cas9	PD‐1	Undisclosed	Phase 1	Third Military Medical University
	NCT03398967	CRISPR–Cas9	Anti‐CD19/CD20 CAR or anti‐CD19/CD22 CAR	Viral	Phase 1/2	Chinese PLA General Hospital
	NCT04037566	CRISPR–Cas9	MAP4K1	Lentivirus and electroporation	Phase 1	Xijing Hospital, The Fourth Military Medical University
	NCT04629729	CRISPR–Cas9	Anti‐CD19 CAR, TRAC	Adeno‐associated virus and electroporation	Phase 1	Fate Therapeutics
	NCT05336409	CRISPR	B2M, CIITA, HLA‐E, anti‐CD19 CAR	Undisclosed	Phase 1	Century Therapeutics, Inc.
	NCT05643742	CRISPR–Cas9	Regnase‐1, TGFBR2, TRAC, B2M, CD70, anti‐CD19 CAR	Adeno‐associated virus	Phase 1/2	CRISPR Therapeutics AG
	NCT04637763	CRISPR hybrid RNA‐DNA	TRAC, PD‐1, anti‐CD19 CAR	Adeno‐associated virus	Phase 1	Caribou Biosciences, Inc.
	NCT05741359	CRISPR–Cas9	Immune checkpoint genes	Electroporation	Phase 1	Bioray Laboratories
	NCT06838832	Undisclosed	Anti‐CD19 CAR	Undisclosed	Phase 1/2	Chinese PLA General Hospital
	NCT04767308	CRISPR–Cas9	CD5	Undisclosed	Phase 1	Huazhong University of Science and Technology
	NCT05714345	TALENs	TCR, CD52 Anti‐CD19 CAR	Lentivirus	Phase 2	Allogene Therapeutics
	NCT04416984	TALENs	TRAC, CD52	Lentivirus and electroporation	Phase 1/2	Allogene Therapeutics
	NCT04030195	Meganucleases	TRAC, Anti‐CD20 CAR	Adeno‐associated virus/lipid nanoparticles	completed	Precision BioSciences, Inc.
	NCT04093596	TALENs	TRAC, CD52	Lentivirus and electroporation	Phase 1	Allogene Therapeutics
	NCT04142619	TALENs	SLAMF7	Lentivirus and electroporation	Phase 1	Cellectis S.A.
	NCT05182073	CRISPR–Cas9	Anti‐BCMA CAR, CD38	Undisclosed	Phase 1	Fate Therapeutics Inc.
	NCT05308875	Undisclosed	PD‐1	Undisclosed	Not yet recruiting	Bioray Laboratories
	NCT05722418	Cas12a chRDNA	Anti‐BCMA CAR, TRAC, B2M	Adeno‐associated virus	Phase 1	Caribou Biosciences
	NCT03939026	TALENs	TCRα, CD52	Lentivirus and electroporation	Phase 1	Allogene Therapeutics
	NCT04984356	CRISPR–Cas9	CD7, TRAC	Electroporation	Completed	Wugen inc.
	NCT06492304	CRISPR–Cas9	TRAC, B2M, CD70, TGFBR2, Regnase‐1, CD70‐directed CAR	Undisclosed	Phase 1/2	CRISPR Therapeutics
	NCT06934382	Base editor	CD7, TRAC, PD‐1, CD52	Electroporation	Phase 1	Stephan Grupp MD PhD, Children's Hospital of Philadelphia
	ISRCTN15323014	CRISPR–Cas9	TRBC1, TRBC2, CD7, CD52	Lentivirus and electroporation	Phase 1	Great Ormond Street Hospital
	NCT03690011	CRISPR–Cas9	CD7	Electroporation	Phase 1	Baylor College of Medicine
	NCT05397184	Base editor	CD7	Undisclosed	Phase 1	Great Ormond Street Hospital for Children NHS Foundation Trust
	NCT05885464	Base editor	TRAC, CD7 UCAR, PD‐1, CD52	Electroporation	Phase 1/2	Beam Therapeutics Inc.
	NCT06514794	CRISPR–Cas9	CD7, TRAC	Electroporation	Phase 2	Wugen, Inc.
	NCT04264078	CRISPR–Cas9	CD7, TRAC	Undisclosed	Phase 1	Xinqiao Hospital of Chongqing
	NCT05607420	TALENs	TRAC, CD52	Lentivirus	Phase 1/2	Cellectis S.A.
	NCT05950334	Undisclosed	CAR, CD16, IL15‐RF, ADR, CD38	Undisclosed	Phase 1	Fate Therapeutics
Haemoglobinopathies	NCT06685536	Base editor	HBG promoter	Undisclosed	Enrolling by invitation	CorrectSequence Therapeutics Co., Ltd
	NCT06717932	Base editor	HBG promoter	Undisclosed	Enrolling by invitation	CorrectSequence Therapeutics Co., Ltd
	NCT03655678	CRISPR–Cas9	BCL11A	Electroporation	Phase 2/3	Vertex Pharmaceuticals Inc.
	NCT03728322	CRISPR–Cas9	HBB	Undisclosed	Phase 1	Allife Medical Science and Technology Co., Ltd
	NCT04211480	CRISPR–Cas9	HBG1	Electroporation	Completed	Bioray Laboratories—Bangyao Biotechnology Co., Ltd.
	NCT06024876	Base editor	HBG promoter	Undisclosed	Phase 1	CorrectSequence Therapeutics Co., Ltd
	NCT06328764	Base editor	HBG promoter	Undisclosed	Phase 1	CorrectSequence Therapeutics Co., Ltd
	NCT07000318	Base editor	HBG promoter	Undisclosed	Phase 1	Children's Hospital of Fudan University
	2018‐001320‐19	CRISPR–Cas9	BCL11A	Electroporation	Phase 1/2/3	Vertex Pharmaceuticals Incorporated
	NCT03653247	ZFNs	BCL11A	Nonviral	Phase 1/2	Sangamo Therapeutics
	NCT03745287	CRISPR–Cas9	BCL11A	Electroporation	Phase 1/2/3	Vertex Pharmaceuticals
	NCT04443907	CRISPR–Cas9	BCL11A	Undisclosed	Phase 1/2	Novartis Pharmaceuticals
	NCT04774536	CRISPR–Cas9	HBB	Electroporation	Phase 1/2	Mark Walters, MD
	NCT04819841	CRISPR–Cas	HBB	Undisclosed	Phase 1/2	Kamau Therapeutics
	NCT04853576	CRISPR–Cas12a	HBG1/2 promoter	Electroporation	Phase 1/2	Editas Medicine, Inc.
	NCT05329649	CRISPR–Cas9	BCL11A	Electroporation	Phase 3	Vertex Pharmaceuticals Incorporated
	NCT05456880	Base editor	HBG1, HBG2	Electroporation	Phase 1/2	Beam Therapeutics
	NCT05951205	CRISPR–Cas9	BCL11A	Electroporation	Phase 3	Vertex Pharmaceuticals Incorporated
	NCT06287086	CRISPR–Cas9	BCL11A	Undisclosed	Not yet recruiting	Bioray Laboratories
	NCT06287099	CRISPR–Cas9	BCL11A	Undisclosed	Not yet recruiting	Bioray Laboratories
	NCT06300723	CRISPR–Cas9	BCL11A	Undisclosed	Enrolling by invitation	Bioray Laboratories
	NCT06506461	CRISPR–Cas9	HBG‐115	Electroporation	Phase 1	St. Jude Children's Research Hospital
	NCT06565026	Base editor	HBG promoter	Undisclosed	Phase 1	CorrectSequence Therapeutics Co., Ltd
	NCT05145062	ZFNs	Enhancer of the BCL11A gene	Electroporation	Enrolling by invitation	Sangamo Therapeutics
	NCT05477563	CRISPR–Cas9	BCL11A	Electroporation	Phase 3	Vertex Pharmaceuticals Incorporated
	ChiCTR2100052858	CRISPR–Cas9	HBG1, HBG2	Undisclosed	Phase 1	923rd Hospital of The People's Liberation Army
	ChiCTR2100053406	CRISPR–Cas9	HGB1, HGB2	Undisclosed	Phase 1	The First Affiliated Hospital, Guangxi Medical University
	NCT04925206	CRISPR–Cas9	BCL11A	Undisclosed	Phase 1	EdiGene (GuangZhou) Inc.
	NCT03432364	ZFNs	BCL11A	Adeno‐associated virus and electroporation	Phase 1/2	Sangamo Therapeutics, Inc.
	NCT04390971	CRISPR/Cas9	Enhancer of the BCL11A gene	Undisclosed	Active not recruiting	Institute of Hematology and Blood Diseases Hospital
	NCT05015920	CRISPR–Cas9	HBB	Lentivirus	Completed	Shanghai BDgene Co., Ltd.
	NCT05356195	CRISPR–Cas9	BCL11A	Electroporation	Phase 3	Vertex Pharmaceuticals
	NCT05444894	CRISPR–Cas12α	HBG1/2 promoter	Electroporation	Phase 1/2	Editas Medicine
	NCT05577312	CRISPR–Cas9	Enhancer of the BCL11A gene	Undisclosed	Phase 1	Bioray Laboratories
	NCT06041620	CRISPR–Cas12b	HbF	Undisclosed	Active recruiting	Institute of Hematology & Blood Diseases Hospital, China
	NCT06065189	Base editor	HBG promoter	Undisclosed	Phase 1	Children's Hospital of Fudan University
	NCT06291961	Base editor	HBG promoter	Undisclosed	Phase 1	CorrectSequence Therapeutics Co., Ltd
	NCT06298630	CRISPR–Cas9	BCL11A	Undisclosed	Not yet recruiting	Bioray Laboratories
Solid cancers	NCT04417764	CRISPR–Cas9	PD‐1	Electroporation	Phase 1	Central South University
	2023‐510417‐25‐00	CRISPR–Cas9	PD‐1	Electroporation	Phase 1	Herlev Hospital
	NCT06912152	CRISPR–Cas	Anti‐B7‐H3 CAR	Undisclosed	Phase 1	Zhejiang University
	NCT07004647	CRISPR–Cas	Anti‐B7‐H3 CAR	Undisclosed	Active recruiting	Ruijin Hospital
	NCT03044743	CRISPR–Cas9	PD‐1	Electroporation	Phase 1/2	The Affiliated Nanjing Drum Tower Hospital of Nanjing University Medical School
	NCT06742593	CRISPR–Cas	Anti‐B7‐H3 CAR	Undisclosed	Phase 1	Suzhou Maximum Bio‐tech Co., Ltd.
	NCT05812326	Undisclosed	PD‐1	Undisclosed	Completed	Sun Yat‐Sen Memorial Hospital of Sun Yat‐Sen University
	NCT06895811	Undisclosed	Anti‐PSMA UCAR	Undisclosed	Phase 1	Shanghai Changzheng Hospital
	NCT04768608	CRISPR–Cas9	PD‐1	Electroporation	Phase 1	Zhejiang University
	NCT06228404	CRISPR–Cas9	Anti‐PSMA CAR	Electroporation	Phase 1	Shanghai Changzheng Hospital
	NCT04249947	PiggyBac transposon gene insertion platform	Anti‐PSMA CAR	PiggyBac DNA delivery system	Phase 1	Poseida Therapeutics, Inc.
	NCT05617755	CRISPR–Cas9	A large synthetic double‐stranded DNA cassette	Electroporation	Phase 1	Arsenal Biosciences, Inc.
	NCT03081715	CRISPR–Cas9	PD‐1	Undisclosed	completed	Hangzhou Cancer Hospital
	NCT07166263	AccuBase base editor	PD‐1, CD16	Undisclosed	Phase 1	Base Therapeutics (Shanghai) Co., Ltd.
	NCT06098898	AccuBase base editor	PD‐1, CD16	Undisclosed	Phase 1	Base Therapeutics (Shanghai) Co., Ltd.
	NCT04426669	CRISPR–Cas9	CISH	Undisclosed	Phase 1/2	Intima Bioscience, Inc.
	NCT07170254	CRISPR–Cas9	Undisclosed	Undisclosed	Active recruiting	Shanghai BDgene Co., Ltd.
	NCT02800369	ZFNs	HPV16, HPV18 E7	p‐DNA direct delivery	Phase 1	Huazhong University of Science and Technology
	NCT03057912	CRISPR–Cas9 and TALEN	HPV16, HPV18 oncogenes E6 and E7	p‐DNA	Phase 1	First Affiliated Hospital, Sun Yat‐Sen University
	NCT03226470	TALENs	HPV16 E6 and E7	Nonviral	Phase 1	Huazhong University of Science and Technology
	NCT06783270	CRISPR–Cas9	PD‐1	Electroporation	Phase 1	Inge Marie Svane
	NCT02793856	CRISPR–Cas9	PD‐1	Electroporation	Completed	Sichuan University
	NCT03525782	CRISPR–Cas9	PD‐1	Retroviral transduction	Phase 1/2	First Affiliated Hospital of Guangdong Pharmaceutical University
	NCT05566223	CRISPR–Cas9	CISH	Undisclosed	Phase 1/2	Intima Bioscience, Inc.
	NCT06097962	AccuBase base editor	PD‐1, CD16	Undisclosed	Phase 1	Base Therapeutics (Shanghai) Co., Ltd.
	NCT06846424	CRISPR–Cas9	Immunosuppressive‐related genes	Undisclosed	Phase 1	Shanghai Gynecologic Oncology Group
	NCT07067255	CRISPR–Cas9	CD146, anti‐GPC3–CAR	Undisclosed	Phase 1/2	Essen Biotech
	NCT06726564	CRISPR–Cas	Anti‐B7‐H3 CAR	Undisclosed	Phase 1	Suzhou Maximum Bio‐tech Co., Ltd.
	NCT03525652	CRISPR–Cas9	PD‐1	Undisclosed	Phase 1/2	The First Affiliated Hospital of Guangdong Pharmaceutical University
	NCT06815029	CRISPR	TGFβR2	Undisclosed	Phase 1	City of Hope Medical Center
	NCT06737146	CRISPR–Cas	Anti‐B7‐H3 CAR	Undisclosed	Phase 1	Suzhou Maximum Bio‐tech Co., Ltd.
	NCT05795595	CRISPR–Cas9	Regnase‐1, TGFBR2, TRAC, B2M, CD70, anti‐CD70 CAR	Adeno‐associated virus	Phase 1/2	CRISPR Therapeutics AG
	NCT04696731	TALENs	CD52, TRAC, anti‐CD70 CAR	Lentivirus and electroporation	Phase 1	Allogene Therapeutics, Inc.
	NCT06245915	CITE (CRISPR Integration of Transgene)	A large synthetic double‐stranded DNA cassette	Electroporation	Phase 1/2	Arsenal Biosciences, Inc.
	NCT06117878	AccuBase base editor	PD‐1, CD16	Undisclosed	Phase 1	Base Therapeutics (Shanghai) Co., Ltd.
	NCT03545815	CRISPR–Cas9	PD‐1, TCR	Lentivirus and electroporation	Phase 1	Chinese PLA General Hospital
	NCT03747965	CRISPR–Cas9	PD‐1	Lentivirus	Phase 1	Chinese PLA General Hospital
	NCT04976218	CRISPR–Cas9	TGFβR	Undisclosed	Phase 1	Chinese PLA General Hospital
	NCT03970382	CRISPR–Cas9	TRAC, TRBC, neoTCR	Nonviral	Phase 1	PACT Pharma, Inc.
	NCT04842812	CRISPR–Cas9	PD‐1	Electroporation	Phase 1	Second Affiliated Hospital of Guangzhou Medical University
	NCT05239143	Cas‐CLOVER	MHC‐I, TCR	PiggyBac	Phase 1/2	Poseida Therapeutics
	NCT05361174	TALENs	PD‐1	Undisclosed	Phase 1/2	Iovance Biotherapeutics, Inc.
	NCT05395052	CRISPR	CD38	Undisclosed	Phase 1	Fate Therapeutics
	NCT06237881	CRISPR–Cas9	SOCS1	Undisclosed	Phase 1/2	M.D. Anderson Cancer Center
	NCT06241456	CRISPR–Cas	TRAC, IL7RF, CD16a, CXCR2, TGF‐B, CD38, HER2	Undisclosed	Phase 1	Fate Therapeutics
	NCT06598371	CRISPR–Cas9	Regnase‐1, SOCS1	Electroporation	Phase 1/2	M.D. Anderson Cancer Center
Viral diseases	NCT06671093	Epigenetic editing	Hepatitis B viral genome	Lipid nanoparticles	Phase 1	Tune Therapeutics, Inc.
	NCT06680232	ARCUS genome editing	Hepatitis B virus genome	Lipid nanoparticles	Phase 1	Precision BioSciences, Inc.
	NCT04560790	CRISPR–Cas9	UL8, UL29	mRNA transfection	Completed	Shanghai BDgene Co., Ltd.
	NCT06474416	CRISPR–Cas9	HSV‐1	Lentivirus‐like particle	Phase 1	Shanghai BDgene Co., Ltd.
	NCT06474442	CRISPR–Cas9	HSV‐1	Lentivirus‐like particle	Phase 2	Shanghai BDgene Co., Ltd.
	NCT00842634	ZFNs	CCR5	Adenovirus	Completed	University of Pennsylvania
	NCT01044654	ZFNs	CCR5	Adenovirus	Completed	Sangamo Therapeutics Inc.
	NCT01252641	ZFNs	CCR5	Adenovirus	Phase 1/2, completed	Sangamo Therapeutics Inc.
	NCT02225665	ZFNs	CCR5	mRNA transfection	Phase 1/2, completed	Sangamo BioSciences, Inc.
	NCT02388594	ZFNs	CCR5	mRNA electroporated	Phase 1 completed	University of Pennsylvania
	NCT02500849	ZFNs	CCR5	Electroporation	Phase 1, completed	City of Hope Medical Center
	NCT03164135	CRISPR–Cas9	CCR5	Nonviral	Unknown	Affiliated Hospital to Academy of Military Medical Sciences
	NCT03617198	ZFNs	CCR5	Adeno‐associated virus	Phase 1	University of Pennsylvania
	NCT03666871	ZFNs	CCR5	Adenovirus	Phase 1/2	University of Cincinnati
	NCT05144386	CRISPR–Cas9	Three undisclosed genomic sites in the HIV DNA	AAV9	Phase 1	Excision BioTherapeutics
	NCT04990557	CRISPR–Cas9	PD‐1, ACE2	Undisclosed	Phase 1/2	Mahmoud Ramadan mohamed Elkazzaz
Metabolic disorders	NCT07176923	Base editor	APOC3	Lipid nanoparticles	Phase 1	CorrectSequence Therapeutics Co., Ltd
	NCT06735755	Adenine base editor	G6PC	Lipid nanoparticles	Phase 1/2	Beam Therapeutics Inc.
	NCT06255782	ARCUS nuclease	OTC	Adeno‐associated virus	Phase 1/2	IECURE, Inc.
	NCT05222178	Nuclease‐free gene‐editing	PAH	Adeno‐associated virus	Phase 1	Homology Medicines
	NCT06839235	CRISPR–Cas12i2	HAO1	Lipid nanoparticles	Phase 1/2	Arbor Biotechnologies
	ACTRN12623000809639	CRISPR–Cas9	ANGPTL3	Lipid‐based nanoparticle	Phase 1	CRISPR Therapeutics AG
	NCT05210530	CRISPR–Cas9	Undisclosed	Undisclosed	Phase 1	CRISPR Therapeutics
	NCT05565248	CRISPR–Cas9	B2M, TXNIP, PD‐L1, HLA‐E, TNFAIP3, MANF	Undisclosed	Phase 1/2	CRISPR Therapeutics AG
	NCT06239636	CRISPR–Cas12b	HLA‐I, HLA‐II, CD47	Lentivirus	Phase 1	Per‐Ola Carlsson
	NCT06511349	CRISPR–Cas12	AGXT	Lipid nanoparticles	Phase 1	RenJi Hospital
	NCT06892301	CRISPR–Cas12	AGXT	Lipid nanoparticles	Phase 1	Guangzhou Women and Children's Medical Center
Autoimmune diseases	NCT06681337	CRISPR–Cas9	Anti‐BCMA CAR, anti‐CD19 CAR, GHVD genes, immune rejection genes	Electroporation	Phase 1	Bioray Laboratories
	NCT07008378	Cas–CLOVER	TRAC, MHC1, anti‐CD19 CAR	PiggyBac transposon	Phase 1	Genentech, Inc.
	NCT06633042	CRISPR	Universal BCMA‐CD19 CAR	Undisclosed	Phase 1	Bioray Laboratories
	NCT06925542	CRISPR–Cas9	Anti‐CD19 CAR	Adeno‐associated viral	Phase 1	CRISPR Therapeutics
	NCT06485232	CRISPR–Cas9	Anti‐BCMA CAR, anti‐CD19 CAR, GHVD genes, immune rejection genes	Electroporation	Phase 1	Xuanwu Hospital, Beijing
	NCT07083349	AccuBase base editor	PD‐1, CD16	Undisclosed	Not yet recruiting	Base Therapeutics (Shanghai) Co., Ltd.
	NCT05859997	CRISPR–Cas9	GHVD genes, immune rejection genes	Electroporation	Enrolling by invitation	Bioray Laboratories
	NCT05988216	CRISPR–Cas9	Anti‐CD19 CAR, GHVD genes, immune rejection genes	Electroporation	Active recruiting	Bioray Laboratories
	NCT06255028	CRISPR	B2M, CIITA, HLA‐E, anti‐CD19 CAR	Undisclosed	Phase 1	Century Therapeutics, Inc.
	NCT06308978	CRISPR–Cas9	Anti‐CD19 CAR, TRAC	Adeno‐associated virus and electroporation	Phase 1	Fate Therapeutics
	NCT06373991	CRISPR–Cas9	TRAC, Power3	Lentivirus	Phase 1	EdiGene Inc.
	NCT06752876	CRISPR–Cas9	TRAC, anti‐CD19 CAR, PD‐1	Adeno‐associated virus	Phase 1	Caribou Biosciences, Inc.
Hereditary amyloidosis	NCT06082050	Base editor	TTR	Lipid nanoparticles	Phase 1	YolTech Therapeutics
	NCT06672237	CRISPR–Cas9	TTR	Lipid nanoparticles	Phase 3	Intellia Therapeutics
	ChiCTR2400081216	CRISPR–Cas9	TTR	Lipid nanoparticles	Phase 1	AccurEdit Therapeutics
	NCT04601051	CRISPR–Cas9	TTR	Lipid nanoparticles	Phase 1	Intellia Therapeutics
	NCT06128629	CRISPR–Cas9	TTR	Lipid nanoparticles	Phase 3	Intellia Therapeutics
	NCT06539208	Base editor	TTR	Lipid nanoparticles	Phase 1/2	YolTech Therapeutics Co., Ltd
Inherited eye diseases	NCT03872479	CRISPR–cas9	CEP290	Adeno‐associated virus (AAV5)	Phase 1/2	Editas Medicine, Inc.
	NCT06465537	CRISPR–Cas9	MYOC	Virus‐like particle	Active recruiting	Shanghai BDgene Co., Ltd.
	NCT06952842	CRISPR–Cas9	RHO	Adeno‐associated virus 8 (AAV8)	Phase 1/2	Chigenovo Co., Ltd
	NCT05805007	CRISPR–Cas9	RHO	Recombinant adeno‐associated virus	Phase 1	Peking University Third Hospital
Cardiovascular disease	ACTRN12623001095651p	CRISPR–Cas9	LPA	Lipid‐based nanoparticle	Phase 1	CRISPR Therapeutics AG
	NCT06458010	Base editor	PCSK9	Lipid nanoparticles	Phase 1	YolTech Therapeutics
	NCT05398029	Base editor	PCSK9	Lipid nanoparticle	Phase 1	Verve Therapeutics, Inc.
	NCT06461702	Base editor	PCSK9	Lipid nanoparticle	Phase 1	YolTech Therapeutics
	NCT06164730	Base editor	PCSK9	Lipid nanoparticle	Phase 1	Verve Therapeutics, Inc.
	ChiCTR2400093099	CRISPR–Cas	PCSK9	Lipid nanoparticles	Completed	The First Affiliated Hospital of Bengbu Medical College
	NCT06451770	Base Editor	ANGPTL3	Lipid nanoparticles	Phase 1	Verve Therapeutics, Inc.
Bacterial diseases	NCT05277350	CRISPR–Cas	Undisclosed	Bacteriophage	Phase 1, completed	SNIPR Biome
	NCT06938867	CRISPR–Cas	Undisclosed	Bacteriophage	Phase 1/2	SNIPR Biome
	NCT04191148	CRISPR–Cas3	E.coli genome	crPhage cocktail	Phase 1, completed	Locus Biosciences
	NCT05488340	CRISPR–Cas3	E.coli genome	Undisclosed	Phase 2	Locus Biosciences
Immunodeficiencies	NCT06959771	Base editor	CD40L	Undisclosed	Phase 1/2	National Institute of Allergy and Infectious Diseases (NIAID)
	NCT06559176	Prime editing	NCF1	Electroporation	Phase 1/2	Prime Medicine
	NCT06325709	CRISPR–Cas9	CYBB	Lentivirus	Phase 1/2	National Institute of Allergy and Infectious Diseases (NIAID)
	NCT05120830	CRISPR–Cas9	KLKB1	Lipid nanoparticles	Phase 1/2	Intellia Therapeutics
	NCT06634420	CRISPR–Cas9	KLKB1	Lipid nanoparticles	Phase 3	Intellia Therapeutics
	NCT06851767	Base editor	IL2RG	Undisclosed	Phase 1/2	National Institute of Allergy and Infectious Diseases (NIAID)
Haemophilia	NCT06611436	CRISPR–Cas9	FIX	Adeno‐associated virus	Phase 1/2	Be Biopharma
Liver diseases	NCT07053488	CRISPR–Cas9	HLA‐A, HLA‐B, CIITA	Through the perfusion circuit	Phase 1/2	AMERICAN ORGAN TRANSPLANT AND CANCER RESEARCH INSTITUTE LLC
Muscular dystrophy	NCT05514249	CRISPR–Cas9	Dystrophin	Recombinant adeno‐associated virus	Phase 1	Cure Rare Disease, Inc
	NCT06594094	CRISPR/hfCas12Max	DMD exon 51	Adeno‐associated virus	Not yet recruiting/recruiting	HuidaGene Therapeutics Co., Ltd.
	NCT06907875	Epigenome editing	DUX4	Adeno‐associated viral vector, serotype rh74 (AAVrh74)	Phase 1/2	Epicrispr Biotechnologies
	NCT05588401	CRISPR–Cas	Undisclosed	Undisclosed	Phase 1/2	Simone Spuler, MD
	NCT06582537	CRISPR–Cas9	LAMA2	Undisclosed	Completed	Maastricht University
Other rare inherited diseases	ChiCTR2300073795	CRISPR	Red‐cell type pyruvate kinase gene	Adeno‐associated virus 6 (AAV6)	Phase 1	Shanghai Children's Medical Center
	NCT06389877	Base editor	SERPINA1	Lipid nanoparticle	Phase 1/2	Beam Therapeutics Inc.
	NCT06622668	CRISPR–Cas9	SERPINA1	Lipid nanoparticle	Phase 1/2	Intellia Therapeutics
	NCT06980948	ZFNs	SCN9A	Adeno‐associated virus	Phase 1	Sangamo Therapeutics
	NCT06860672	Base editor	CHD3	Dual adeno‐associated virus	Phase 1	Yongguo Yu
Other	NCT07053462	CRISPR–Cas9	HLA‐A, HLA‐B, CIITA	Undisclosed	Phase 1/2	AMERICAN ORGAN TRANSPLANT AND CANCER RESEARCH INSTITUTE LLC

Abbreviation: CRISPR, clustered regularly interspaced short palindromic repeats.

Clinical trials data (https://crisprmedicinenews.com/clinical‐trials/) was accessed on September 22, 2025, trials not including interventions using genome editors were excluded.

The biological context dictates the chemistry, tropism, and dosing strategy of the delivery system. In the liver, GalNAc conjugates and ionizable lipid formulations enable highly selective hepatocyte editing, whereas blood disorders favor ex vivo Cas9 RNPs electroporation into hematopoietic stem cells, avoiding systemic exposure. The immune‐privileged eye supports AAV‐mediated subretinal delivery, skeletal muscle depends on engineered capsids that enhance tropism while reducing hepatic uptake, and CNS disorders require barrier‐penetrant or locally infused AAV vectors, supported by evolved capsids and refined expression cassettes. Polymeric and stimuli‐responsive nanoparticles further extend genome editing to pulmonary, immune, and bone tissues by exploiting local physicochemical environments. Taken together, these cases illustrate that disease indication does more than define a therapeutic target—it fundamentally constrains and instructs delivery design. Matching the molecular tool to the anatomical and physiological context remains the decisive factor in transforming genome‐editing potential into patient benefit.

### 7.1 Liver‑Targeted In Vivo Genome Editing

**FIGURE 6:**
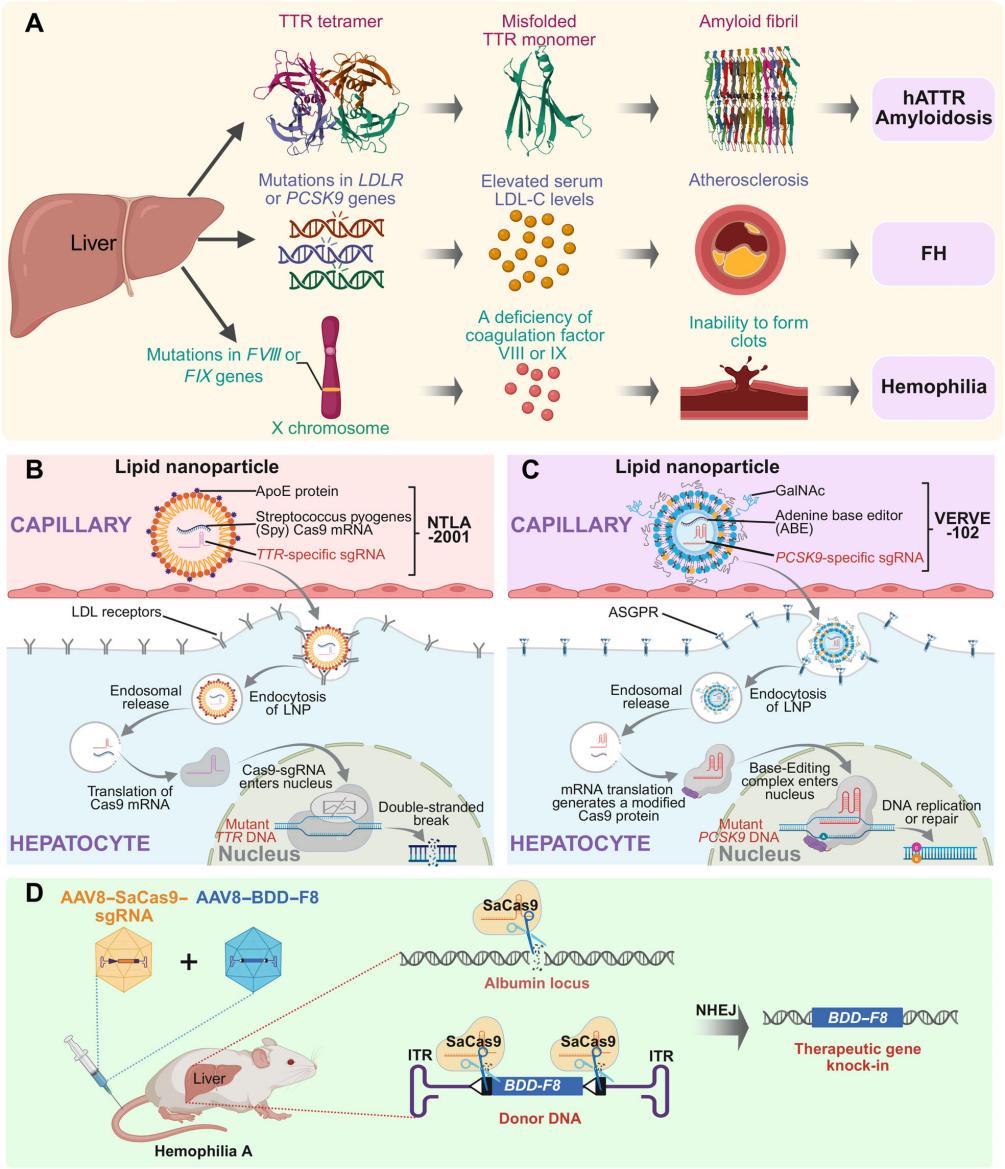
Delivery strategies of the clustered regularly interspaced short palindromic repeats (CRISPR)–Cas systems for monogenic liver diseases. (A) Disease mechanisms. In hereditary transthyretin amyloidosis (hATTR), misfolding of hepatic transthyretin (TTR) tetramers leads to amyloid fibril deposition in peripheral tissues. Loss‐ or gain‐of‐function mutations in *LDLR* or *PCSK9* elevate circulating LDL cholesterol, causing familial hypercholesterolemia (FH). X‐linked mutations in F8 or F9 result in *FVIII* or *FIX* deficiency, underlying hemophilia A or B, respectively. (B) Therapeutic workflow of *NTLA‐2001*. LNPs encapsulating SpyCas9 mRNA and *TTR*‐targeting sgRNA associate with ApoE in circulation and are internalized by hepatocytes via LDLR‐meditated endocytosis. Following endosomal escape, Cas9 is translated and forms a Cas9–sgRNA complex that enters the nucleus to induce DSBs at the TTR locus, resulting in gene knockout and durable TTR reduction. (C) Mechanism of action of *VERVE‐102*. GalNAc‐decorated LNPs delivering ABE mRNA and PCSK9–sgRNA engage the hepatocyte‐specific ASGPR for endocytosis. After endosomal escape and translation, the ABE–sgRNA complex traffics to the nucleus and installs a precise A‐to‐G transition in PCSK9, leading to long‐term PCSK9 inactivation. (D) Dual‐AAV strategy for hemophilia A. Two recombinant AAV8 vectors deliver SaCas9–sgRNA and a BDD‐F8 donor template. SaCas9 introduces a targeted break at the albumin locus, enabling NHEJ‐mediated knock‐in of the BDD‐F8 cassette and sustained hepatic expression of factor VIII. (Figure was created with BioRender.com.)

Clinical translation of genome editing has progressed rapidly in monogenic liver diseases such as hereditary transthyretin (ATTR) amyloidosis, hemophilia, and familial hypercholesterolemia (FH), where therapeutic benefit follows from targeting a single hepatic gene [457] (Figure 6A). These indications now benchmark in vivo delivery using ionizable LNPs, GalNAc‐functionalized formulations, and dual AAV vectors [9, 409, 458, 459, 460, 461, 462], collectively demonstrating remarkable clinical efficacy while highlighting persistent constraints related to tropism, redosing, and immunogenicity.

In ATTR amyloidosis, a disease driven by gain‐of‐function mutations in transthyretin (TTR) expressed primarily by hepatocytes, Intellia's NTLA‐2001 encapsulates *Sp*Cas9 mRNA and a TTR‐targeting sgRNA in a four‐component ionizable LNP optimized for ApoE‐mediated hepatocyte uptake (Figure 6B). In preclinical studies, a single 1 mg kg^−1^ intravenous dose in mice resulted in approximately 70% editing of the Ttr gene and a >97% reduction in serum TTR protein levels, which persisted for at least 12 months after a single administration [409]. Similarly durable and potent TTR knockdown (>94% reduction in serum TTR) was observed in cynomolgus monkeys (NHPs) following a single 3 mg kg^−1^ dose. Subsequently, in a Phase 1 clinical trial involving patients with hereditary transthyretin amyloidosis (hATTR), a single 0.3 mg kg^−1^ intravenous infusion of NTLA‐2001 led to a mean reduction of 87% in serum TTR protein concentration within 28 days. The treatment was well‐tolerated, with no serious adverse events reported [9, 463]. The transient, nonintegrating exposure and titratable dosing are primary safety advantages, though reliance on ApoE–LDLR constrains extrahepatic use and repeat dosing may be complicated by anti‐PEG antibodies.

FH therapies leverage BEs and ligand‐directed delivery to enhance hepatocyte specificity. Heterozygous FH, commonly caused by *PCSK9* gain‐of‐function variants, is well suited to gene inactivation. Verve Therapeutics’ VERVE‐101 delivers ABE mRNA and a *PCSK9* guide in a standard LNP; VERVE‐102 incorporates GalNAc ligands for ASGPR targeting (Figure 6C), achieving substantially higher liver delivery compared with untargeted LNPs [458, 459].

Preclinical studies in cynomolgus monkeys demonstrated that a single dose of VERVE‐101 at 1.5 mg kg^−1^ led to profound and durable suppression of serum PCSK9 protein by approximately 83–93% and reduced LDL cholesterol by up to 69% [15]. Subsequently, in the first‐in‐human Phase 1b heart‐1 trial, a single intravenous infusion of VERVE‐101 at 0.45 mg kg^−1^ produced LDL cholesterol reductions of up to 48% in participants, while the single participant dosed at 0.6 mg kg^−1^ achieved a 55% reduction that was sustained for at least 6 months [454, 458, 464]. Building upon these promising results, subsequent efforts have focused on VERVE‐102, which in NHP models demonstrated robust efficacy: a single infusion of VERVE‐102 (3 mg kg^−1^) yielded durable mean reductions of 80% in blood PCSK9 and 62% in LDL‐C [459]. Beyond PCSK9, GalNAc–LNPs have also targeted ANGPTL3, yielding a remarkable 96.3% reduction in plasma triglycerides in rodent models [465]. While GalNAc conjugation increases on‐target editing at lower lipid doses, receptor saturation and the immunogenicity of next‐generation lipid chemistries remain considerations for chronic treatment.

Unlike gene silencing strategies, hemophilia treatments require long‐term expression of missing coagulation factors. Recombinant AAV5 encoding factor IX (FIX) or factor VIII (FVIII) has reduced bleeding episodes and allowed discontinuation of prophylaxis; for example, a single infusion of AAV5–hFIX at a dose of 2 × 10^13^ vg kg^−1^ resulted in a 64% reduction in the annualized bleeding rate and enabled 96% of patients to discontinue routine prophylaxis [466, 467]. Similarly, the AAV5–hFVIII–SQ gene therapy, valoctocogene roxaparvovec (Roctavian), has demonstrated significant efficacy in clinical trials and has subsequently gained regulatory approval as a one‐time treatment for severe hemophilia A [468]. Yet the ∼4.7 kb AAV packaging limit constrains delivery of larger editors such as Cas9 or BEs [469]. Dual‐AAV designs address this by splitting effectors and guide/donor sequences across two capsids: split‐intein dual‐AAV systems have achieved ∼10–80% editing across liver, heart, and muscle, restoring dystrophin in Duchenne models and correcting hepatic alleles via HDR [119, 124, 470]. In hemophilia models, dual AAV8‐mediated in vivo genome editing has demonstrated therapeutic promise: HDR‐based correction of the endogenous FIX locus in hemophilia B mice enabled sustained supraphysiological FIX activity [461], while NHEJ‐mediated targeted integration of a BDD‐F8 transgene into the albumin locus achieved long‐term FVIII expression and phenotypic correction in hemophilia A mice [462] (Figure 6D). However, pre‐existing capsid immunity, redosing limitations, and prolonged nuclease expression remain major challenges.

Based on the aforementioned findings, the field of liver‐targeted genome editing has established a clear division of labor among distinct platforms, each with its defined technological trajectory. Ionizable LNPs, leveraging their transient activity and high delivery efficiency, have emerged as the modality of choice for achieving potent yet short‐lived gene knockout in the liver. GalNAc‐conjugated LNPs enhance hepatocyte‐specific delivery through targeted engagement of the asialoglycoprotein receptor, substantially improving the therapeutic window for metabolic disorders such as FH. Meanwhile, dual AAV systems, with their expanded cargo capacity, remain indispensable for therapeutic scenarios requiring gene addition or precise correction, such as the restoration of coagulation factors in hemophilia. Priorities ahead include enabling AAV redosing via less immunogenic capsids, expanding LNPs tropism beyond the liver, and using ligand chemistry to fine‐tune intrahepatic distribution—together charting a path to broader, repeatable liver gene‐editing therapy.

### 7.2 Ex Vivo HSC and Immune‑Cell Editing

Ex vivo genome editing of HSPCs and CAR‐T cells relies primarily on RNP electroporation and LV transduction. Electroporation introduces preassembled RNPs, ensuring defined stoichiometry and transient nuclease activity that reduce off‐target editing. In contrast, LV vectors enable durable transgene expression for short hairpin RNA (shRNA) or CAR constructs but carry risks of insertional mutagenesis and expansion due to random genomic integration [194].

**FIGURE 7:**
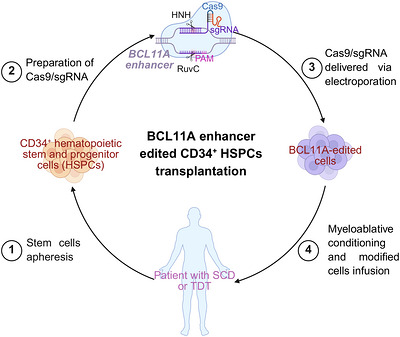
Schematic of CRISPR–Cas9 editing of the BCL11A enhancer for sickle cell disease and β‐thalassemia. The therapeutic process consists of four major stages: (1) Collection of autologous CD34^+^ hematopoietic stem and progenitor cells (HSPCs) via apheresis. (2) In vitro assembly of CRISPR–Cas9 RNP complexes targeting the erythroid‐specific enhancer of *BCL11A*. (3) Electroporation‐mediated delivery of the RNPs into HSPCs, enabling precise editing of the enhancer to derepress fetal hemoglobin (HbF) expression. (4) Myeloablative conditioning and reinfusion of the edited HSPCs to achieve durable hematopoietic reconstitution and therapeutic HbF induction. CRISPR, clustered regularly interspaced short palindromic repeats; SCD, sickle cell disease; TDT, transfusion‐dependent β‐thalassemia. (Figure was created with BioRender.com.)

In sickle‐cell disease (SCD) and transfusion‐dependent β‐thalassemia (TDT), RNP electroporation for editing the erythroid enhancer of BCL11A has successfully advanced from clinical trials to clinical application, with Casgevy (exa‐cel) becoming the first therapy of its kind to receive regulatory approval from agencies including the UK's MHRA and the US FDA [471, 472] (Figure 7). Patient‐derived CD34^+^ cells achieved approximately 80% on‐target allelic editing in preclinical studies, and HbF levels increased to about 40% within months posttreatment, sufficient for therapeutic benefit [8]. Edited human HSPCs maintain multilineage engraftment in xenografts without detectable recurrent off‐target lesions, confirming the genomic safety of transient RNP delivery. Beyond enhancer disruption, RNP electroporation supports HDR, base editing, and prime editing for HBB correction, achieving up to 80% allele repair while preserving engraftment [7, 473, 474, 475]. Because HDR protocols requiring donor DNA and extended culture can reduce stemness, BE and PE are increasingly preferred for scarless correction without DSBs. This paradigm shift from gene disruption to gene repair reflects the increasing demand for both safety and precision in clinical translation. Cetin and colleagues have systematically reviewed the evolution of this technological landscape, while the progression of multiple next‐generation gene editing platforms into clinical trials provides compelling evidence of its translational feasibility. In the context of HbF reactivation, EDIT‐301 employs AsCas12a to target the HBG1/2 promoters, disrupting BCL11A binding sites to restore γ‐globin expression. In contrast, BEAM‐101 utilizes an ABE to introduce an A→G transition within the same regulatory region, recapitulating benign mutations associated with hereditary persistence of HbF. This strategy effectively avoids DSBs, thereby minimizing the risks of indels and genomic rearrangements [456]. LV‐based *BCL11A* knockdown strategies through shRNA provide an alternative route to HbF reinduction [476]. Transduced patient CD34^+^ cells show durable HbF elevation, though insertional mutagenesis remains a long‐term risk. Self‐inactivating vectors and safer promoters mitigate this, but integration‐site tracking and quantitative long‐term clonal tracking remain essential.

Similar delivery paradigms extend to CAR‐T cell manufacturing. CRISPR RNPs disrupt endogenous *TRAC* or immune‐checkpoint loci, while LV or γ‐retroviral systems insert CAR constructs. Because CAR‐T products are short‐lived, insertional genotoxicity is less concerning, and cassette‐free RNP editing enables rapid, cGMP‐compatible manufacturing. Engraftment outcomes confirm the safety of ex vivo HSPC editing. In immunodeficient mice and early human studies, edited HSPCs show durable and multilineage reconstitution comparable to unedited controls. LV‐modified products also yield robust but occasionally display oligoclonal expansions, emphasizing the need for long‐term monitoring.

Advances in nuclease platforms and target selection continue to broaden therapeutic scope. CRISPR–Cas9‐mediated HDR enables precise *IL2RG* correction for X‐linked severe combined immunodeficiency, restoring immune function [477]. ZFN targeting the *AAVS1* locus in human CD34^+^ HSCs results in long‐term engraftment, with 6–16% of human cells in recipient bone marrow stably carrying the integrated transgene [478]. In primary human B cells, CRISPR–Cas9 mediates up to 94% gene disruption and high‐efficiency therapeutic *FIX* cassette insertion [479]. For HIV, CRISPR‐based knockout of *CCR5* and *CXCR4* confers viral resistance, though dual disruption reduces engraftment, illustrating trade‐offs between antiviral efficacy and cellular fitness [480]. Optimizing delivery enhances both efficiency and cell quality. Infusing CD34^+^ cells soon after electroporation improves survival [481]. Polymer‐based nanoparticles and the TRIAMF platform facilitate high‐efficiency, low‐toxicity RNP transfer while preserving multilineage colony‐forming capacity [482, 483]. In pyruvate kinase deficiency, TALEN or CRISPR systems achieve up to 96% precise genomic integration in progenitors, though maintaining yields after transplantation remains challenging [484].

In conclusion, RNP electroporation and LV transduction constitute the foundation of ex vivo platforms for hemoglobinopathies and CAR‐T engineering. RNPs provide precise, transient editing with minimal genotoxic risk, while LVs ensure stable expression but require integration surveillance. Further efforts should focus on shortening culture time, refining vector design, and integrating quantitative clonal analytics to enhance safety and durability in large‐scale clinical use.

### 7.3 Musculoskeletal and Neuromuscular Disorders

Musculoskeletal disorders exemplify both the promise and complexity of genome‐editing delivery platforms. DMD remains a model target, as the 11.3 kb *DMD* cDNA exceeds the 4.7 kb AAV limit. Microdystrophin AAVs, encoding essential spectrin‐like and cysteine‐rich domains, restore muscle membrane stability in animal models and are under clinical evaluation [485]. However, achieving durable expression requires high vector doses, heightening risks of NAbs against AAV capsid and dystrophin epitopes. These limitations have driven efforts toward CRISPR‐based strategies that enable endogenous gene repair with reduced immune exposure.

CRISPR‐mediated exon reframing has emerged as an alternative to microdystrophin replacement. Dual AAV systems encoding SpCas9 and sgRNA often use scAAV to bypass second‐strand synthesis, improving editing kinetics and dystrophin restoration in an exon‐deleted DMD mouse model, reaching therapeutic levels with markedly lower doses [119]. Nonetheless, integration risk and long‐term immunity remain intrinsic to AAV platforms. VLPs‐mediated editing introduces a transient, nonintegrating option with high efficiency. Fourth‐generation VLPs integrate chemical Gag dimerization, RNA packaging signals, and riboswitch‐controlled sgRNA release to enhance delivery [110]. Systemic administration in DMD mouse models enabled skeletal muscle‐specific gene editing, excising the mutated exon 4 in the Dmd gene to restore dystrophin expression and improve muscle function without detectable editing in nonmuscle tissues [486]. While transient exposure minimizes off‐target activity and immune priming, scalable manufacturing remains a key barrier to clinical translation.

Localized genome editing offers advantages for diseases such as osteoarthritis (OA), where avascular cartilage limits systemic delivery. Injectable, shear‐thinning hydrogels act as depot technologies for CRISPR reagents (Cas RNPs, LNPs, or VLPs), providing sustained release and joint confinement. pH‐responsive crosslinkers synchronize release with inflammatory flares, maintaining intra‐articular concentrations without systemic cytokine activation. These platforms can be combined with next‐generation compact nucleases (e.g., Cas12j, Cas13bt) to enhance cartilage penetration. Beyond depot systems, hydrogel‐cell hybrids enable localized and regenerative genome editing. CRISPR‐corrected patient‐derived myogenic progenitor cells encapsulated in hydrogel formed dystrophin‐positive myofibers with long‐term engraftment and neuromuscular junction formation [487]. In OA, a FOXO3‐targeted CRISPR hydrogel (FoxO3–NETT@SMs) enhanced mitophagy, reduced oxidative stress, and increased chondrocyte proliferation [488]. Similarly, hydrogels carrying CRISPR tools against CFIm25 or FGF18 promoted cartilage regeneration and suppressed inflammation via NF‐κB pathway modulation [354, 489]. Microfluidic fabrication and inert crosslinkers now allow precise control of degradation and release kinetics, improving reproducibility. CRISPR‐engineered chondrocytes encapsulated in hyaluronan hydrogels showed increased matrix deposition and reduced macrophage infiltration through TGF‐β‐activated kinase 1 knockout [490]. Hybrid antibacterial hydrogels embedding CRISPR‐modified bacteriophage have also shown efficacy in osteomyelitis models, disrupting Staphylococcus aureus biofilms [491].

Collectively, the evolution from microdystrophin AAVs to VLP‐ and hydrogel‐based CRISPR systems illustrates the diversification of genome‐editing modalities for musculoskeletal and neuromuscular therapy. Each platform balances trade‐offs between packaging capacity, exposure duration, scalability, and immune recognition. Comparative studies in large‐animal models with deep immune‐profiling and long‐term functional evaluation will be critical to determine optimal delivery systems or combinations for sustained, safe, and clinically translatable muscle and joint regeneration.

### 7.4 CNS Delivery

The CNS poses unique challenges for therapeutic genome editing in disorders such as SMA, epilepsy, and neurodegeneration. Overcoming the BBB, achieving cell‐type‐specificity, and maintaining safety after a single curative dose remain key obstacles. Four delivery platforms currently define the field: naturally occurring AAV serotype 9 (AAV9), its capsid‐evolved derivatives (e.g., PHP.B and rAAV.cc47), intrathecally administered LNPs, and cell‐penetrating‐peptide–RNP (CPP–RNP) complexes. Each balances efficiency, tropism, and redosing potential differently.

AAV‐based delivery remains the benchmark for durable CNS editing. AAV9, identified for its intrinsic ability to traverse the BBB, enables widespread neuronal and glial transduction at relatively low doses [492, 493, 494]. This feature supports efficient editing for broad disorders such as SMA while reducing hepatotoxicity and anticapsid immune responses. However, higher doses still induce complement activation and hepatic inflammation in pediatric SMA patients, emphasizing the need for improved CNS‐tropic capsids. Directed evolution produced variants such as PHP.B, which enhances neuronal transduction in mice through endothelial receptor binding and AAV.cc47, which extends tropism to the brain and heart in NHPs [19, 407]. These variants move toward translational readiness but remain constrained by pre‐existing immunity and concerns about sustained nuclease expression in long‐lived neurons.

LNPs‐based systems provide transient expression and redosing potential. While systemic LNPs primarily target the liver, intranasal administration offers a noninvasive route that bypasses the BBB and facilitates direct brain targeting [495]. Additionally, another recent study demonstrated that intracerebroventricular injection of acid‐degradable LNPs (ADP‐LNPs) encapsulating Cas9 mRNA and sgRNA efficiently transfected neural stem cells. These cells subsequently proliferated and distributed throughout the developing brain, achieving widespread gene editing [496]. The rapid clearance of mRNA and sgRNA minimizes off‐target activity and immunogenicity, though achieving uniform rostral‐caudal distribution and avoiding microglial activation remain unresolved.

CPP–RNP complexes advance transient genome editing by delivering preassembled RNP directly into CNS cells, eliminating the need for DNA vectors. Their nanoscale size favors diffusion and limited antigenicity, making them appealing for early‐life interventions requiring subsequent redosing. CPP‐mediated entry allows transient yet potent editing suitable for mouse brain neurons and astrocytes, with efficiency increasing dose‐dependently [497]. However, avoidance of proteasomal degradation remains a technical hurdle, and large animal data are still limited.

Comparative analyses reveal a continuum between platforms offering durable expression versus those emphasizing safety and flexibility for redosing. AAV9 and its engineered derivatives provide persistent expression suitable for monogenic loss‐of‐function diseases but are hindered by immunogenicity and redosing limits. Conversely, LNPs and CPP–RNPs prioritize transient editing with flexible dosing and lower immune risk, ideal for conditions needing reversible or localized modulation. Continued innovations in capsid design, lipid chemistry, and peptide engineering are converging toward delivery systems that achieve efficient, safe, and broadly accessible CNS genome editing.

The eye and inner ear pose substantial anatomical and physiological barriers to genome‑editing therapeutics. Advances in rAAV, VLPs, and EVs‐based delivery systems are beginning to overcome these challenges, enabling targeted editing of neuro‑sensory tissues such as the retina and cochlea. Representative platforms, such as sub‐retinal AAV for LCA, VLP for cone dystrophies, and exosome‐mediated cochlear transport, demonstrate both progress and persistent constraints in achieving precise, efficient delivery.

**FIGURE 8:**
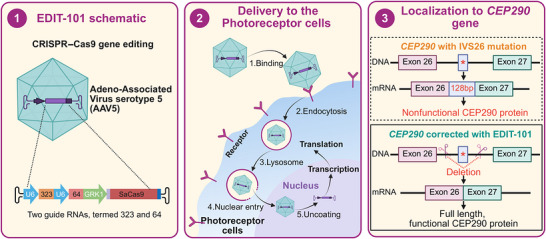
Graphical illustration of EDIT‐101‐mediated genome editing for CEP290‐associated photoreceptor degeneration. The mechanism of EDIT‐101 can be conceptualized in three functional modules: (1) Schematic representation of the EDIT‐101 construct packaged in an adeno‐associated virus serotype 5 (AAV5) vector. The cassette encodes SaCas9 nuclease and two guide RNAs (gRNA‐323 and gRNA‐64) that target the pathogenic IVS26 intronic region of the CEP290 gene. (2) Delivery process of the AAV5 vector to photoreceptor cells, including cellular binding, endocytosis, endosomal escape, nuclear entry, and subsequent transcription and translation of the clustered regularly interspaced short palindromic repeats (CRISPR) components. (3) Molecular outcome of the EDIT‐101 treatment. The IVS26 mutation in CEP290 disrupts normal splicing and produces a truncated, non‐functional protein. Dual‐gRNA‐directed cleavage removes the mutant intronic fragment, restoring proper mRNA splicing and enabling expression of full‐length, functional CEP290 protein. (Figure was created with BioRender.com.)

AAV remains the gold standard for ocular gene transfer, with sub‐retinal injection providing efficient photoreceptor and RPE transduction. EDIT‐101, an AAV5 vector carrying saCas9 and a sgRNA correcting the deep intronic *CEP290* mutation underlying most LCA10 cases (Figure 8), yielded robust, photoreceptor‐specific indels and restored light responses in preclinical models [164]. The AAV5 serotype exhibits strong neurotropism but limited inner‐retinal penetration, necessitating sub‐retinal rather than intravitreal delivery. Editing activity plateaued within weeks, minimizing prolonged nuclease exposure. However, pre‑existing antibodies and the 4.7 kb packaging limit constrain broader use. Capsid‐engineered variants such as AAV2‐derived variant 7m8, with a seven‑amino‑acid insertion that enhances photoreceptor tropism after intravitreal injection, may reduce surgical complexity but have yet to match sub‑retinal AAV5 in clinical efficiency [498]. In contrast to AAV's durable transgene persistence, VLP delivery provides a transient alternative suitable for dominantly inherited retinal diseases requiring allele disruption. Self‑assembling VLPs can encapsulate Cas9 RNPs or mRNAs, delivering high‑fidelity edits without viral DNA integration. In a laser‐induced mouse model of retinal vascular disease, sub‐retinal injection of Cas9–RNP‐loaded VLPs achieved a 43% reduction in the area of choroidal neovascularization by specifically editing the *Vegfa* gene in retinal pigment epithelial cells [109], establishing a benchmark for postmitotic retinal editing. VLPs circumvent AAV's size constraints and minimize off‑target exposure due to rapid disassembly, though large‐scale manufacturing and consistency in RNP loading remain limiting. Immune compatibility within the human retina also requires further validation.

The cochlea, enclosed by the blood–labyrinth barrier, demands noninvasive vectors capable of traversing the round‐window membrane. Engineered EVs enable efficient RNP delivery to auditory hair and supporting cells, producing precise genome edits and preserving hearing thresholds in a mouse model of dominant progressive hearing loss [499]. Their transient RNP payloads mitigate the risk of prolonged nuclease expression and off‐target effects. Yet, scalable production preserving vesicle integrity, consistent biodistribution, and editing efficiencies above the ∼30% functional threshold remain active challenges. Incorporating microfluidic electroporation for enhanced RNP encapsulation and real‐time tracking of vesicle kinetics may improve translational reliability. Allele disruption via CRISPR/Cas9 remains a leading approach for inherited retinopathies. Dual‐AAV excision of intronic CEP290 splice site restored normal transcripts in LCA10 models, while *GUCY2D* knockout in dominant cone‐rod dystrophy (CORD6) mice and macaques improved retinal structure and function without excessive immune responses [500, 501]. Despite encouraging efficacy, dual‐vector logistics complicate clinical translation. Base editing further refines precision by avoiding DSBs: ABEs delivered via split dual AAVs corrected a pathogenic RPE65 nonsense mutation, restoring transcript expression and light sensitivity in LCA models [502]. Although this strategy demands tight coordination between vectors, it highlights the feasibility of nucleotide‐level correction in postmitotic retinal tissue.

Taken together, sub‐retinal AAVs, transient VLPs, and EVs carriers collectively define a versatile toolkit for ocular and otic genome editing. AAVs provide high‐efficiency, durable transduction but are constrained by immunogenicity and size limits; VLPs deliver large RNP cargos transiently yet require improved yield and safety validation; and EVs offer minimally immunogenic passage across complex barriers though currently achieve modest editing rates. Continued progress in capsid design, protein‐shell engineering, and vesicle fabrication will accelerate translation of these modalities toward durable, safe, and precise correction of inherited blindness and deafness.

## 8 Future Directions and Opportunities

Therapeutic genome editing is entering a transformative phase shaped by the convergence of molecular engineering, synthetic chemistry, materials science, and computational design. Efficient, cell‐selective delivery of large and fragile editing macromolecules remains one of the field's central bottlenecks, one that increasingly demands multidisciplinary solutions rather than isolated innovation.

Chemical optimization now sits at the core of translational viability. Modifications such as triazole linkages, 2′‐O‑methyl or 2′‐fluoro ribose chemistries [503, 504], and structured 3′‐end motifs have transformed sgRNAs and prime‐editing guide RNAs (pegRNAs) from laboratory reagents into clinically robust components, enabling multifold gains in editing efficiency and stability within dosing limitations imposed by delivery. These refinements increasingly determine whether therapeutic activity can be achieved within dosing and formulation constraints. In parallel, advances in lipid chemistry have expanded the reach of LNP delivery. Systematic tuning of helper‐lipid composition, surface charge, and ligand presentation has enabled organ tropism beyond the liver, enabling lung‐targeting “SORT” LNPs and highly selective GalNAc‐decorated LNP that achieve highly selective, deep, and durable hepatic editing in both NHPs and patients. These results highlight the interplay between lipid composition, organ physiology, and pharmacokinetic behavior in shaping biodistribution and safety.

Viral‐vector engineering is undergoing a similar transformation. Directed evolution and computational design have produced AAV variants with cross‐species tropism, endothelial penetration, and retinal or CNS specificity. Pairing these capsids with regulatory elements such as miR‐142 or miR‐183 target sites further refine cell‐type specificity and immune compatibility. Hybrid and emerging platforms offer additional flexibility. Fourth‐generation VLPs achieved transient yet efficient RNP delivery to liver and retina, minimizing off‐target exposure, while engineered exosomes, including plant‐derived exosomes, enable selective cargo loading with low immunogenicity, supporting their potential for repeat dosing [505]. Engineered CRISPR–Cas9 variants (SpG and the SpRY) expand targetable PAM spaces and fit within single vector formats [506]. Fusion constructs such as Cas9TX link nuclease activity to exonuclease TREX2, reducing chromosomal translocations [507]. Catalytically inactive Cas13 fused to adenosine deaminase enables programmable RNA editing, introducing reversible, nonpermanent therapeutic options [508].

Looking forward, high‐throughput barcoded nanoparticle screens in NHPs, integrated with single‐cell sequencing, will identify vectors capable of targeting refractory populations such as hematopoietic stem cells and CNS neurons [509]. Incorporating synthetic riboswitches into viral genomes may enable pharmacological control of editor activity, facilitating repeatable and temporally precise interventions. Combining recombinase‐based PASTE systems with split‐intein AAVs could permit insertion of therapeutic cassettes up to 30 kb in a single dose [510]. Collectively, these efforts demonstrate a unifying principle: when chemistry, biology, and data science converge, delivery barriers recede, and the realization of curative genome editing moves within reach.

### 8.1 High‑Throughput and AI‑Guided Vector Engineering

Recent progress in genome‐editing therapeutics reflects the convergence of three synergistic innovations: barcoded in vivo nanoparticle screening, ML‐based performance prediction, and rapid design‐build‐test iteration. Together, these approaches have transformed the traditionally slow, empirical optimization process into a data‐driven, predictive, and scalable framework for clinical translation.

Traditionally, nanoparticle discovery followed a linear pipeline, progressing from in vitro assays to rodent validation and finally NHPs testing, and required large animal cohorts and years of optimization. DNA barcoding has transformed this workflow by enabling hundreds of chemically distinct LNPs, each tagged with a unique oligonucleotide identifier, to be pooled and evaluated within a single dose. Sequencing of barcodes recovered from target tissues provides a direct, quantitative measure of in vivo delivery efficiency for each formulation, revealing tissue‐specific tropism at unprecedented throughput. This strategy has identified LNPs capable of transfecting splenic endothelial cells, T cells, and hematopoietic stem cells, populations historically refractory to systemic delivery [511, 512]. Furthermore, the resulting barcoded datasets now support interspecies comparisons of delivery performance, streamlining candidate selection for translational development while reducing animal use [513].

The massive datasets generated by such screens are ideally suited for ML‐based analysis. Early neural‐network models trained on mutagenized AAV capsid libraries uncovered sequence determinants of packaging efficiency and organ tropism. These frameworks have since extended to nonviral formulations, correlating physicochemical parameters, including ionizable‐lipid p*K*_a_, helper‐lipid charge, hydrodynamic diameter, and serum protein corona composition, with functional outcomes such as editing rate and mRNA expression. ML‐guided promoter‐library analyses have also produced cell‐state‐specific promoters exhibiting up to 1000‐fold activity difference between target and control cell states [514].Collectively, these models narrow the experimental search space, transforming nanoparticle discovery from brute‐force screening into hypothesis‐driven design. Integration of barcoding and ML has accelerated optimization cycles across delivery platforms. Iterative diversification of a four‐component LNP scaffold produced zwitterionic, hydroxyl‐rich, and bioreducible lipid variants that outperform the clinical MC‐3 formulation. The bioreducible tail‐branched lipid 306‐O12B achieved 38.5% *Angptl3* editing in the liver, approximately 12‐fold higher than MC‐3, after only a few optimization rounds [515]. Similarly, site‐specific sgRNA modifications combined with refined LNP chemistry enabled durable genome editing in Phase 1 patients at doses as low as 0.1 mg kg^−1^ [9]. The development of SORT LNPs, incorporating a fifth charged lipid, progressed from concept to effective lung and spleen delivery in under a year, guided by barcoded datasets and ML‐derived feature importance [254].

This paradigm extends beyond LNPs. VLPs have evolved from passive encapsidation to chemically inducible dimerization and riboswitch‐regulated release, achieving on‐target efficiencies comparable to LNPs while minimizing unintended DNA and RNA edits [110]. High‐throughput screening and computational modeling have likewise accelerated optimization of dual‐AAV prime‐editing systems [516, 517]. Beyond discovery speed, these methods enhance mechanistic understanding. Barcoded LNP libraries revealed that tuning helper‐lipid charge can shift biodistribution from ApoE‐mediated hepatic uptake toward ApoE‐independent pathways, involving albumin and vitronectin‐αvβ3 interactions [518]. ML models subsequently predicted which lipid chemistries favor such protein corona profiles, which were later validated in vivo [519]. This iterative cycle—data generation, predictive modeling, and experimental confirmation—has converted empirical heuristics into quantitative design principles. The translational impact of these innovations is already visible. Three LNP–RNA drugs have received regulatory approval, following accelerated development pipelines grounded in these technologies [520]. Two distinct LNP formulations achieved therapeutic *PCSK9* base editing in NHPs after a single systemic dose, outlining a rapid trajectory toward clinical translation [39]. As datasets expand in dimensionality and scale, ML‐integrated nanoparticle discovery is poised to yield next‐generation vectors with higher precision, broader tissue reach, and reduced development time.

In conclusion, barcoded in vivo screening, ML‐guided prediction, and iterative chemical optimization now constitute a self‐reinforcing cycle transforming the discovery of delivery vehicles for genome editing. By converting trial‐and‐error exploration into model‐driven design, these approaches accelerate translation from chemical synthesis to clinical proof‐of‐concept, broaden the spectrum of accessible tissues, and lay the foundation for safer, more predictable therapeutic editing.

### 8.2 Modular and Personalized Delivery Systems

Therapeutic genome editing is increasingly converging on “plug‑and‑play” delivery platforms, modular systems whose core components can be interchanged to match patient‐specific genotypes, disease locations, and immune profiles. Instead of constructing new vectors for each indication, these systems rely on stable chassis such as viral capsids, LNPs, polymers, or EVs, onto which disease‐specific guide RNAs, regulatory elements, or immune‐evasive features can be rapidly incorporated. Experimental and early clinical evidence now confirms that genetic, anatomical, and immunological parameters can be tuned independently without compromising efficacy.

Genotype‐specific adaptability is most advanced in hepatic applications, where a single LNP scaffold can deliver diverse genome editors. For instance, NTLA‑2001 employs a hepatotropic LNP carrying SpCas9 mRNA and a TTR‐targeting guide, achieving 87% serum TTR reduction after a 0.3 mg kg^−1^ dose in amyloidosis patients [9]. The same chassis can be instantly repurposed with new guides for other TTR variants or unrelated genes. VERVE‐101 and VERVE‐102 apply the same principle with BE targeting PCSK9, using predictive guide modifications to increase efficiency [454, 521]. Additionally, the use of preassembled RNP complexes enables straightforward genetic targeting, as the Cas protein remains invariant and only the guide sequence is exchanged, offering true “click‐in” specificity.

Tissue selectivity is achieved through adjustable surface chemistries or regulatory elements. GalNAc conjugation directs LNPs to hepatocytes, whereas SORT formulations retune the same backbone to the lung or spleen, enabling up to 88% genomic editing in lung tissue while limiting liver exposure [259, 522]. Viral vectors mirror this modularity: engineered AAV variants such as RGD‐inserted capsids enhance tropism toward brain or muscle [18]. Further refinement arises from tissue‐restricted promoters and microRNA‐responsive elements. For example, a neuron‐restricted *SMN1* promoter improves CNS safety [523], or miR‐122/miR‐183 motifs suppress unwanted hepatic or sensory‐neuron expression [433, 524]. These interchangeable regulatory modules act as genetic “filters” layered atop structural design.

Disease‐responsive and immune‐adaptive modules expand personalization. Stimuli‐responsive polymers or membrane‐coated particles restrict release to hypoxic, oxidative, or inflammatory microenvironments, while immune tuning is achieved via capsid selection, transient B‐cell depletion, or microRNA‐based detargeting of APCs. In silico HLA‐peptide mapping further guides synonymous codon optimization to reduce immunogenicity. Each element (including tropism, responsiveness, immune shielding) functions as an independent plug‐in that can be configured per patient.

Collectively, these strategies form a modular toolkit in which lipid composition, capsid design, promoters, and immune motifs operate as interoperable components. Standardized component libraries, predictive algorithms linking module combinations to disease traits, and flexible manufacturing pipelines will be essential for large‐scale personalization under GMP constraints. Ultimately, the convergence of modular architecture and patient‐specific customization transforms delivery design from bespoke reconstruction to adaptive assembly, bringing genome editing closer to a universally adaptable, precision‐medicine framework.

### 8.3 Integration With Precision Editors

The expanding suite of precision editors, including BEs, PEs, CRISPR‐associated transposases (CAST), and RNA editors, exhibits substantial diversity in size, composition, and mechanism. Effective translation of these tools depends on delivery platforms coengineered to their distinct biophysical requirements. Iterative codevelopment, matching the structural and kinetic profiles of editors with compatible vehicles, has become essential for advancing genome and transcriptome editing toward clinical use.

BEs, particularly adenine and cytosine variants, pose formidable size constraints: full‐length SpCas9–deaminase fusions (∼6–7 kb) exceed the 4.7 kb limit of rAAV. Two complementary strategies have addressed this challenge. Dual‐AAV systems employ trans‐splicing inteins to reconstitute the editor in vivo, achieving up to 38% editing in liver [89]. Alternatively, compact nucleases such as SaCas9 and Nme2Cas9 enable single‐AAV delivery at lower doses, reducing capsid‐specific immunity [525, 526]. Parallel advances in LNPs, optimized for ionizable lipid p*K*_a_, helper‐lipid charge, and ligand targeting, have enabled coencapsulation of ABE mRNA and sgRNA, achieving >60% PCSK9 disruption and a 69% LDL‐C reduction in NHPs at 1.5 mg kg^−1^, directly informing the VERVE‐101 trial [527].

PEs introduce new design challenges arising from the instability of the pegRNA. Structural stabilization through 3′ pseudoknots or 2′‐O‐Me/PS linkages prolongs half‐life but alters charge and size, necessitating vehicle re‐engineering [528, 529]. Refined LNP formulations with higher RNA: lipid ratios now permit codelivery of PE mRNA and pegRNA to murine liver with >10% editing [530]. When payload size remains limiting, dual‐AAV strategies and split‐intein designs yield ∼42% editing at the *Dnmt1* locus, while VLPs provide transient, genome‐free PE RNP delivery, achieving therapeutic correction without persistent editor expression [124, 336].

CAST systems, which combine a CRISPR module with large transposase operons, demand multipartite carrier architectures. Dual‐ or triple‐AAV “split‐gene” approaches reconstitute the CAST components in situ, while capsid tropism can be tuned for liver or CNS targets. Nonviral solutions, such as phenylboronic acid‐grafted PEI polymeric nanoparticles cloaked with macrophage membranes, enable inflammation‐responsive CAST delivery within oxidative microenvironments [531]. These efforts highlight how the substantial size and complex stoichiometry of CAST are catalyzing next‐generation modular vehicle design.

RNA editors, exploiting ADAR or Cas13 enzymes, require smaller cargos and thus permit ultrasmall vehicles [532]. Exosome‐based carriers loaded with dCas9–VP64 mRNA or ADAR‐recruiting mRNAs restore hepatic or neuronal function in animal models [108], while MS2–aptamer‐guided VLPs deliver multiple RNA editor copies with minimal immunogenicity [362]. PBAE nanoparticles have further demonstrated aerosolizable delivery of mRNA to lung epithelium, suggesting translational potential for respiratory RNA‐editing [533].

Collectively, these advances illustrate that precision editing and delivery design are inseparable disciplines. Reducing editor size, stabilizing RNA components, and controlling protein persistence directly alleviate delivery constraints, while innovations in capsid evolution, lipid chemistry, and polymer responsiveness expand the therapeutic landscape. As emerging systems, such as PASTE integrases, epigenetic modulators, and RNA BEs, enter development, synchronized optimization of the editor and its carrier will remain central to realizing safe, durable, and clinically scalable genome‐editing therapies.

### 8.4 Immune‑Evasive and Redosable Platforms

Repeated dosing of genome‐editing vectors remains constrained by robust humoral and cellular immunity directed against both viral capsids and bacterial nucleases. Pre‐existing NAbs to natural AAV serotypes are present in 30–60% of humans and rise sharply after a single infusion, precluding redosing [534]. Analogous responses develop against protein components translated from LNPs or VLPs payloads. Current efforts converge on three complementary strategies: stealth capsids, tolerogenic or camouflaged surface modifications, and precisely timed transient immunosuppression, to reopen the therapeutic window for repeat administration.

Stealth and immune‐orthogonal capsids are engineered to preserve tropism while minimizing recognition by pre‐existing antibodies. Structure‐guided mutagenesis and in vivo selection have produced variants with substitutions at dominant B‐cell epitopes and de‐immunized surface loops. A liver‐tropic capsid administered at only 5 × 10^11^ vg kg^−1^ achieved a mean FIX activity of 33.7% (range 14–81%) without prophylactic steroids; only two of 10 recipients required brief treatment for transient transaminase elevations [535]. These lower doses reduce antigen exposure and antibody maturation, improving redosing potential. In NHPs, sequential dosing with orthogonal serotypes (e.g., AAV5 to AAV1) demonstrated efficient transduction, suggesting that a library of mutually immune‐distinct vectors could support chronic or repeated editing regimens [536].

Camouflaged and tolerogenic surfaces further blunt adaptive responses. Encapsulating AAV within EVs shields capsids from circulating Nabs while enabling surface modification with immunoregulatory ligands such as CTLA‐4 [369]. Vesicular encapsulation also retunes tropism through altered glycocalyx composition, now optimized by glycan editing and membrane‐protein swapping to target refractory tissues such as skeletal muscle and the CNS [537].

In parallel, synthetic polymers bearing zwitterionic or pegylated side chains suppress complement activation [538], while rapamycin‐loaded nanoparticle coatings promote hepatic Treg induction [539]. Additionally, plant‐derived exosomes, inherently nonimmunogenic in mammals, are also emerging as stealth scaffolds for CRISPR–RNP delivery [540].

Despite these advances, the possibility of breakthrough immunity necessitates transient immunosuppressive regimens as a critical adjunct. Combined regimens of rituximab‐mediated B‐cell depletion, low‐dose corticosteroids, and mTOR inhibition effectively dampen antibody and cytotoxic T‐cell responses in ocular and hepatic gene therapy trials [541, 542]. For patients with high baseline antibody titers, single infusions of IgG‐cleaving enzymes such as imlifidase (IdeS) remove >95% of circulating IgG within hours, restoring transduction efficiency during same‐serotype redosing in NHPs. The effect is reversible within 1–2 weeks, and optimal efficacy occurs when enzyme infusion precedes vector delivery by ∼24 h, minimizing complement activation [543].

Nonviral delivery platforms offer further flexibility for redosing. Fully synthetic LNPs, lacking intrinsic capsid antigens, can be readministered provided innate immune activation from ionizable lipids is controlled [544]. In NHPs, two systemic infusions of LNPs carrying mRNA and sgRNA achieved consecutive base editing of PCSK9 without loss of efficacy, an outcome not currently feasible with existing viral systems [40]. Similarly, VLPs assembled from self‐proteins such as PEG10 provide a low‐immunogenicity profile while delivering preformed Cas–sgRNA complexes that degrade within 24 h, minimizing persistent antigen exposure.

Together, these approaches: stealth vector engineering, immunomodulatory surface chemistry, and precisely timed immunosuppression, are redefining redosable genome editing. The key remaining challenges are to optimize the balance between antigenicity and tropism, standardize vesicle and coating manufacture for clinical scale, and refine transient immunosuppressive protocols to minimize systemic risk. Overcoming these barriers will extend genome editing from a single‐use curative intervention to an iterative, adaptive therapeutic platform, capable of accommodating evolving disease states and emerging pathogenic alleles.

### 8.5 Convergence With Small‑Molecule and Biologic Modalities

Combination regimens that integrate editing with established pharmacological and biologic therapies are emerging as a new paradigm in precision medicine. By pairing the durability of permanent genetic correction with the tunable kinetics of transient agents, such strategies can enhance both efficacy and controllability. Among these, RNA interference (RNAi), monoclonal antibodies (mAbs), and small‐molecule modulators exemplify how cross‐modality designs expand the therapeutic and delivery space of therapeutic genome editing.

RNAi provides an immediate suppressive effect that bridges the lag between vector administration and maximal editing. In the clinical management of TTR amyloidosis, the siRNA drug patisiran achieves rapid knockdown but requires lifelong infusions [545], whereas the LNP–CRISPR candidate NTLA‐2001 produces an 87% mean TTR reduction within 28 days at a 0.3 mg kg^−^^1^ dose and durable suppression in NHPs without detectable off‐target edits [9]. Sequential use of RNAi and CRISPR could therefore provide both immediate and permanent control, enabling discontinuation of chronic RNAi dosing once editing stabilizes. Similar “bridging” concepts apply in SCD, where transient BCL11A RNAi elevates HbF while BEs establish lasting HbF‐inducing mutations [546]. Preclinical coformulations of aptamer and siRNA conjugates with CRISPR RNPs in shared nanoparticle scaffolds further demonstrate the feasibility of single‐platform delivery for dual genetic interventions.

mAbs and ligands enhance tissue specificity by directing vectors to otherwise inaccessible sites. Antibody fragments targeting transferrin receptor CD71 extended muscle gene silencing for 4 weeks in NHPs after a single administration, outperforming unmodified oligonucleotide [547]. When coupled to AAV or LNP carriers encoding genome editing cassettes, such as dual AAV systems for dystrophin restoration in exon‐44‐deleted Duchenne models, the antibody moiety concentrates editing within muscle fibers, reducing systemic exposure [548]. RNA aptamers with antibody‐like binding, such as those recognizing platelet‐derived growth factor receptor α, have similarly delivered STAT3‐specific siRNA, underscoring the adaptability of ligand‐directed systems [549].

Small‐molecule modulators introduce chemical precision to regulate either disease pathways or vector activity. In one study, macrophage‐membrane‐coated PBAE nanoparticles carrying a CRISPR plasmid incorporated ROS‐sensitive trimethoxyphenyl linkers that triggered uptake and localized gene editing within inflamed tissue. Such conditional activation can be paired with oral pathway inhibitors that elevate ROS, restricting editing to diseased microenvironments [550]. In liver fibrosis models, hepatocyte‐derived exosomes loaded with dCas9–VP64 activators to upregulate *HNF4α*, combined with antifibrotic small molecules, produced superior restoration of hepatic function compared with either agent alone [108]. These examples highlight how integrating chemical control with genetic precision mitigates off‐target exposure and enhances spatial selectivity.

The rationale for combination therapy extends beyond additive efficacy. Transient RNAi or pharmacological suppression can restrain residual pathogenic cells left unedited, preventing relapse as edited populations expand. Adjustable dosing of antibodies or small molecules provides clinicians with postediting control over therapeutic intensity, helping to mitigate potential on‐target toxicity. Moreover, synergistic coadministration can reduce the required vector dose, thereby lowering immunogenic risk and manufacturing burden. Despite these advantages, cross‐modality design introduces challenges: mismatched pharmacokinetics complicate formulation (e.g., LNP–mRNA half‐lives of hours versus multiweek antibody persistence), regulatory oversight must address mixed‐modality safety, and codelivery of viral vectors with biologics may amplify immune responses, necessitating refined immunosuppressive protocols.

Looking ahead, modular carriers such as SEND and VLPs offer frameworks for the copackaging of mRNA, sgRNA, and targeting ligands. Adjusting helper‐lipid charge already enables vitronectin‐guided LNP trafficking to lung endothelium; similar modifications may accommodate antibody or small‐molecule cargoes. As vector engineering and clinical validation progress, genome editing is poised to transition from stand‐alone interventions to integrated therapeutic regimens, where RNAi, antibodies, and small molecules act as complementary partners, each offsetting the limitations of the others to achieve safer, more adaptable, and durable cures.

## 9 Conclusions and Outlook

Decades of progress have transformed genome editors from research tools into clinically deployed therapeutics, yet every translational breakthrough has required parallel advances in delivery. Compared with nuclease engineering, delivery remains the dominant determinant of efficacy, durability, and safety. Recent trials underscore this dependence: LNP‐mediated delivery of CRISPR–Cas9 mRNA and sgRNA lowered circulating TTR protein levels by 87% following a single NTLA‐2001 infusion [9], while a 0.6 mg kg^−^^1^ dose of VERVE‑101 reduced LDL‐C by 55% in patients with heterozygous FH [551]. Similarly, dual AAV9‐mediated delivery of split BEs has achieved up to 38% editing in murine liver and measurable benefit in progeria, Niemann‐Pick disease, and DMD [89, 552, 553]. Each of these clinical and preclinical successes is inseparable from the underlying delivery strategy.

Our analysis reveals that while genome editing tools have achieved remarkable sophistication, their clinical translation is fundamentally constrained by delivery challenges. The current landscape demonstrates a clear hierarchy where delivery efficiency directly dictates therapeutic outcomes. Viral vectors provide high efficiency but face biological constraints, while nonviral systems offer superior safety profiles but require further optimization for broader tissue targeting. The most promising developments emerge from hybrid approaches that seek to combine the advantages of multiple platforms.

Viral vectors illustrate both the potential and limitations of biological carriers. Native AAV serotypes combine favorable safety with limited cargo capacity (<4.7 kb), pre‐existing NAbs, and dose‐dependent hepatotoxicity. Directed evolution, peptide display, and regulatory‐element tuning have improved skeletal‐muscle transduction by 10–29‐fold and reduced off‐target hepatic or APC expression through miR‐122a/miR‐142 detargeting [18, 554, 555]. Nonetheless, therapeutic dosing typically requires 10^15^–10^16^ vector genomes per patient, straining manufacturing capacity and amplifying immunologic risks.

Nonviral systems, particularly LNPs, demonstrate how chemical engineering can redefine organ tropism. Iterative tuning of ionizable lipid chemistry has reduced the effective hepatocyte‐silencing dose from 1 to 0.005 mg kg^−1^ in mouse models [556], while GalNAc conjugation directs uptake via the asialoglycoprotein‐receptor and SORT LNPs enable editing in the lung or spleen [259, 557]. DNA‐barcoded lipid libraries now identify variants capable of editing hematopoietic stem cells in NHPs, collectively dispelling the notion that LNPs are strictly hepatotropic [558].

Emerging platforms aim to combine the efficiency of viral vectors with the transient safety profile of nanoparticles. Engineered VLPs derived from MLV Gag deliver base‐editor RNPs and achieve 63% Pcsk9 editing in mouse liver after a single intravenous dose [110]. Exosomes exploiting endogenous secretion pathways have carried dCas9–VPR activators to hepatocytes to rescue fibrotic phenotypes [384]. In addition, hydroxyl‐rich, charge‐switching, or pH‐responsive polymeric nanoparticles are beginning to reach vascular, pulmonary, and oncologic compartments in small‐animal models [297, 559]. Although editing efficiencies remain modest relative to viral or LNP systems, their low immunogenicity and inherent redosability position them as attractive options for chronic indications.

Beyond carriers, chemical optimization of editing components reinforces delivery's central importance. Guide RNAs bearing 2′‑O‑methyl or 2′‑fluoro modification exhibit increased nuclease resistance and higher editing efficiency in vivo [560, 561]. Combined viral–nonviral regimens, such as AAV templates paired with LNP‐borne nucleases, have enabled HDR in otherwise refractory postmitotic tissues, illustrating the growing permeability between delivery modalities.

Looking forward, we identify three critical frontiers for delivery system development: (1) achieving cell‐type‐specific targeting through novel ligand discovery and computational design; (2) overcoming intracellular barriers including endosomal escape and nuclear import; (3) developing scalable manufacturing processes for complex vector systems. The integration of ML approaches with high‐throughput screening platforms will accelerate vector optimization, while advances in biomaterials science may yield entirely new delivery paradigms.

Collectively, current evidence asserts that sophisticated editors cannot compensate for inadequate delivery. Achieving safe and equitable genome editing will require continuous innovation across molecular biology, material science, immunology, data analytics, and biomanufacturing. Multidisciplinary consortia are already accelerating capsid evolution, high‐throughput nanoparticle screening, and automated vector production. Broader collaboration remains essential: harmonized preclinical pipelines, shared reagent libraries, and open‐access datasets will streamline iteration and minimize redundancy, while early regulatory engagement will expedite translation without compromising safety. Ultimately, the ability to engineer next‐generation vectors that are potent, precise, and manufacturable at scale will determine whether genome editing realizes its curative promise. Delivery is therefore not merely a supporting technology, but the linchpin that will shape the clinical future of genome editing, necessitating a sustained, integrative commitment to scientific and translational advancement.

## Author Contributions

M.Y., R.X., and J.Z. proposed this theme, organized the structure, and made the decision to submit it for publication. M.Y., Y.S., and Z.W. drafted the manuscript, prepared the figures, and selected the cited references. K.C., L.L., X.Z., X.D., and C.Y. revised the details of this review. R.X. and J.Z. approved the final version of manuscript. All authors have read and approved the final manuscript.

## Ethics Statement

No ethical approval was required for this study.

## Conflicts of Interest

The authors declare no conflicts of interest.

## Data Availability

The authors have nothing to report.
